# Short, Enantioselective Total Synthesis of (+)-Ineleganolide

**DOI:** 10.1021/jacs.5c17640

**Published:** 2025-11-21

**Authors:** Kuan Yu, Aikaterini Gorou, Danny Huang, Thomas J. Maimone

**Affiliations:** Department of Chemistry, 1438 University of California—Berkeley, 826 Latimer Hall, Berkeley, California 94720, United States

## Abstract

Owing to their distinctive polycyclic architectures and promising bioactivities, the large family of furanocembranoid natural products continues to attract significant attention from the synthetic community. Herein, we present a 10-step total synthesis of (+)-ineleganolide, a highly oxidized norcembranoid natural product featuring a synthetically challenging caged pentacyclic framework. An interesting *trans*-selective photochemical [2 + 2] cycloaddition involving an unusual *α,β*-unsaturated tricarbonyl chromophore was used to generate a strained cyclobutanol which underwent a C–C bond cleavage cascade when exposed to Brønsted acid. Through this sequence, the ineleganolide core polycycle was generated in only six steps from commercially available materials. This strategy provides a conceptually new abiotic blueprint for this fascinating family of diterpenes.

## Graphical abstract

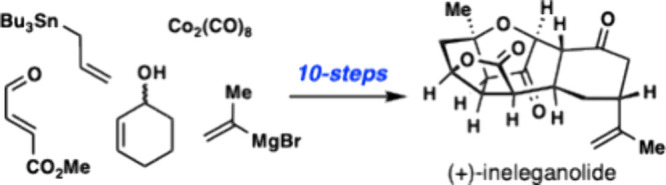

Cembranoid and norcembranoid natural products of marine origin have attracted considerable synthetic and biological attention over the past three decades.^1^ This has been driven by their structural complexity and diversity, mysterious biosynthetic origins, and promising biological properties.^2^ The polycyclic family of furanocembranoids, including (−)-scabrolide B (**3**),^3^ (+)-ineleganolide (**4**),^4^ (+)-verrillin (**5**),^5^ and (−)-bielschowskysin (**6**),^6^ has received heightened attention in recent times (Figure 1A). Presumably stemming from transannular ring closures and oxidative modifications of furan-derived macrocyclic precursors, structures exemplified by (−)-bipinnatin J (**1**)^7^ and 5*-epi*-sinuleptolide (**2**),^8^ bioinspired approaches have dominated synthetic efforts toward many of these targets.^1,2^

**Figure 1:**
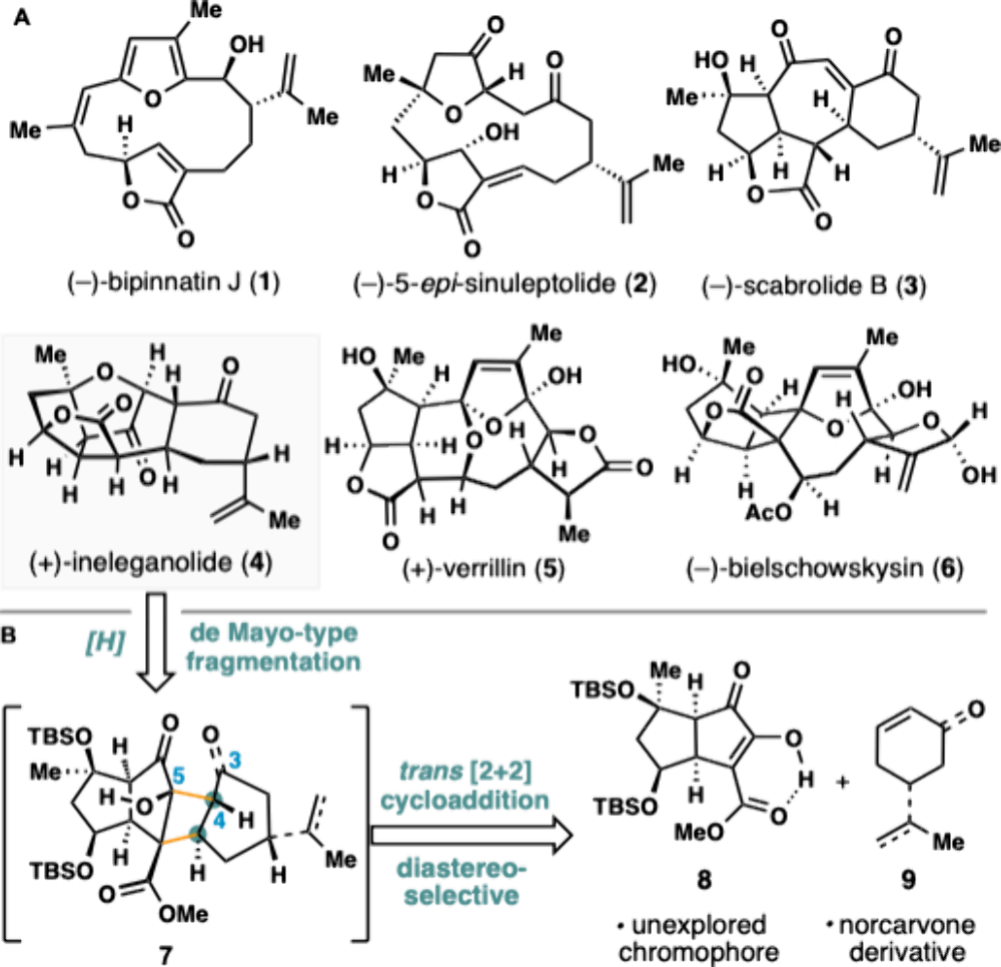
(A) Structures of select furanocembranoid natural products. (B) General retrosynthetic plan for ineleganolide.

(+)-Ineleganolide (**4**), first isolated from the dichloromethane extracts of the soft coral *Sinularia inelegans* by Duh and co-workers in 1999, remains a flagship norcembranoid and cherished synthetic target.^4^ Despite preliminary biological studies documenting cytotoxicity against P-388 murine leukemia cell lines, the targets of **4**, and indeed most furanocembranoids, are poorly understood.^9^ Harboring a densely oxygenated, pentacyclic framework of high topological complexity (including eight contiguous stereocenters), total syntheses of **4** remained elusive until only recently.^10^

In 2011, Pattenden and co-workers established a potential biosynthetic link between **4** and (−)-5*-epi*-sinuleptolide (**2**) wherein treatment of acetylated **2** with lithium bis­(trimethylsilyl)­amide (LiHMDS) triggered a double-transannular Michael cyclization, successfully achieving a semisynthesis of (+)-ineleganolide (**4**) albeit in low yield.^11^ In 2022, Wood and co-workers reported the first total synthesis of (+)-ineleganolide (**4**) in 22 steps inspired by Pattenden’s transannular Michael addition approach, but using an NHK macrocyclization of a linalool-derived staring material^12^ to access a macrocyclic precursor.^13^ Building upon their long-standing interest and efforts in this area,^10c–10g^ Stoltz and co-workers achieved a convergent synthesis of **4** in 2023, utilizing a one-step Michael/aldol cascade and a SmI_2_-mediated semipinacol rearrangement to assemble the rigid cycloheptane ring.^14^ Additional biosynthetic insight into **4** and congeners came from a recent 2024 report by Fürstner and co-workers who disclosed that (+)-ineleganolide (**4**) can be produced by treating (−)-scabrolide B (**3**) with base, albeit in low yield.^15^ Contemporaneous studies by Sarlah reached the same insightful conclusion and ultimately led to an optimized synthetic protocol and 14-step total synthesis of **4**.^16^ Given our past interest in developing efficient, abiotic approaches to cyclized cembranoids,^17^ we also embarked on a total synthesis of **4**. Herein we report a 10-step (asymmetric) or 9-step (formal racemic) solution to this challenging problem in polycyclic terpene synthesis.

While enolate chemistry has guided many of the synthetic strategies used to prepare polycyclic furanocembranoids, we were inspired by a de Mayo-type process involving an alternative intermolecular photochemical [2 + 2] cycloaddition/fragmentation (Figure 1B).^18^ In combination with a reduction, **4** could thus be traced back to strained cyclobutane **7** by using this approach. This cycloadduct could in turn arise from the merger of 5,5-fused bicycle **8**—possessing a hitherto unknown chromophore for photochemical [2 + 2] cycloadditions—and norcarvone or a suitable cyclohexene-containing building block (see **9**). There are several regio- and diastereoselectivity challenges inherent to this overall strategy. Photochemical [2 + 2] cycloadditions between cyclopentenone-containing chromophores and cyclohexenes are known to produce both *cis-* and *trans-*cyclobutane adducts, indicative of a stepwise pathway.^19^ Typically, unpredictable mixtures of isomers are obtained, and *trans*-selective processes are rare.^20^ While a *trans*-selective process (directly forming **7**) appeared best for our specific synthetic goals, we had assumed that a *cis-*[2 + 2] cycloaddition in conjunction with a late-stage epimerization of the C4 position would also be viable. In addition, when a ketone is present at C3, intermediates such as **7** have an additional, undesired mode of C–C bond cleavage, namely, cleavage of the C4–C5 bond. All of these selectivity issues needed to be considered in realizing this general strategy.

Our investigations commenced with a catalytic asymmetric Keck allylation^21^ of commercially available aldehyde **10** with allyltributyltin, furnishing allylic alcohol **11** in 93% yield (99% ee) and with an (*S*)-configuration at C10, the first crucial stereocenter set (Scheme 1). The terminal alkene was then oxidized to β-hydroxyketone **12**. While traditional Tsuji–Wacker oxidation conditions (PdCl_2_, CuCl, O_2_, DMF/H_2_O) did provide **12** in 30% yield, lactol products derived from the undesired anti-Markovnikov addition were also isolated in equimolar quantity. After extensive screening, Sigman’s modification,^22^ involving a combination of Pd[(−)-sparteine]­Cl_2_, *N,N′*-dimethylacetamide (DMAc), and H_2_O under oxygen atmosphere, was found to provide an optimal outcome, generating **12** in 67% yield. Of note, **12** can also be prepared racemically in just one step from **10** (see the Supporting Information (SI)). Having β-hydroxyketone **12** secured, our attention turned toward setting the C8 stereochemistry in preparation for a Pauson–Khand reaction to assemble the 5,5-fused core present in **8**. Lithium ethoxyacetylide was then added to **12**, and after protection of the unstable diol as a *tert*-butyl­(dimethyl)­silyl (TBS) ether, **13** and its C8 epimer **13′** (not shown, see the SI) were formed in 30% and 45% yield, respectively. The observed low diastereoselectivity was attributed to a competing interplay between a closed, chelation-controlled transition state (see TS-1, Scheme 1 inset) and an open one (see TS-2). Further comprehensive investigation into the effects of additives, temperature, concentration, and solvents^23^ led to no appreciable improvement. In contrast, a markedly enhanced diastereoselectivity ratio (dr, 9:1) with higher combined yield (92%) for undesired stereoisomer **13′** was observed when TiCl_4_ was used as the chelating metal source^24^ (see Scheme 1 insert). Preferential formation of **13** was ultimately achieved by *in situ* generation of a TBS silyl ether prior to lithiate addition, producing enynes **13** and **13′** in 66% and 22% yield, respectively. These 1,2-addition studies are insightful in the broader context of furanocembranoid total synthesis; they demonstrate a pathway to selectively obtain either ineleganolide (**4**) or bielschowskysin (**6**) stereochemistry at C8 from a common synthetic intermediate, a feat not currently possible using chiral pool-derived cyclopentenone building blocks.^25^

**Scheme 1:**
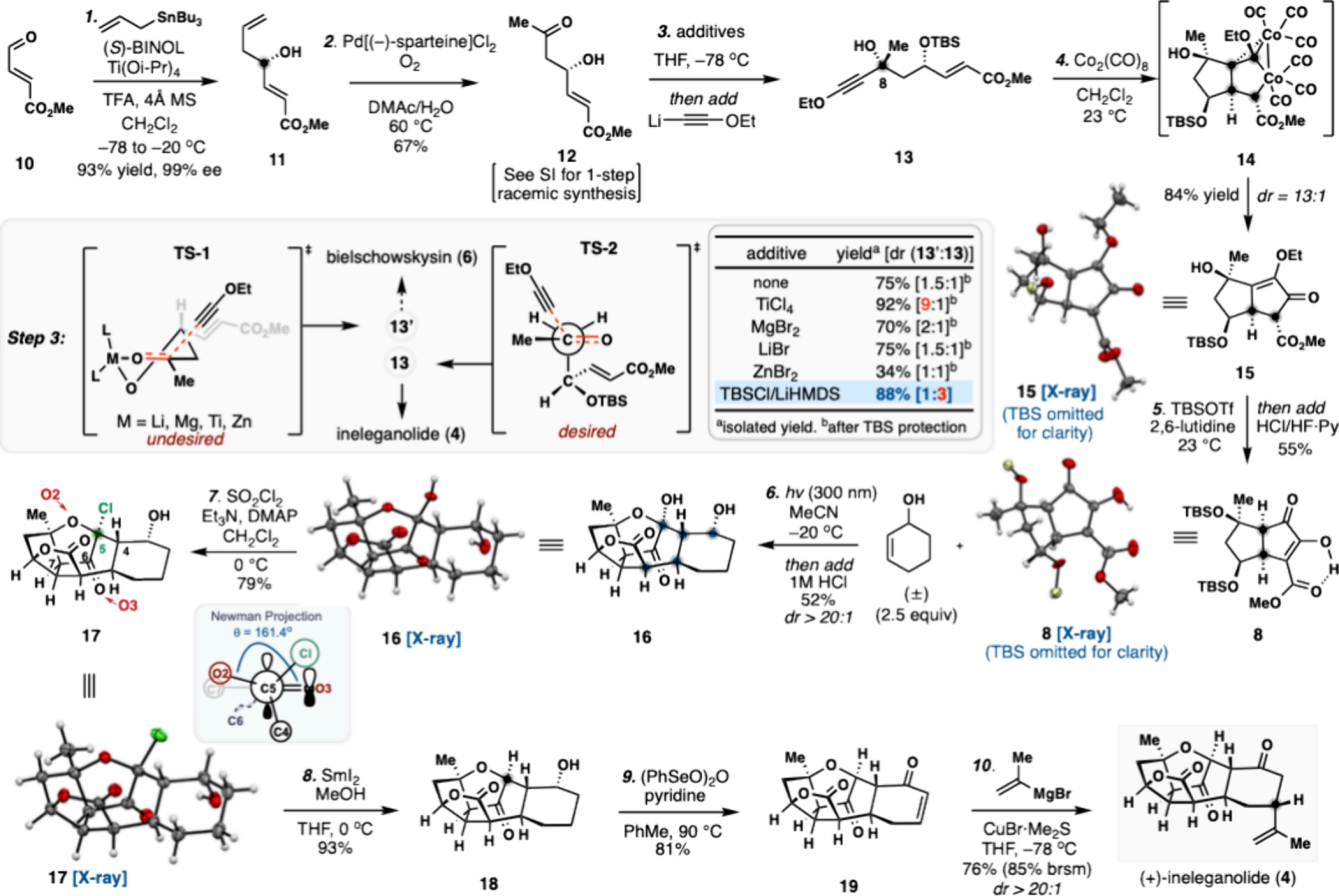
Total Synthesis of (+)-Ineleganolide (**4**)

With a three-step asymmetric synthesis of enyne **13** in hand, we proceeded to evaluate an intramolecular Pauson–Khand (PK) reaction to form bicycle **15**. We anticipated that achieving the desired *endo* cyclization mode needed in our system would be challenging as opposite *exo*/*endo* selectivities are generally observed between substrates bearing bulky allylic siloxy groups and their free alcohol counterparts—while the former typically favors *exo* selectivity, the latter delivers the *endo* cycloadducts predominantly.^26^ To our surprise, we found that the allylic TBS silyl ether-containing substrate **13** underwent PK cyclization smoothly in the presence of Co_2_(CO)_8_, affording desired product **15** and its C11, C12-epimer **15′** (not shown, see the SI) in an 84% combined yield with excellent *endo* selectivity (13:1 ratio favoring **15**). The unusual stereoselectivity could be ascribed to minimization of steric repulsion between the C8 substituents and the ethoxy unit in the endo transition state (see **14**). The structure and absolute configuration of **15** were unambiguously confirmed by X-ray crystallographic analysis. From **15**, unveiling the key enone-enol-ester chromophore found in **8** proved to be challenging. In a carefully optimized sequence, the addition of TBSOTf and 2,6-lutidine to **15** both silylated the C8 tertiary alcohol and formed an unstable cyclopentadiene intermediate via silyl enol ether formation at C5. Subsequent exposure of the resulting reaction mixture was observed to *aq*. HCl (6 M) followed by a HF·pyridine complex (HF·Py) delivered carbonyl isomerization product **8** in synthetically useful yields (55%). The structure of **8** was confirmed unambiguously by an X-ray crystallographic analysis.

With bicyclic enol **8** in hand, we sought to assess its viability as a coupling partner in an intermolecular photochemical [2 + 2] cycloaddition with a suitable cyclohexene-containing fragment. After thorough evaluation of an array of cyclohexene-containing substrates and photochemical conditions (see Table 1), we were delighted to find that *trans* adduct **20** and *cis* adduct **21** were produced from **8** and racemic 2-cyclohexen-1-ol (2.5 equiv) in 43% and 29% yield, respectively (entry 8, Table 1). Although both **20** and **21** were found to be unstable under basic, acidic, and thermal conditions, both were rapidly characterized by ^1^H NMR and ^13^C NMR (including 2D). Notably, both cycloadducts contain the desired regioselectivity, facial selectivity, and *exo* selectivity. Moreover, a single configuration at the C3 stereocenter was obtained, despite using racemic 2-cyclohexen-1-ol. Further exploration of reaction solvents revealed that acetonitrile (MeCN) was optimal for achieving uncommon *trans*-selectivity.^19a,27^

**Table 1:**
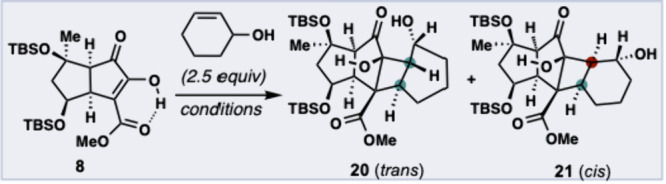

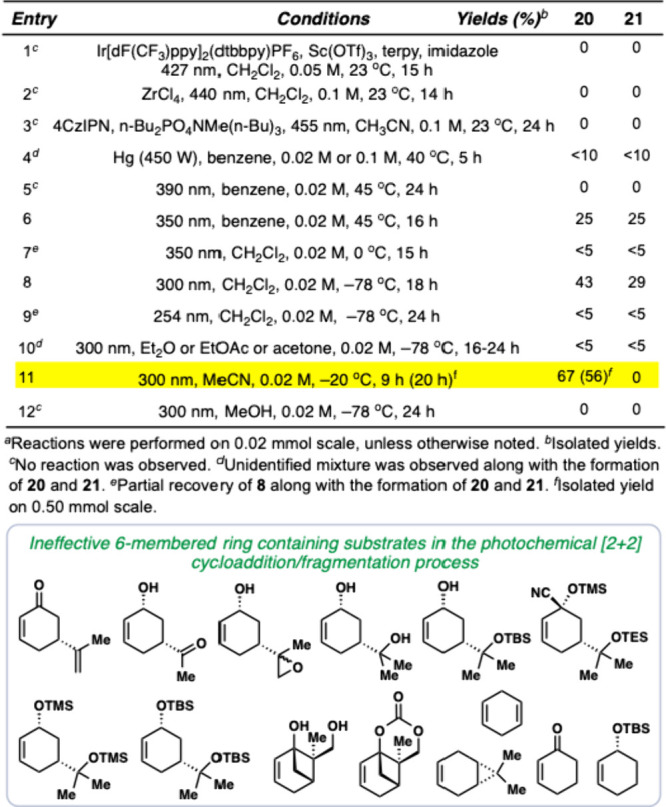
Optimization of the Photochemical *trans*-[2 + 2] Cycloaddition^a^ ^,^ ^b^ ^,^ ^c^ ^,^ ^d^ ^,^ ^e^ ^,^ ^f^

a Reactions were performed on 0.02 mmol scale, unless otherwise noted.

b Isolated yields.

c No reaction was observed.

d Unidentified mixture was observed along with the formation of **20** and **21**.

e Partial recovery of 8 along with the formation of **20** and **21**.

f Isolated yield on 0.50 mmol scale.

Having established a reliable route for preparing **20**, we executed the pivotal fragmentation step of the de Mayo-type process (Scheme 1). Surprisingly, directly treating the optimized photocycloaddition reaction mixture with *aq*. HCl (1 M) led to the spontaneous formation of caged pentacycle **16** in 52% yield and as essentially a single diastereomer. The structure of compound **16** was confirmed by X-ray analysis. Presumably, this cascade involves desilylation of the C10 and C8 hydroxyl groups with concomitant formation of the hallmark C19 lactone, retro-aldol cleavage of the C5–C12 bond, and finally ketalization to furnish the pentacycle (Figure 2A). Thus, in a single step, the entire skeleton and eight of nine vital stereocenters of ineleganolide (**4**) were assembled from **8** and (±)-2-cyclohexen-1-ol.

**Figure 2:**
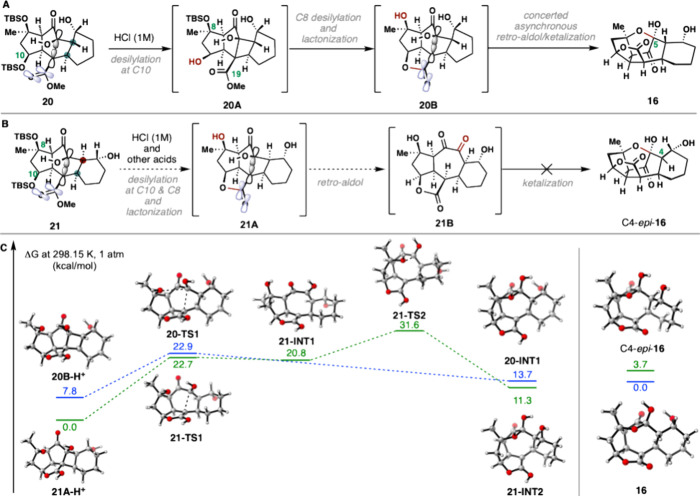
(A) Proposed fragmentation process for **20**. (B) Proposed decomposition process for **21**. (C) The free energy profile for the proposed fragmentation process of **20** and **21** at the M062X-D3/def2-TZVP/SMD­(MeCN) level of theory.

Later investigation revealed that treatment of the C4-*epi*-isomer **21** with various acids (i.e., *aq*. HCl, HF, HF·Py, and TFA) only resulted in slow decomposition (Figure 2B), highlighting the importance of obtaining the *trans*-[2 + 2] adduct in the realization of this synthetic strategy. This observation was also examined by DFT calculations and is illustrated in Figure 2C. While the retro-aldol and ketalization were calculated to occur as two distinct steps through intermediate cycloheptadione **21-INT1** for the *cis* isomer **21**, we were able to locate a transition state directly connecting protonated cyclobutanol (**20B–H** ^**+**^) to lactol **20-INT1**, supported by intrinsic reaction coordinate (IRC) calculations (see the SI).^28^ Although we had originally anticipated that **21-INT1** would undergo facile epimerization to the desired *trans*-fused isomer under strongly acidic reaction conditions, with this additional insight into the potential energy surface, we instead propose that the cycloheptadione intermediate was prone to undesired acid-mediated side reactivities, an issue fortuitously bypassed by the concerted asynchronous retro-aldol/ketalization sequence exhibited by the *trans*-fused isomer.

With robust, six-step access to pentacycle **16** secured, the stage was set for the regioselective removal of the extraneous C5 bridgehead hydroxyl group in the presence of the C3 secondary alcohol (Scheme 1). Initial attempts to elicit direct ionic hydrogenation or SmI_2_-mediated dehydroxylation resulted only in the recovery of **16** or formation of unidentified mixtures. While a Barton–McCombie-type deoxygenation might allow for a broader reactivity window to be explored, we were unable to form the requisite activated thiocarbonyls likely due to steric hindrance at this position. As a final recourse, we explored the conversion of **16** into its corresponding cyclic sulfate using sulfuryl chloride (SO_2_Cl_2_) in the hopes of leveraging the potentially more accessible C3 hydroxyl group as a tether. To our surprise, this process instead formed chloride **17**, whose structure was established by X-ray crystallographic analysis, with the C3 alcohol intact. Chloride **17** was then rapidly elaborated to (+)-ineleganolide (**4**) via a three-step endgame. The chlorine atom was uneventfully removed under careful SmI_2_-mediated dehalogenation conditions, affording β-keto tetrahydrofuran **18** in 93% yield. Overreduction of the β-keto tetrahydrofuran moiety was not observed likely due to poor orbital overlap, i.e. the O3–C6–C5–O2 dihedral angle being 161.4° as noted in the X-ray crystal structure of **17** (see insert Scheme 1). With **18** secured, we were pleased to find that a one-pot double-oxidation process employing benzeneseleninic acid anhydride [(PhSeO)_2_O] efficiently delivered cyclohexenone **19** in 81% yield. Finally, the stereospecific installation of the C1 isopropenyl unit was explored. While Zhang and co-workers faced stereochemical challenges on a similar conjugate addition reaction to a seven-membered enone in their synthesis of (−)-sinulochmodin C,^29^ we fortuitously observed the opposite facial selectivity when enone **19** was treated with excess freshly prepared isopropenyl cuprate, which furnished (+)-ineleganolide (**4**) in 76% isolated yield with excellent diastereoselectivity (dr >20:1).

In summary, we accomplished a concise 10-step asymmetric total synthesis of ineleganolide (**4**), a flagship norcembranoid. Key to the success of this work were a diastereoselective alkynylation, an *endo* selective Pauson–Khand reaction, a stereospecific de Mayo-type intermolecular photochemical [2 + 2] cycloaddition/fragmentation cascade, a regioselective chlorination of a hindered lactol, and a facial selective conjugate addition. This synthetic strategy proceeded with good efficiency and high stereocontrol from readily available starting materials. Notably, our convergent coupling strategy requires only one of the two coupling partners to be generated in the enantioenriched form, thus obviating the need to prepare norcarvone or other chiral coupling fragments. Finally, we believe elements of this study can inform future syntheses of other intriguing cyclized cembranoids, such as bielschowskysin, which remains an unsolved synthetic problem. Such investigations are ongoing in our lab.

## Supplementary Material



## Notes

Experimental procedures, spectroscopic data for all compounds, and X-ray crystallographic data for **16**, **17**, **15**, **8** (CCDC 2491903–2491906). (PDF)
