# Supplementary material for: Short, Enantioselective Total Synthesis of (+)-Ineleganolide
Source: J Am Chem Soc. 2025 Nov 21;147(49):44727–32. doi: 10.1021/jacs.5c17640 (PMC12703658; doi:10.1021/jacs.5c17640)

Supporting Information for

Short, Enantioselective Total Synthesis of (+)-Ineleganolide

Kuan Yu,* Aikaterini Gorou,# Danny Huang,# and Thomas J. Maimone*

Department of Chemistry, University of California-Berkeley, 826 Latimer Hall

Berkeley,CA 94720 (USA)

**Corresponding author*

Email: maimone@berkeley.edu, yukuan@berkeley.edu

# these authors contributed equally

Supporting Information Available

I) **General Procedures**

II) **Experimental Procedures and Spectroscopic Data of Compounds**

III) **Natural Product Spectral Comparisons**

IV) **Computational Details and Coordinates of Computed Structures**

V) **Crystallographic Data**

VI) **Abbreviations**

VII) **References**

VIII) **^{1}H and ^{13}C NMR Spectra of Compounds**

I) General Procedures

All reactions were carried out under an argon atmosphere with dry solvents under anhydrous conditions, unless otherwise noted. Starting aldehyde **10** [cas no.: 5837-72-9] was purchased from AmBeed ($ 219.2 USD/g), and the ethyl ester [cas no.: 2960-66-9] is commercially available through Sigma-Aldrich for $ 11.5 USD/g. Air- and moisture-sensitive liquids were transferred via syringe. When indicated, solvents or reagents were degassed by sparging with argon for 10 minutes in an ultrasound bath at 25 ºC. Volatile solvents were removed under reduced pressure rotary evaporation. Analytical and preparative thin-layer chromatography (TLC) were performed using glass plates coated with silica gel (0.25-mm, 60-Å pore size, Merck TLC Silicagel 60 F254) impregnated with a fluorescent indicator (254 nm). TLC plates were visualized by exposure to ultraviolet light (UV) and then were stained by submersion in an ethanolic anisaldehyde solution followed by brief heating on a hot plate. Flash column chromatography was performed with silica gel purchased from Fisher Scientific (230-40 mesh, Grade 60). All samples were loaded onto flash columns either as solutions in EtOAc/hexanes, Et_2O/CH_2Cl_2, acetone/hexanes or acetone/CH_2Cl_2. Anhydrous methylene chloride (CH_2Cl_2), acetonitrile (MeCN), tetrahydrofuran (THF) and toluene (PhMe) were obtained by passing these previously degassed solvents through activated alumina columns. Anhydrous benzene, acetone, chloroform ($CHCl_3$), ethyl acetate (EtOAc), ethanol (EtOH), methanol (MeOH), *N, N'*-dimethylacetamide (DMAc) and triethylamine (Et_3N) were purchased at the highest commercial quality from Sigma Aldrich and used without further purification, unless otherwise stated. Proton nuclear magnetic resonance (^{1}H NMR) spectra and carbon nuclear magnetic resonance (^{13}C NMR) spectra were recorded on Bruker NEO501, AV600, and AV700 spectrometers at 23 ºC. Proton chemical shifts are expressed as parts per million (ppm, δ scale) and are referenced to residual solvent ($CHCl_3$, δ 7.26 (s), C_6D_5H δ 7.16 (s)). Carbon chemical shifts are expressed as parts per million (ppm, δ scale) and are referenced to the residual solvent ($CHCl_3$, δ 77.16, C_6D_6, δ 128.06). Data is presented as follows: chemical shift, multiplicity (s = singlet, d = doublet, dd = doublet of doublets, ddd = doublet of doublet of doublet, dt = triplet of doublet, t = triplet, q = quartet, m = multiplet, br = broad, coupling constant (*J*) in Hertz

(Hz), and integration. Infrared spectra were recorded on a Bruker Alpha FT-IR spectrometer as thin films and are reported as frequency of absorption (cm^{-1}). Only selected resonances are reported. High-resolution mass spectra (HRMS) were obtained by the mass spectrometry facility at the University of California, Berkeley using a Finnigan LTQFT mass spectrometer (Thermo Electron Corporation). Chiral HPLC analyses were performed on a Waters Alliance e2695 Separations Module using Chiralpak® columns (IB N-5) eluting with *i*-PrOH/hexanes mixtures as indicated. Detection of eluent was carried out with a photodiode array detector at 210 nm. Optical rotations were measured on a PerkinElmer 241 polarimeter using a 1 dm path-length cell at 589 nm. X-ray diffraction data was collected at the Small Molecule X-ray Crystallography Facility (CheXray) at the University of California, Berkeley using a Rigaku XtaLAB P200 equipped with a MicroMax 007HF rotating anoted an Pilatus3 R 200K-A hybrid pixel array detector. Data were collected using CuKa radiation (l = 1.5418 Å). Melting points for crystalline compounds were determined with a MEL-TEMP II apparatus and are uncorrected.

II) Experimental Procedures and Spectroscopic Data of Compounds

Table S1. One-step Syntheses of β-hydroxyketone 12[a]:

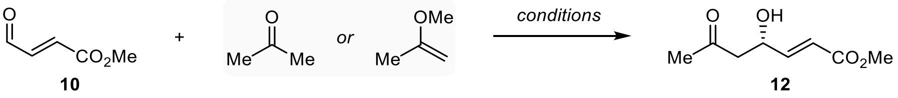

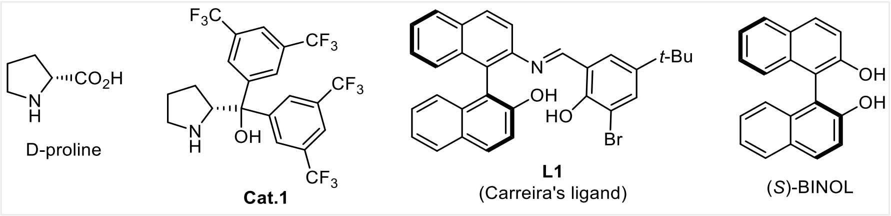

Entry	Conditions	Yield [%][b]	Ee [%][c]
1	LDA, acetone, THF, –78 °C, 1.5 h	64	0
2	D-proline, acetone/H_2O (19:1), 0.1 M, 23 °C, 12 h	24	0
3	D-proline, acetone/H_2O (19:1), 0.01 M, 23 °C, 24 h	10	0
4	D-proline, acetone/DMSO (1:4) 0.1 M, 23 °C, 12 h	32	30
5	D-proline, acetone/DMSO (30:1) 0.03 M, 23 °C, 12 h	25	30
6	D-proline, acetone, 0.01 M, 0 °C, 12 h	30	24
7	D-proline, acetone, 0.01 M, –78 °C, 12 h	13	30
8	**Cat. 1**, THF, 23 °C, 36 h	0	0
9	**Cat. 1**, DMSO, 23 °C, 5 days	0	0
10	$Ti(Oi\text{-}Pr)_4$, 2,6-Di-*tert*-butyl-4-methylpyridine, **L1** 2-methoxypropene, 0 °C, 14 h, *then* HCl (1 M, aq.), 10 min	36	49
11	$Ti(Oi\text{-}Pr)_4$, (*S*)-BINOL, 2-methoxypropene, 4Å MS CH_2Cl_2, –70 to –30 °C, 8 h, *then* HCl (1 M, aq.), 10 min	55	46
12	$Ti(Oi\text{-}Pr)_2Cl_2$, (*S*)-BINOL, 2-methoxypropene, 4Å MS CH_2Cl_2, –70 to –30 °C, 12 h, *then* HCl (1 M, aq.), 10 min	40	15

[a]Reactions were performed on 1.0 mmol scale. [b]Isolated yields. [c]Determined by chiral HPLC analysis.

Preparation of β-hydroxyketone (±)-12 (entry 1, Table S1):

To a flame-dried 10 mL reaction tube charged with diisopropylamine (0.16 mL, 1.1 mmol, 1.1 equiv) and THF (2 mL) at –78 °C was added *n*-BuLi (0.63 mL, 1.6 M in hexanes, 1.0 mmol, 1.0 equiv). The resulting mixture was stirred for 10 min before it was warmed to 0 °C and stirred for 10 min. The reaction tube was cooled to –78 °C before a solution of freshly distilled acetone (74 μL, 1.0 mmol, 1.0 equiv) in THF (1 mL) was added dropwise, and the reaction mixture was stirred for an additional 1 h. To the resulting mixture was added a

solution of aldehyde **10** (0.11 g, 1.0 mmol, 1.0 equiv) in THF (1 mL), and the reaction was stirred for another 10 min before it was quenched with NH_4Cl (3 mL, sat. aq.). The layers were separated, and the aqueous layer was extracted with EtOAc (6 × 3 mL). The combined organic layers were washed with brine (5 mL), dried (Na_2SO_4) and concentrated *in vacuo*. Flash column chromatography (silica gel, hexanes: EtOAc 2:1) afforded β-hydroxyketone (±)-**12** (0.11 g, 64%) as a colorless oil.

TLC: R_f = 0.30 (silica gel, hexanes: EtOAc 1:1).

^{1}H NMR (500 MHz, $CDCl_3$) δ = 6.88 (dd, J = 15.6, 4.1 Hz, 1H), 6.14 (dd, J = 15.6, 1.9 Hz, 1H), 4.79 – 4.72 (m, 1H), 3.74 (s, 3H), 3.26 (d, J = 4.1 Hz, 1H), 2.77 (dd, J = 18.0, 3.1 Hz, 1H), 2.66 (dd, J = 18.0, 9.0 Hz, 1H), 2.21 ppm (s, 3H).

^{13}C NMR (125 MHz, $CDCl_3$) δ = 208.6, 167.0, 147.9, 120.6, 66.8, 51.9, 48.7, 30.9 ppm.

IR (film) ν_{max} = 3435, 2928, 1708, 1655, 1274, 1169, 987, 720, 441 cm^{-1}.

HRMS (m/z): (ESI) calcd for $C_8H_{12}NaO_4^+$ $[M + Na]^+$ 195.0628, found 195.0625.

HPLC traces [Chiralpak IB N-5 (0.46 cm × 25 cm), 20% *i*-PrOH/hexanes, v = 1.0 mL·min^{-1}, λ = 210 nm, t (major) = 7.98 min, t (minor) = 9.07 min].

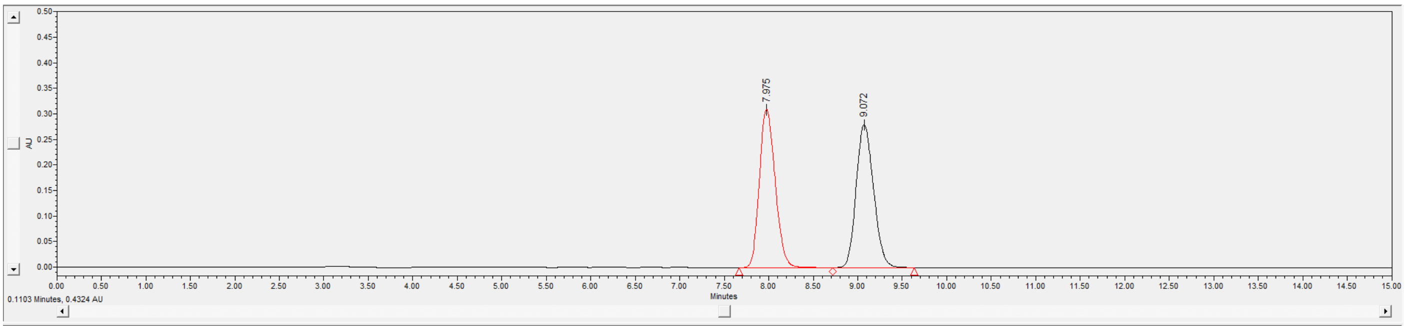

	Name	Retention Time (min)	Area (μV*sec)	% Area	Height (μV)	Int Type	Amount	Units	Peak Type	Peak Codes
1		7.975	3810921	50.07	309120	BV			Unknown	
2		9.072	3800807	49.93	279674	VB			Unknown	

Preparation of β-hydroxyketone 12 with 30% ee (entry 4, Table S1):

To a 50 mL round-bottom flask charged with aldehyde **10** (0.11 g, 1.0 mmol, 1.0 equiv), acetone (2.0 mL) and DMSO (8.0 mL) at 23 °C was added D-proline (23 mg, 0.20 mmol, 0.20 equiv) in one portion. The resulting mixture was stirred for 12 h before Et_2O (5 mL), ice water (10 mL) and $NaHCO_3$ (5 mL, sat. aq.) were sequentially added to the reaction flask. The layers were separated, and the aqueous layer was extracted with

Et_2O (6 × 5 mL). The combined organic layers were washed with brine (10 mL), dried (Na_2SO_4) and concentrated *in vacuo*. Flash column chromatography (silica gel, hexanes: EtOAc 2:1) afforded β-hydroxyketone **12** (55 mg, 32%) in 30% ee as a colorless oil.

HPLC traces [Chiralpak IB N-5 (0.46 cm × 25 cm), 20% *i*-PrOH/hexanes, v = 1.0 mL·min^{-1}, λ = 210 nm, t (major) = 8.04 min, t (minor) = 9.16 min].

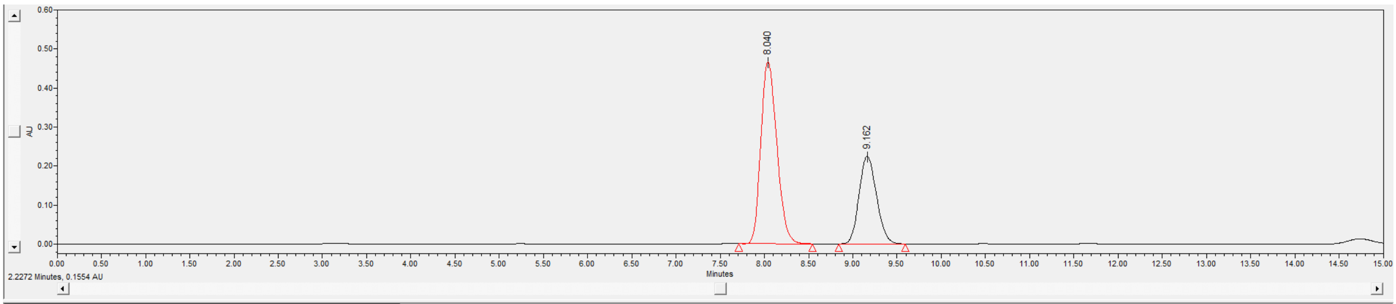

	Name	Retention Time (min)	Area (μV*sec)	% Area	Height (μV)	Int Type	Amount	Units	Peak Type	Peak Codes
1		8.040	5867045	65.55	465678	bb			Unknown	
2		9.162	3083123	34.45	224164	bb			Unknown	

Preparation of β-hydroxyketone 12 with 46% ee (entry 11, Table S1):

To a flame-dried 25 mL round-bottom flask charged with (*S*)-BINOL (57 mg, 0.10 mmol, 0.2 equiv), oven-dried 4 Å molecular sieves (0.5 g) and CH_2Cl_2 (5 mL) at 23 °C was added a solution of Ti(O*i*-Pr)$_4$ (30 μL, 0.10 mmol, 0.1 equiv) in CH_2Cl_2 (0.10 mL). The resulting mixture was stirred for 1 h before it was cooled to −70 °C and 2-methoxypropene (0.19 mL, 2.0 mmol, 2.0 equiv) and a solution of aldehyde **10** (0.11 g, 1.0 mmol, 1.0 equiv) in CH_2Cl_2 (1 mL) were sequentially added to the reaction flask. The reaction was warmed to −30 °C and stirred for 8 h before it was warmed to 0 °C and Et_2O (10 mL) and HCl (2.0 mL, 1 M aq., 2.0 mmol, 2.0 equiv) were sequentially added. The resulting mixture was stirred for 10 min before it was added $NaHCO_3$ (5 mL, sat. aq.) dropwise. The layers were separated, and the aqueous layer was extracted with EtOAc (6 × 5 mL). The combined organic layers were washed with brine (10 mL), dried (Na_2SO_4) and concentrated *in vacuo*. Flash column chromatography (silica gel, hexanes: EtOAc 2:1) afforded β-hydroxyketone **12** (95 mg, 55%) in 46% ee as a colorless oil.

HPLC traces [Chiralpak IB N-5 (0.46 cm × 25 cm), 20% *i*-PrOH/hexanes, v = 1.0 mL·min^{-1}, λ = 210 nm, t (major) = 7.92 min, t (minor) = 8.98 min].

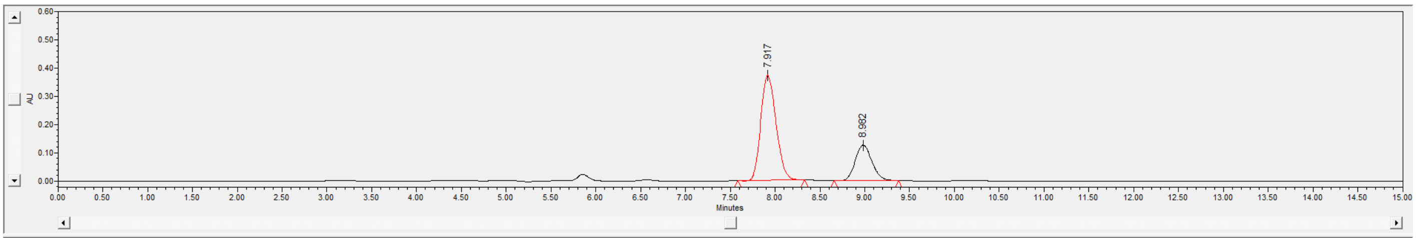

	Name	Retention Time (min)	Area (µV*sec)	% Area	Height (µV)	Int Type	Amount	Units	Peak Type	Peak Codes
1		7.917	4384195	73.02	372336	bb			Unknown	
2		8.982	1619998	26.98	125885	bb			Unknown	

Two-step synthesis of β-hydroxyketone (–)-12 with 99% ee:

Allylic alcohol 11:

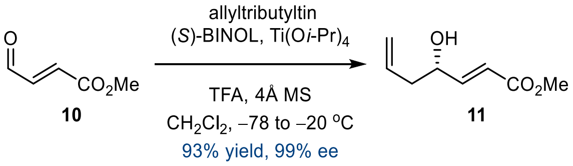

To a flame-dried 50 mL round-bottom flask charged with (*S*)-BINOL (0.27 g, 0.94 mmol, 0.2 equiv), oven-dried 4 Å molecular sieves (1.9 g), and CH_2Cl_2 (20 mL) at 23 °C were sequentially added a solution of Ti(O*i*-Pr)$_4$ (0.14 mL, 0.47 mmol, 0.1 equiv) in CH_2Cl_2 (1 mL) and a solution of TFA (2.5 µL, 33 µmol, 0.007 equiv) in CH_2Cl_2 (0.1 mL). The resulting mixture was warmed to 37 °C and stirred for 1 h before it was cooled to 23 °C and a solution of aldehyde **10** (0.54 g, 4.7 mmol, 1.0 equiv) in CH_2Cl_2 (5 mL) was added dropwise to the reaction flask. The reaction was stirred at 23 °C for an additional 0.5 h before it was cooled to –78 °C and allyltributyltin (2.2 mL, 7.1 mmol, 1.5 equiv) was added dropwise. The resulting mixture was stirred at –78 °C for an additional 10 min before it was warmed to –20 °C and stirred for 12 h. The reaction was warmed to 0 °C and stirred for 10 min before it was quenched with $NaHCO_3$ (20 mL, sat. aq.). The layers were separated, and the aqueous layer was extracted with CH_2Cl_2 (4 × 10 mL). The combined organic layers were dried (Na_2SO_4) and concentrated *in vacuo*. Flash column chromatography (silica gel, CH_2Cl_2:Et_2O 10:1) afforded allylic alcohol **11** (0.68 g, 93%) as a colorless oil.

TLC: R_f = 0.55 (silica gel, CH_2Cl_2:Et_2O 5:1).

^{1}H NMR (600 MHz, $CDCl_3$) δ = 6.96 (dd, *J* = 15.7, 4.5 Hz, 1H), 6.08 (dd, *J* = 15.7, 1.8 Hz, 1H), 5.79 (ddt, *J* = 16.2, 11.0, 7.2 Hz, 1H), 5.21 – 5.20 (m, 1H), 5.19 – 5.16 (m, 1H), 4.39 – 4.35 (m, 1H), 3.74 (s, 3H), 2.46 –

2.40 (m, 1H), 2.34 – 2.28 (m, 1H), 1.91 ppm (d, $J = 4.7$ Hz, 1H).

^{13}C NMR (150 MHz, $CDCl_3$) $\delta = 167.0, 149.5, 133.1, 120.3, 119.6, 69.9, 51.8, 41.2$ ppm.

IR (film) $\nu_{max} = 3431, 1728, 1706, 1438, 1310, 1280, 1173, 985, 925$ cm^{-1}.

HRMS (m/z): (ESI) calcd for $C_8H_{13}O_3^+$ $[M + H]^+$ 157.0859, found 157.0857.

$[\alpha]_D^{25} = -12.7$ (c = 0.10, $CHCl_3$).

***Note:** The enantiomeric excess of **11** was determined later using β-hydroxyketone (–)-**12**.

β-Hydroxyketone (–)-12 and lactol 12':

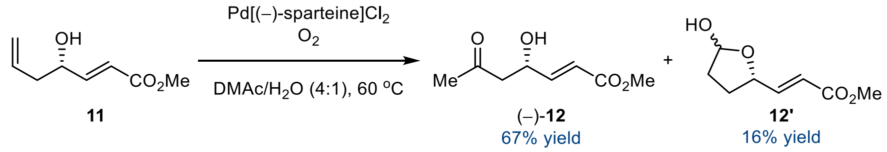

To a 50 mL round-bottom flask charged with allylic alcohol **11** (0.62 g, 4.0 mmol, 1.0 equiv), DMAc (12 mL) and H_2O (3 mL) at 23 °C under O_2 atmosphere (1 atm) was added a solution of Pd[(–)-sparteine]Cl_2[1] (16 mg, 40 μmol, 0.01 equiv) in DMAc/H_2O (4:1, 5 mL). The resulting mixture was warmed to 60 °C and stirred for 20 h before it was cooled to 0 °C and Et_2O (10 mL) and $NaHCO_3$ (10 mL, sat. aq.) were sequentially added to the reaction flask. The layers were separated, and the aqueous layer was extracted with Et_2O (6 × 15 mL). The combined organic layers were washed with brine (10 mL), dried (Na_2SO_4) and concentrated *in vacuo*. Flash column chromatography (silica gel, hexanes: EtOAc 2:1) afforded β-hydroxyketone (–)-**12** (0.46 g, 67%) in 99% ee as a colorless oil, along with lactol **12'** (0.11 g, 16%, dr = 1:0.73) as an inseparable mixture as a colorless oil.

(–)-**12: HPLC traces** [Chiralpak IB N-5 (0.46 cm × 25 cm), 20% *i*-PrOH/hexanes, v = 1.0 mL·min^{-1}, $\lambda = 210$ nm, t (major) = 7.99 min, t (minor) = 9.11 min].

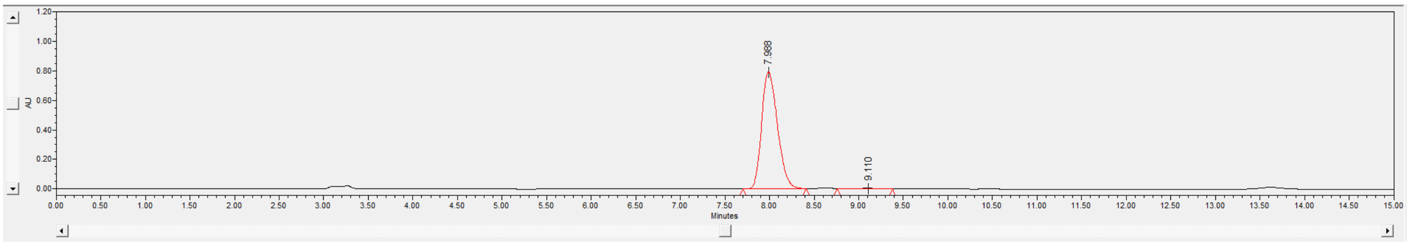

	Name	Retention Time (min)	Area (µV*sec)	% Area	Height (µV)	Int Type	Amount	Units	Peak Type	Peak Codes
1		7.988	9782195	99.51	791805	bb			Unknown	
2		9.110	47867	0.49	3577	bb			Unknown	I08

$[\alpha]_D^{25}$ = –19.5 (c = 0.10, $CHCl_3$).

12’: TLC: R_f = 0.35 (silica gel, hexanes:EtOAc 1:1).

^{1}H NMR (500 MHz, $CDCl_3$) δ = 7.01 (dd, J = 15.6, 5.6 Hz, 0.73H), 6.90 (dd, J = 15.6, 4.7 Hz, 1H), 6.05 (dd, J = 15.6, 1.5 Hz, 0.73H), 6.01 (dd, J = 15.6, 1.7 Hz, 1H), 5.68 – 5.60 (m, 1H), 5.56 (t, J = 3.5 Hz, 0.73H), 4.87 – 4.81 (m, 1H), 4.62 (dddd, J = 7.3, 5.8, 4.7, 1.1 Hz, 0.73H), 3.74 (s, 2.19H), 3.73 (s, 3H), 2.66 – 2.58 (m, 1.73H), 2.33 (dq, J = 12.2, 8.3 Hz, 1H), 2.20 – 2.12 (m, 0.73H), 2.07 – 1.88 (m, 4.19H), 1.70 (dddd, J = 12.4, 9.2, 5.6, 4.6 Hz, 1H).

^{13}C NMR (125 MHz, $CDCl_3$) δ = 167.0 (2C), 149.3, 147.9, 120.4, 120.1, 99.1 (2C), 79.2, 77.0, 51.8 (2C), 33.9, 32.5, 29.6, 29.4 ppm.

IR (film) ν_{max} = 3428, 2955, 2924, 1722, 1660, 1437, 1308, 1276, 1046, 979, 575 cm^{-1}.

HRMS (m/z): (ESI) calcd for $C_8H_{12}NaO_4^+$ $[M + Na]^+$ 195.0628, found 195.0625.

$[\alpha]_D^{25}$ = –5.6 (c = 0.10, $CHCl_3$).

Enynes 13 and 13’:

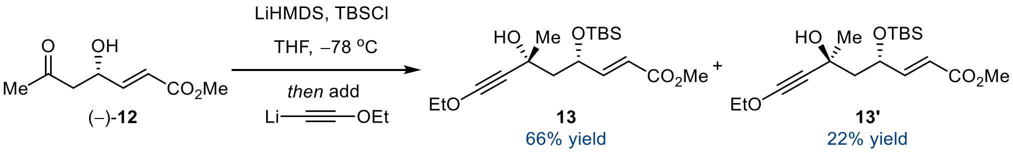

To a flame-dried 100 mL round-bottom flask charged with β-hydroxyketone (–)-**12** (0.45 g, 2.6 mmol, 1.0 equiv) and THF (20 mL) at –78 °C were sequentially added a solution of TBSCl (0.47 g, 3.1 mmol, 1.2 equiv) in THF (3 mL) and a solution of LiHMDS (2.9 mL, 1.0 M in THF, 2.9 mmol, 1.1 equiv). The resulting mixture was stirred for 2 h before freshly prepared lithium ethoxyacetylide[2] (39 mL, 0.2 M in THF, 7.8 mmol, 3.0

equiv) was added dropwise. The reaction was slowly warmed to −10 °C over 1 h before it was quenched with $NaHCO_3$ (25 mL, sat. aq.). The layers were separated, and the aqueous layer was extracted with EtOAc (4 × 15 mL). The combined organic layers were dried (Na_2SO_4) and concentrated *in vacuo*. Flash column chromatography (silica gel, hexanes:EtOAc 3:1) afforded enyne **13** (0.61 g, 66%) as a colorless oil, along with enyne **13'** (0.20 g, 22%) as a colorless oil.

13: **TLC:** R_f = 0.40 (silica gel, hexanes:EtOAc 2:1).

^{1}H NMR (600 MHz, $CDCl_3$) δ = 7.19 (dd, *J* = 15.6, 5.2 Hz, 1H), 5.99 (dd, *J* = 15.7, 1.7 Hz, 1H), 4.72 – 4.66 (m, 1H), 4.06 (q, *J* = 7.1 Hz, 2H), 3.73 (s, 3H), 3.22 (s, 1H), 2.04 – 1.94 (m, 2H), 1.50 (s, 3H), 1.34 (t, *J* = 7.1 Hz, 3H), 0.92 (s, 9H), 0.11 (s, 3H), 0.07 ppm (s, 3H).

^{13}C NMR (150 MHz, $CDCl_3$) δ = 167.1, 151.4, 119.4, 93.5, 74.6, 71.2, 67.2, 51.7, 49.9, 42.3, 32.3, 26.0 (3C), 18.2, 14.6, -4.3, -4.8 ppm.

IR (film) ν_{max} = 3468, 2937, 2262, 1731, 1260, 1100, 837, 779 cm^{-1}.

HRMS (m/z): (ESI) calcd for $C_{18}H_{32}NaO_5Si^+$ $[M + Na]^+$ 379.1911, found 379.1910.

$[\alpha]_D^{25}$ = +4.5 (c = 0.10, $CHCl_3$).

13': **TLC:** R_f = 0.45 (silica gel, hexanes:EtOAc 3:1).

^{1}H NMR (600 MHz, C_6D_6) δ = 7.09 (dd, *J* = 15.7, 6.2 Hz, 1H), 6.03 (dd, *J* = 15.7, 1.3 Hz, 1H), 4.95 – 4.92 (m, 1H), 3.97 (s, 1H), 3.66 (q, *J* = 7.1 Hz, 2H), 3.40 (s, 3H), 1.83 (dd, *J* = 14.1, 9.6 Hz, 1H), 1.56 – 1.53 (m, 4H), 0.97 – 0.93 (m, 12H), 0.21 (s, 3H), 0.04 ppm (s, 3H).

^{13}C NMR (150 MHz, C_6D_6) δ = 166.4, 150.7, 120.4, 93.5, 74.3, 72.4, 67.5, 51.2, 49.8, 42.7, 32.7, 26.0 (3C), 18.2, 14.3, -3.7, -4.5 ppm.

IR (film) ν_{max} = 3506, 2925, 2858, 2262, 1730, 1259, 1098, 842, 781 cm^{-1}.

HRMS (m/z): (ESI) calcd for $C_{18}H_{32}NaO_5Si^+$ $[M + Na]^+$ 379.1911, found 379.1911.

$[\alpha]_D^{25}$ = +6.7 (c = 0.10, $CHCl_3$).

Preparation of enynes 13 and 13' without additives:

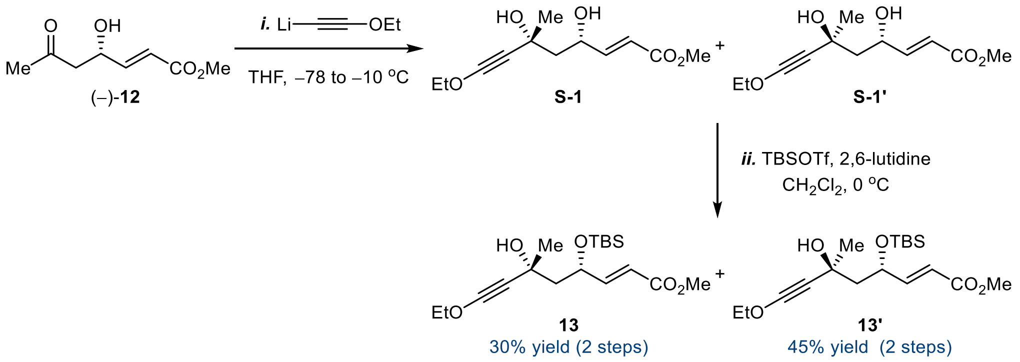

To a flame-dried 50 mL round-bottom flask charged with β-hydroxyketone (–)-**12** (0.10 g, 0.58 mmol, 1.0 equiv) and THF (5 mL) at –78 °C was added freshly prepared lithium ethoxyacetylide[2] (12 mL, 0.2 M in THF, 2.3 mmol, 4.0 equiv) dropwise. The resulting mixture was slowly warmed to –10 °C over 1 h before it was quenched with $NaHCO_3$ (10 mL, sat. aq.). The layers were separated, and the aqueous layer was extracted with EtOAc (6 × 10 mL). The combined organic layers were washed with brine (10 mL), dried (Na_2SO_4) and concentrated *in vacuo* to afford the crude mixture of unstable diols **S-1** and **S-1'** as pale yellow oil, which was used without further purification.

To a flame-dried 25 mL round-bottom flask charged with diols **S-1** and **S-1'** (crude, obtained above) and CH_2Cl_2 (10 mL) at 0 °C were sequentially added 2,6-lutidine (0.20 mL, 1.7 mmol, 3.0 equiv) and TBSOTf (0.28 mL, 1.2 mmol, 2.0 equiv). The resulting mixture was stirred for 1 h before it was quenched with $NaHCO_3$ (10 mL, sat. aq.). The layers were separated, and the aqueous layer was extracted with CH_2Cl_2 (4 × 5 mL). The combined organic layers were dried (Na_2SO_4) and concentrated *in vacuo*. Flash column chromatography (silica gel, hexanes:EtOAc 3:1) afforded enyne **13** (62 mg, 30%) as a colorless oil, along with enyne **13'** (93 mg, 45%) as a colorless oil.

Preparation of enynes 13 and 13' with $TiCl_4$ as additive:

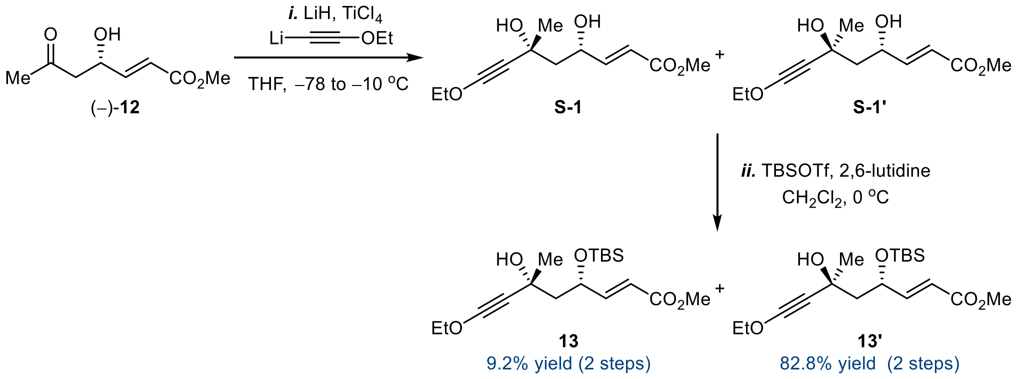

To a flame-dried 50 mL round-bottom flask charged with β-hydroxyketone (−)-**12** (0.10 g, 0.58 mmol, 1.0 equiv) and THF (5 mL) at −30 °C was added LiH (6.9 mg, 0.87 mmol, 1.5 equiv). The resulting mixture was stirred for 0.5 h before a solution of $TiCl_4$ (0.87 mL, 1.0 M in CH_2Cl_2, 0.87 mmol, 1.5 equiv) was added to the flask. The reaction was stirred at −30 °C for an additional 0.5 h before it was cooled to −78 °C and freshly prepared lithium ethoxyacetylide[2] (12 mL, 0.2 M in THF, 2.3 mmol, 4.0 equiv) was added dropwise. The resulting mixture was slowly warmed to −10 °C over 1 h before it was quenched with $NaHCO_3$ (10 mL, sat. aq.). The layers were separated, and the aqueous layer was extracted with EtOAc (6 × 10 mL). The combined organic layers were washed with brine (10 mL), dried (Na_2SO_4) and concentrated *in vacuo* to afford the crude mixture of unstable diols **S-1** and **S-1'** as pale yellow oil, which was used without further purification.

To a flame-dried 25 mL round-bottom flask charged with diols **S-1** and **S-1'** (crude, obtained above) and CH_2Cl_2 (10 mL) at 0 °C were sequentially added 2,6-lutidine (0.20 mL, 1.7 mmol, 3.0 equiv) and TBSOTf (0.28 mL, 1.2 mmol, 2.0 equiv). The resulting mixture was stirred for 1 h before it was quenched with $NaHCO_3$ (10 mL, sat. aq.). The layers were separated, and the aqueous layer was extracted with CH_2Cl_2 (4 × 5 mL). The combined organic layers were dried (Na_2SO_4) and concentrated *in vacuo*. Flash column chromatography (silica gel, hexanes:EtOAc 3:1) afforded enyne **13** (19 mg, 9.2%) as a colorless oil, along with enyne **13'** (0.17 g, 82.8%) as a colorless oil.

Preparation of enynes 13 and 13' with $MgBr_2$ as additive:

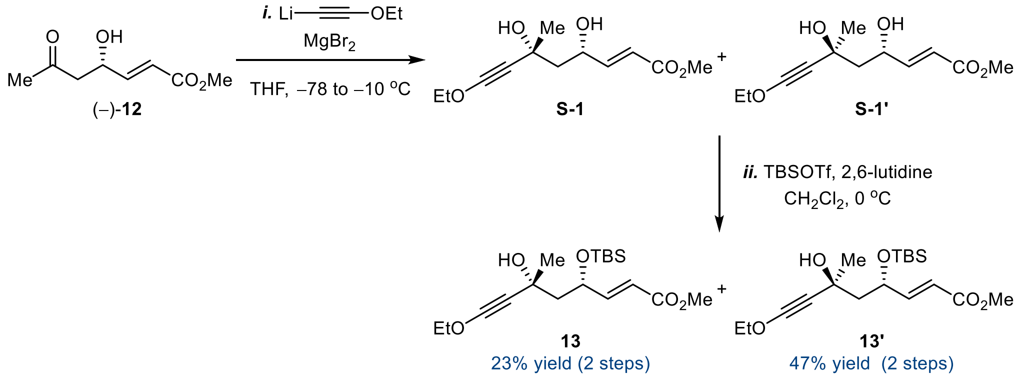

To a flame-dried 50 mL round-bottom flask charged with β-hydroxyketone (−)-**12** (0.10 g, 0.58 mmol, 1.0 equiv), $MgBr_2$ (0.22 g, 1.2 mmol, 2.0 equiv), and THF (10 mL) at −78 °C was added freshly prepared lithium

ethoxyacetylide[2] (12 mL, 0.2 M in THF, 2.3 mmol, 4.0 equiv) dropwise. The resulting mixture was slowly warmed to −10 °C over 1 h before it was quenched with $NaHCO_3$ (10 mL, sat. aq.). The layers were separated, and the aqueous layer was extracted with EtOAc (6 × 10 mL). The combined organic layers were washed with brine (10 mL), dried (Na_2SO_4) and concentrated *in vacuo* to afford the crude mixture of unstable diols **S-1** and **S-1'** as pale yellow oil, which was used without further purification.

To a flame-dried 25 mL round-bottom flask charged with diols **S-1** and **S-1'** (crude, obtained above) and CH_2Cl_2 (10 mL) at 0 °C were sequentially added 2,6-lutidine (0.20 mL, 1.7 mmol, 3.0 equiv) and TBSOTf (0.28 mL, 1.2 mmol, 2.0 equiv). The resulting mixture was stirred for 1 h before it was quenched with $NaHCO_3$ (10 mL, sat. aq.). The layers were separated, and the aqueous layer was extracted with CH_2Cl_2 (4 × 5 mL). The combined organic layers were dried (Na_2SO_4) and concentrated *in vacuo*. Flash column chromatography (silica gel, hexanes:EtOAc 3:1) afforded enyne **13** (48 mg, 23%) as a colorless oil, along with enyne **13'** (97 mg, 47%) as a colorless oil.

Preparation of enynes 13 and 13' with LiBr as additive:

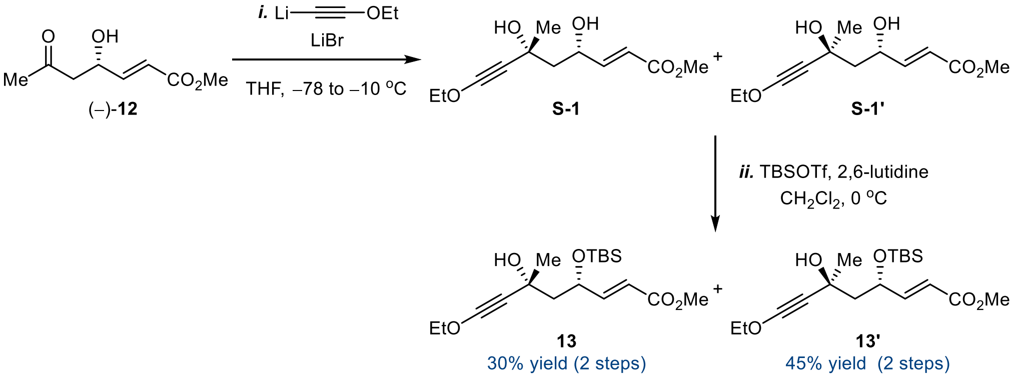

To a flame-dried 50 mL round-bottom flask charged with β-hydroxyketone (−)-**12** (0.10 g, 0.58 mmol, 1.0 equiv), LiBr (0.50 g, 5.8 mmol, 10 equiv), and THF (10 mL) at −78 °C was added freshly prepared lithium ethoxyacetylide[2] (12 mL, 0.2 M in THF, 2.3 mmol, 4.0 equiv) dropwise. The resulting mixture was slowly warmed to −10 °C over 1 h before it was quenched with $NaHCO_3$ (10 mL, sat. aq.). The layers were separated, and the aqueous layer was extracted with EtOAc (6 × 10 mL). The combined organic layers were washed with brine (10 mL), dried (Na_2SO_4) and concentrated *in vacuo* to afford the crude mixture of unstable diols **S-1** and

S-1’ as pale yellow oil, which was used without further purification.

To a flame-dried 25 mL round-bottom flask charged with diols **S-1** and **S-1’** (crude, obtained above) and CH_2Cl_2 (10 mL) at 0 °C were sequentially added 2,6-lutidine (0.20 mL, 1.7 mmol, 3.0 equiv) and TBSOTf (0.28 mL, 1.2 mmol, 2.0 equiv). The resulting mixture was stirred for 1 h before it was quenched with $NaHCO_3$ (10 mL, sat. aq.). The layers were separated, and the aqueous layer was extracted with CH_2Cl_2 (4 × 5 mL). The combined organic layers were dried (Na_2SO_4) and concentrated *in vacuo*. Flash column chromatography (silica gel, hexanes:EtOAc 3:1) afforded enyne **13** (62 mg, 30%) as a colorless oil, along with enyne **13’** (93 mg, 45%) as a colorless oil.

Preparation of enynes 13 and 13’ with $ZnBr_2$ as additive:

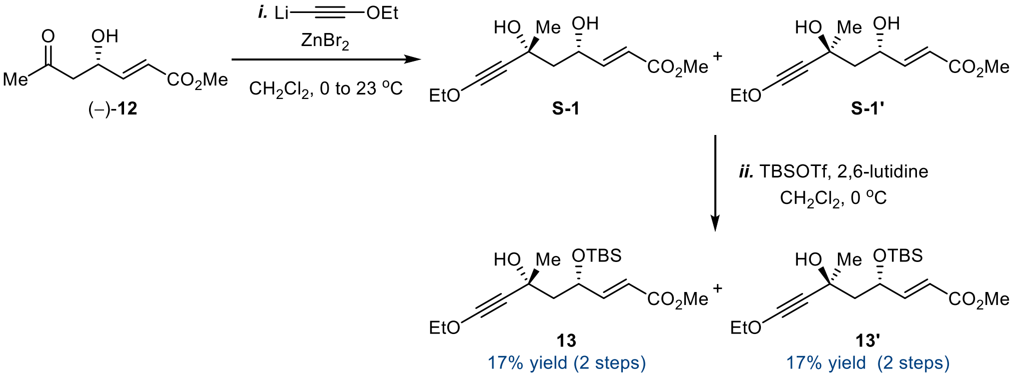

To a flame-dried 50 mL round-bottom flask charged with $ZnBr_2$ (0.27 g, 1.2 mmol, 2.0 equiv) and CH_2Cl_2 (10 mL) at 0 °C was added β-hydroxyketone (−)-**12** (0.10 g, 0.58 mmol, 1.0 equiv) in CH_2Cl_2 (2 mL). The resulting mixture was stirred for 1 h before freshly prepared lithium ethoxyacetylide[2] (12 mL, 0.2 M in THF, 2.3 mmol, 4.0 equiv) was added dropwise to the flask. The resulting mixture was slowly warmed to 23 °C and stirred for 2 h before it was quenched with $NaHCO_3$ (10 mL, sat. aq.). The layers were separated, and the aqueous layer was extracted with CH_2Cl_2 (6 × 10 mL). The combined organic layers were washed with brine (10 mL), dried (Na_2SO_4) and concentrated *in vacuo* to afford the crude mixture of unstable diols **S-1** and **S-1’** as pale yellow oil, which was used without further purification.

To a flame-dried 25 mL round-bottom flask charged with diols **S-1** and **S-1’** (crude, obtained above) and CH_2Cl_2 (10 mL) at 0 °C were sequentially added 2,6-lutidine (0.20 mL, 1.7 mmol, 3.0 equiv) and TBSOTf

(0.28 mL, 1.2 mmol, 2.0 equiv). The resulting mixture was stirred for 1 h before it was quenched with $NaHCO_3$ (10 mL, sat. aq.). The layers were separated, and the aqueous layer was extracted with CH_2Cl_2 (4 × 5 mL). The combined organic layers were dried (Na_2SO_4) and concentrated *in vacuo*. Flash column chromatography (silica gel, hexanes:EtOAc 3:1) afforded enyne **13** (35 mg, 17%) as a colorless oil, along with enyne **13'** (35 mg, 17%) as a colorless oil.

Bicycles 15 and 15':

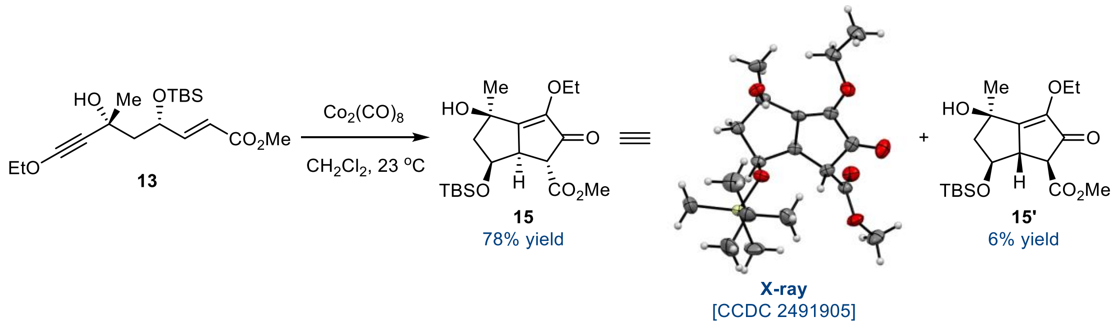

To a flame-dried 100 mL round-bottom flask charged with enyne **13** (0.61 g, 1.7 mmol, 1.0 equiv) and $Co_2(CO)_8$ (0.70 g, 2.0 mmol, 1.2 equiv) at 23 °C was added CH_2Cl_2 (35 mL). The resulting mixture was stirred for 20 min before silica gel (1 g) was added. The reaction was stirred for an additional 2 h before it was filtered through a short pad of celite. The filtrate was collected and concentrated *in vacuo*. Flash column chromatography (silica gel, CH_2Cl_2:Et_2O 20:1) afforded bicycle **15** (0.51 g, 78%) as a white solid, along with bicycle **15'** (39 mg, 6%) as a white amorphous solid.

15: TLC: R_f = 0.40 (silica gel, hexanes:EtOAc 2:1).

^{1}H NMR (600 MHz, C_6D_6) δ = 4.45 (dq, J = 9.6, 7.1 Hz, 1H), 4.10 (dq, J = 9.6, 7.1 Hz, 1H), 3.79 (td, J = 3.4, 0.9 Hz, 1H), 3.62 (d, J = 3.7 Hz, 1H), 3.44 (s, 3H), 3.20 (t, J = 3.6 Hz, 1H), 2.68 (s, 1H), 1.88 (dd, J = 14.3, 0.9 Hz, 1H), 1.59 (dd, J = 14.2, 3.3 Hz, 1H), 1.41 (s, 3H), 1.08 (t, J = 7.1 Hz, 3H), 0.76 (s, 9H), -0.09 (s, 3H), -0.10 ppm (s, 3H).

^{13}C NMR (150 MHz, C_6D_6) δ = 197.6, 169.7, 157.9, 148.1, 75.0, 71.1, 67.0, 53.0, 52.9, 52.0, 50.6, 26.5, 25.8 (3C), 18.1, 15.4, -4.9, -5.2 ppm.

IR (film) ν_{max} = 2932, 2855, 1750, 1720, 1368, 1260, 1127, 1045, 840, 781 cm^{-1}.

HRMS (m/z): (ESI) calcd for $C_{19}H_{32}NaO_6Si^+$ $[M + Na]^+$ 407.1860, found 407.1859.

$[\alpha]_D^{25}$ = +73.1 (c = 0.10, $CHCl_3$).

m.p. = 151–152 °C.

15': TLC: R_f = 0.15 (silica gel, hexanes:EtOAc 2:1).

^{1}H NMR (600 MHz, C_6D_6) δ = 4.26 (dq, *J* = 9.9, 7.1 Hz, 1H), 4.08 (dq, *J* = 9.9, 7.1 Hz, 1H), 3.58 (dd, *J* = 8.7, 3.3 Hz, 1H), 3.44 – 3.38 (m, 4H), 3.01 (d, *J* = 3.3 Hz, 1H), 2.10 – 1.96 (m, 2H), 1.37 (s, 3H), 1.05 (t, *J* = 7.1 Hz, 3H), 0.92 (s, 9H), 0.01 (s, 3H), -0.01 ppm (s, 3H).

^{13}C NMR (150 MHz, C_6D_6) δ = 198.1, 169.3, 156.7, 147.7, 75.3, 72.6, 67.0, 57.2, 53.7, 52.0, 51.5, 26.6, 25.8 (3C), 18.1, 15.7, -4.5, -4.9 ppm.

IR (film) ν_{max} = 3462, 2922, 2856, 1714, 1254, 1094, 840, 774 cm^{-1}.

HRMS (m/z): (ESI) calcd for $C_{19}H_{32}NaO_6Si^+$ $[M + Na]^+$ 407.1860, found 407.1860.

$[\alpha]_D^{25}$ = –33.8 (c = 0.10, $CHCl_3$).

Preparation of bicycles 15 and 15' from unstable diol S-1:

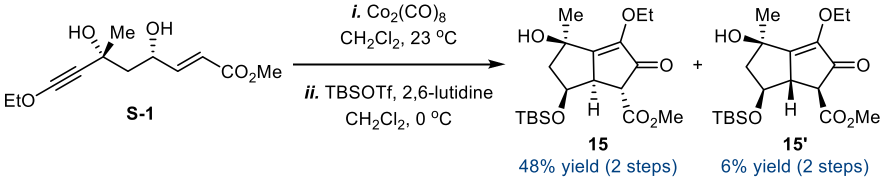

To a flame-dried 10 mL reaction tube charged with diol **S-1** (50 mg, 0.21 mmol, 1.0 equiv) and $Co_2(CO)_8$ (86 mg, 0.25 mmol, 1.2 equiv) at 23 °C was added CH_2Cl_2 (5 mL). The resulting mixture was stirred for 20 min before silica gel (0.1 g) was added. The reaction was stirred for an additional 1 h before it was filtered through a short pad of celite. The filtrate was collected and concentrated *in vacuo* to afford the crude mixture of bicyclic diols as an amorphous solid, which were used directly without further purification.

To a flame-dried 25 mL round-bottom flask charged with the bicyclic diols (crude, obtained above) and CH_2Cl_2 (5 mL) at 0 °C were sequentially added 2,6-lutidine (73 µL, 0.63 mmol, 3.0 equiv) and TBSOTf (96 µL, 0.42 mmol, 2.0 equiv). The resulting mixture was stirred for 0.5 h before it was quenched with $NaHCO_3$

(5 mL, sat. aq.). The layers were separated, and the aqueous layer was extracted with CH_2Cl_2 (4 × 5 mL). The combined organic layers were dried (Na_2SO_4) and concentrated *in vacuo*. Flash column chromatography (silica gel, CH_2Cl_2:Et_2O 20:1) afforded enyne **15** (39 mg, 48%) as a colorless oil, along with enyne **15'** (4.8 mg, 6%) as a colorless oil.

***Note:** The relatively pure starting material diol **S-1** was obtained by quick elution of the crude 1,2-addition mixture through a short silica gel column using cold eluent (0 °C, hexanes:EtOAc 1:1) due to its instability on silica gel.

Bicyclic enol 8:

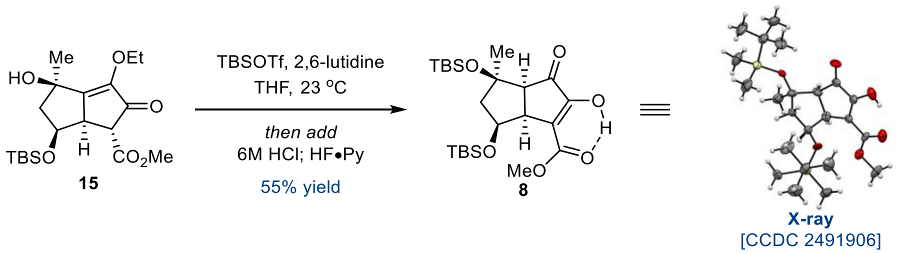

To a flame-dried 100 mL round-bottom flask charged with bicycle **15** (0.50 g, 1.3 mmol, 1.0 equiv) and THF (6 mL) at 23 °C were sequentially added 2,6-lutidine (3.0 mL, 26 mmol, 20 equiv) and TBSOTf (3.0 mL, 13 mmol, 10 equiv). The resulting mixture was stirred for 1 h before it was cooled to 0 °C, and HCl (5.5 mL, 6.0 M aq., 33 mmol, 25 equiv) was added dropwise to the flask. The reaction was stirred at 0 °C for an additional 1 h before HF·Py (1.0 mL) was added. The resulting mixture was stirred at 0 °C for an additional 2 h before it was cooled to −10 °C and Et_2O (20 mL) and $NaHCO_3$ (35 mL, sat. aq.) were sequentially added. The layers were separated, and the aqueous layer was extracted with EtOAc (5 × 20 mL). The combined organic layers were dried (Na_2SO_4) and concentrated *in vacuo*. Flash column chromatography (silica gel, hexanes:EtOAc 3:1) afforded bicyclic enol **8** (0.34 g, 55%) as a pale yellow solid.

***Note:** HF·Py is very toxic and corrosive. Caution should be exercised when using this complex.

TLC: R_f = 0.55 (silica gel, hexanes:EtOAc 5:1).

^{1}H NMR (600 MHz, $CDCl_3$) δ = 8.66 (s, 1H), 4.30 (ddd, J = 9.9, 8.0, 6.4 Hz, 1H), 3.83 (s, 3H), 3.57 (dd, J =

8.0, 6.1 Hz, 1H), 2.57 (d, J = 6.1 Hz, 1H), 1.89–1.81 (m, 2 H), 1.38 (s, 3H), 0.87 (s, 9H), 0.86 (s, 9H), 0.11 (s, 3H), 0.07 (s, 3H), 0.04 ppm (s, 6H).

^{13}C NMR (125 MHz, $CDCl_3$) δ = 198.5, 168.7, 160.2, 124.6, 76.7, 71.7, 57.9, 52.2, 47.7, 42.4, 31.5, 26.0 (3C), 25.9 (3C), 18.4, 18.2, -2.0, -2.1, -4.7, -4.9 ppm.

IR (film) ν_{max} = 2963, 2924, 2856, 2363, 1734, 1258, 1229, 1098, 837, 773, 750 cm^{-1}.

HRMS (m/z): (ESI) calcd for $C_{23}H_{43}O_6Si_2^+$ $[M + H]^+$ 471.2593, found 471.2591.

$[\alpha]_D^{25}$ = –25.6 (c = 0.10, $CHCl_3$).

m.p. = 166–167 °C.

Table S2. Optimization of the Photochemical *trans*-[2+2] Cycloaddition[a]

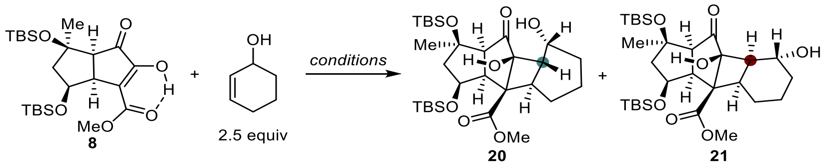

Entry	Conditions	Yield [%][b]	
		**20**	**21**
1[c]	Ir[dF(CF_3)ppy]$_2$(dtbbpy)PF_6 (2 mol%), Sc(OTf)$_3$ (10 mol%), terpy (15 mol%), imidazole (25 mol%), 427 nm, CH_2Cl_2, 0.05 M, 23 °C, 15 h	0	0
2[c]	Ir[dF(CF_3)ppy]$_2$(dtbbpy)PF_6 (2 mol%), *n*-Bu$_2$PO$_4$NMe(*n*-Bu)$_3$ (2.5 mol%), 455 nm, EtOH, 0.1 M, 23 °C, 24 h	0	0
3[c]	$ZrCl_4$ (10 mol%), 440 nm, CH_2Cl_2, 0.1 M, 23 °C, 14 h	0	0
4[c]	4CzIPN (0.4 mol%), *n*-Bu$_2$PO$_4$NMe(*n*-Bu)$_3$ (2.5 mol%), 455 nm CH$_3$CN, 0.1 M, 23 °C, 24 h	0	0
5[d]	Hg (450 W), benzene, 0.02 M or 0.1 M, 40 °C, 5 h	<10	<10
6[c]	390 nm, benzene, 0.02 M, 45 °C, 24 h	0	0
7	350 nm, benzene, 0.02 M, 45 °C, 16 h	25	25
8[e]	350 nm, CH_2Cl_2, 0.02 M, 0 °C, 15 h	<5	<5
9	300 nm, CH_2Cl_2, 0.02 M, –78 °C, 18 h	43	29
10[e]	254 nm, CH_2Cl_2, 0.02 M, –78 °C, 24 h	<5	<5
11[d]	300 nm, Et$_2$O, 0.02 M, –78 °C, 24 h	<5	<5
12[d]	300 nm, EtOAc, 0.02 M, –78 °C, 16 h	<2	<2
13[d]	300 nm, acetone, 0.02 M, –78 °C, 18 h	<5	<5
14	300 nm, MeCN, 0.02 M, –20 °C, 9 h (20 h)[f]	67 (56)[f]	0
15[c]	300 nm, MeOH, 0.02 M, –78 °C, 24 h	0	0
16	**21** instead of **8**, 300 nm, MeCN, 0.02 M, –20 °C, 24 h	0	89

[a]Reactions were performed on 0.02 mmol scale, unless otherwise noted. [b]Isolated yields. [c]No reaction was observed. [d]Unidentified mixture was observed along with the formation of **20** and **21**. [e]Partial recovery of **8** along with formation of **20** and **21**. [f]Isolated yield on 0.50 mmol scale.

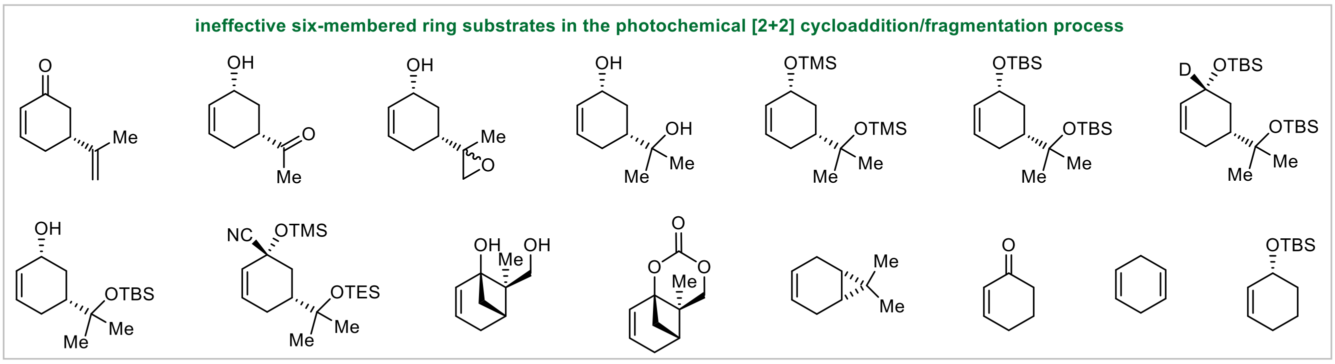

Tetracycles 20 and 21 (entry 9, Table S2):

To a 5 mL quartz glass tube charged with bicyclic enol **8** (9.4 mg, 20 μmol, 1.0 equiv) and 2-cyclohexen-1-ol (5.0 μL, 50 μmol, 2.5 equiv) at 23 °C was added CH_2Cl_2 (1.5 mL). The resulting mixture was degassed for 0.5 h with argon before it was irradiated at 300 nm using a Rayonet® Chamber Reactor model RPR-200 at −78 °C. The reaction was stirred for 18 h before it was removed from the photoreactor and concentrated *in vacuo*. Flash column chromatography (silica gel, cold hexanes:EtOAc 6:1) afforded tetracycle **20** (4.9 mg, 43%) as a colorless oil, along with tetracycle **21** (3.3 mg, 29%) as a colorless oil.

***Note:** Tetracycles **20** and **21** were found to not be very stable during purification using silica gel at room temperature. A one-pot [2+2] cycloaddition/HCl-promoted fragmentation was generally adopted to avoid slow decomposition (*vide infra*).

20: TLC: R_f = 0.55 (silica gel, hexanes:EtOAc 5:1).

^{1}H NMR (600 MHz, C_6D_6) δ = 4.25 (td, *J* = 8.3, 6.5 Hz, 1H), 4.20 (s, 1H), 4.18 (br s, 1H), 3.48 (s, 3H), 2.85 (t, *J* = 7.9 Hz, 1H), 2.53 (d, *J* = 7.7 Hz, 1H), 2.26 (d, *J* = 13.2 Hz, 1H), 2.19 (dd, *J* = 12.3, 8.5 Hz, 1H), 2.08 (t, *J* = 2.1 Hz, 1H), 1.98 (ddd, *J* = 12.3, 6.4, 1.2 Hz, 1H), 1.90 (ddd, *J* = 13.2, 11.6, 3.3 Hz, 1H), 1.78 – 1.70 (m, 2H), 1.64 – 1.60 (m, 1H), 1.40 – 1.37 (m, 1H), 1.12 (s, 9H), 1.08 (s, 3H), 1.07 – 1.02 (m, 2H), 0.93 (s, 9H), 0.25 (s, 3H), 0.24 (s, 3H), 0.04 (s, 3H), 0.03 ppm (s, 3H).

^{13}C NMR (150 MHz, C_6D_6) δ = 214.9, 173.5, 81.3, 79.3, 73.3, 65.5, 63.3, 60.4, 53.7, 51.6, 50.8, 49.4, 35.1, 33.1, 31.8, 29.7, 26.4 (6C), 21.7, 18.7, 18.6, -1.8, -1.9, -3.7, -4.3 ppm.

IR (film) ν_{max} = 3493, 2930, 2855, 1740, 1468, 1259, 1080, 834, 773 cm^{-1}.

HRMS (m/z): (ESI) calcd for $C_{29}H_{53}O_7Si_2^+$ $[M + H]^+$ 569. 3324, found 569. 3324.

$[\alpha]_D^{25}$ = −112.0 (c = 0.02, $CHCl_3$).

21: TLC: R_f = 0.45 (silica gel, hexanes:EtOAc 5:1).

^{1}H NMR (600 MHz, C_6D_6) δ = 4.55 (td, *J* = 10.5, 5.5 Hz, 1H), 4.16 (s, 1H), 4.15 – 4.11 (m, 1H), 3.38 (s, 3H), 2.52 – 2.45 (m, 2H), 2.29 (br s, 1H), 2.06 (dd, *J* = 13.7, 4.3 Hz, 1H), 2.02 – 1.95 (m, 2H), 1.90 (dd, *J* = 13.6,

6.4 Hz, 1H), 1.86 (td, J = 8.5, 6.2 Hz, 1H), 1.40 – 1.33 (m, 2H), 1.29 – 1.23 (m, 2H), 1.18 (s, 9H), 1.14 (s, 3H), 1.15 – 1.08 (m, 1H), 0.93 (s, 9H), 0.31 (s, 3H), 0.29 (s, 3H), 0.00 (s, 3H), -0.01 ppm (s, 3H).

^{13}C NMR (150 MHz, C_6D_6) δ = 210.7, 173.2, 79.8, 78.8, 73.4, 65.2, 57.6, 56.2, 53.0, 52.8, 50.8, 46.9, 37.9, 33.0, 28.9, 26.5 (3C), 26.3 (3C), 20.7, 20.4, 18.8, 18.4, -1.8, -1.9, -4.0 ppm (2C).

IR (film) ν_{max} = 3460, 2927, 2856, 1749, 1716, 1462, 1257, 1062, 1001, 837, 775 cm^{-1}.

HRMS (m/z): (ESI) calcd for $C_{29}H_{52}NaO_7Si_2^+$ $[M + Na]^+$ 591. 3144, found 591. 3147.

$[\alpha]_D^{25}$ = −50.9 (c = 0.02, $CHCl_3$).

Tetracycle 20 (entry 14, Table S2):

0.02 mmol scale: To a 5 mL quartz glass tube charged with bicyclic enol **8** (9.4 mg, 20 µmol, 1.0 equiv) and 2-cyclohexen-1-ol (5.0 µL, 50 µmol, 2.5 equiv) at 23 °C was added MeCN (1.0 mL). The resulting mixture was degassed for 0.5 h with argon before it was irradiated at 300 nm using Rayonet® Chamber Reactor model RPR-200 at −20 °C. The reaction was stirred for 9 h before it was removed from the photoreactor and concentrated *in vacuo*. Flash column chromatography (silica gel, hexanes:EtOAc 6:1) afforded tetracycle **20** (7.6 mg, 67%) as a colorless oil.

0.50 mmol scale: To a 50 mL quartz glass tube charged with bicyclic enol **8** (0.24 g, 0.50 mmol, 1.0 equiv) and 2-cyclohexen-1-ol (0.13 mL, 1.3 mmol, 2.5 equiv) at 23 °C was added MeCN (25 mL). The resulting mixture was degassed for 1 h with argon before it was irradiated at 300 nm using Rayonet® Chamber Reactor model RPR-200 at −20 °C. The reaction was stirred for 20 h before it was removed from the photoreactor and concentrated *in vacuo*. Flash column chromatography (silica gel, hexanes:EtOAc 6:1) afforded tetracycle **20** (0.16 g, 56%) as a colorless oil.

Pentacycle 16:

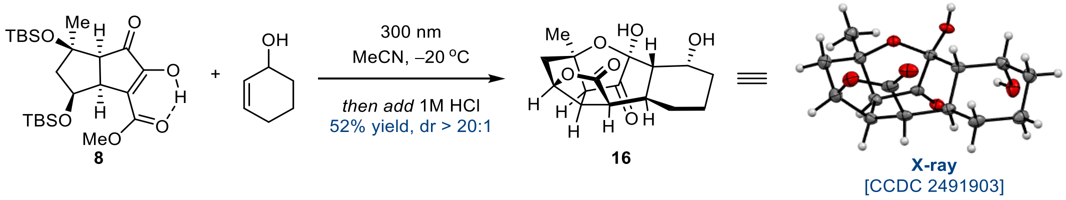

To a 50 mL quartz glass tube charged with bicyclic enol **8** (0.24 g, 0.50 mmol, 1.0 equiv) and 2-cyclohexen-1-ol (0.13 mL, 1.3 mmol, 2.5 equiv) at 23 °C was added MeCN (25 mL). The resulting mixture was degassed for 1 h with argon before it was irradiated at 300 nm using Rayonet® Chamber Reactor model RPR-200 at –20 °C. The reaction was stirred for 20 h before it was removed from the photoreactor and warmed to 23 °C. To the resulting mixture was added HCl (1.5 mL, 1.0 M aq., 1.5 mmol, 3.0 equiv), and the reaction was stirred for an additional 0.5 h before it was cooled to 0 °C and Et_2O (5 mL) and $NaHCO_3$ (10 mL, sat. aq.) were sequentially added. The layers were separated, and the aqueous layer was extracted with EtOAc (5 × 5 mL). The combined organic layers were dried (Na_2SO_4) and concentrated *in vacuo*. Flash column chromatography (silica gel, hexanes:acetone 2:1) afforded pentacycle **16** (80 mg, 52%) as a white solid.

TLC: R_f = 0.45 (silica gel, hexanes:acetone 1.5:1).

^{1}H NMR (500 MHz, $CDCl_3$) δ = 5.11 (t, *J* = 7.4 Hz, 1H), 4.46 – 4.32 (m, 1H), 3.50 (ddd, *J* = 12.2, 9.2, 7.3 Hz, 1H), 2.98 (dd, *J* = 12.4, 2.3 Hz, 1H), 2.77 (d, *J* = 9.2 Hz, 1H), 2.53 (d, *J* = 15.7 Hz, 1H), 2.44 – 2.34 (m, 1H), 2.13 (dt, *J* = 12.1, 3.4 Hz, 1H), 2.07 (dd, *J* = 15.7, 7.3 Hz, 1H), 1.98 (dd, *J* = 12.1, 2.4 Hz, 1H), 1.90 (dt, *J* = 13.9, 2.6 Hz, 1H), 1.74 – 1.62 (m, 2H), 1.57 – 1.50 (m, 2H), 1.48 – 1.40 (m, 1H), 1.44 ppm (s, 3H).

^{13}C NMR (125 MHz, $CDCl_3$) δ = 211.7, 176.4, 103.2, 91.7, 82.5, 66.5, 63.3, 47.6, 45.9, 45.4, 44.8, 33.2, 31.8, 31.6, 22.7, 19.7 ppm.

IR (film) ν_{max} = 3398, 2928, 2362, 1756, 1213, 1037, 748 cm^{-1}.

HRMS (m/z): (ESI) calcd for $C_{16}H_{20}NaO_6^+$ $[M + Na]^+$ 331. 1152, found 331. 1154.

$[\alpha]_D^{25}$ = –12.8 (c = 0.05, $CHCl_3$).

m.p. = 185–186 °C.

Chloride 17:

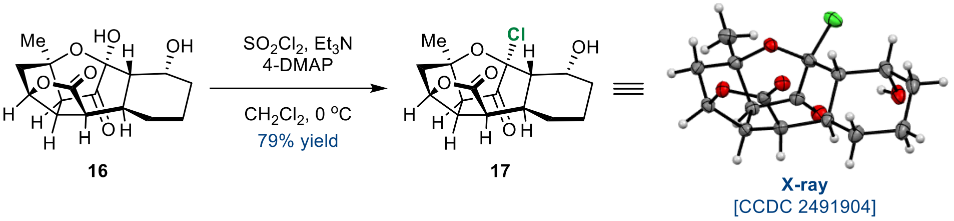

To a flame-dried 25 mL round-bottom flask charged with pentacycle **16** (71 mg, 0.23 mmol, 1.0 equiv), 4-DMAP (28 mg, 0.23 mmol, 1.0 equiv), and CH_2Cl_2 (7 mL) at 0 °C were sequentially added Et_3N (0.13 mL, 0.92 mmol, 4.0 equiv) and SO_2Cl_2 (37 μL, 0.46 mmol, 2.0 equiv). The resulting mixture was stirred for 0.5 h before it was quenched with $NaHCO_3$ (7 mL, sat. aq.) dropwise. The layers were separated, and the aqueous layer was extracted with CH_2Cl_2 (3 × 5 mL). The combined organic layers were dried (Na_2SO_4) and concentrated *in vacuo*. Flash column chromatography (silica gel, CH_2Cl_2:acetone 15:1) afforded chloride **17** (59 mg, 79%) as a white solid.

TLC: R_f = 0.55 (silica gel, CH_2Cl_2:acetone 6:1).

^{1}H NMR (600 MHz, $CDCl_3$) δ = 5.12 (t, *J* = 7.5 Hz, 1H), 4.60 (dt, *J* = 4.0, 2.1 Hz, 1H), 3.52 (ddd, *J* = 12.2, 9.1, 7.6 Hz, 1H), 2.99 (dd, *J* = 12.2, 2.3 Hz, 1H), 2.88 (d, *J* = 9.0 Hz, 1H), 2.61 (d, *J* = 16.0 Hz, 1H), 2.52 – 2.44 (m, 1H), 2.23 – 2.18 (m, 1H), 2.17 (dd, *J* = 11.6, 1.8 Hz, 1H), 2.13 (dd, *J* = 16.0, 7.5 Hz, 1H), 1.97 – 1.92 (m, 1H), 1.78 – 1.67 (m, 1H), 1.57 (s, 3H), 1.54 – 1.44 ppm (m, 3H).

^{13}C NMR (150 MHz, $CDCl_3$) δ = 207.3, 175.5, 105.6, 94.7, 81.9, 67.8, 62.9, 47.4, 47.0, 46.3, 44.7, 33.6, 31.9 (2C), 21.1, 19.4 ppm.

IR (film) ν_{max} = 3554, 2925, 2856, 1760, 1456, 1325, 1213, 1177, 1031, 752, 656 cm^{-1}.

HRMS (m/z): (ESI) calcd for $C_{16}H_{20}ClO_5^+$ $[M + H]^+$ 327. 0994, found 327. 0995.

$[\alpha]_D^{25}$ = +19.5 (c = 0.02, $CHCl_3$).

m.p. = 197–198 °C.

β-Keto tetrahydrofuran 18:

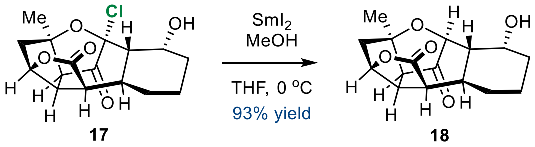

To a flame-dried 25 mL round-bottom flask charged with chloride **17** (49 mg, 0.15 mmol, 1.0 equiv) and MeOH (0.2 mL) at 23 °C was added THF (2 mL). The resulting mixture was degassed for 0.5 h with argon before it was cooled to 0 °C and SmI_2 (4.5 mL, 0.1 M in THF, 0.45 mmol, 3.0 equiv) was added dropwise. The reaction was stirred at 0 °C for an additional 5 min before it was quenched with $NaHCO_3$ (5 mL, sat. aq.) dropwise. The layers were separated, and the aqueous layer was extracted with EtOAc (5 × 5 mL). The combined organic layers were dried (Na_2SO_4) and concentrated *in vacuo*. Flash column chromatography (silica gel, CH_2Cl_2:acetone 15:1) afforded β-keto tetrahydrofuran **18** (41 mg, 93%) as a white amorphous solid.

TLC: R_f = 0.50 (silica gel, CH_2Cl_2:acetone 6:1).

^{1}H NMR (600 MHz, $CDCl_3$) δ = 5.11 (t, *J* = 7.4 Hz, 1H), 4.30 – 4.17 (m, 2H), 3.46 (ddd, *J* = 12.0, 9.2, 7.5 Hz, 1H), 2.97 (dd, *J* = 12.1, 2.6 Hz, 1H), 2.67 (d, *J* = 9.2 Hz, 1H), 2.52 (d, *J* = 15.6 Hz, 1H), 2.47 – 2.39 (m, 1H), 2.19 – 2.09 (m, 2H), 1.93 (dt, *J* = 12.2, 1.7 Hz, 1H), 1.88 – 1.83 (m, 1H), 1.75 – 1.67 (m, 1H), 1.54 – 1.44 (m, 4H), 1.29 ppm (s, 3H).

^{13}C NMR (150 MHz, $CDCl_3$) δ = 216.8, 176.2, 91.5, 84.4, 82.4, 69.7, 62.7, 47.2, 45.7, 44.4, 41.3, 32.6, 32.2, 31.1, 20.3, 19.5 ppm.

IR (film) ν_{max} = 2927, 2853, 2362, 2341, 1752, 1260, 1171, 1025, 763, 752 cm^{-1}.

HRMS (m/z): (ESI) calcd for $C_{16}H_{21}O_5^+$ $[M + H]^+$ 293. 1384, found 293. 1387.

$[\alpha]_D^{25}$ = –5.5 (c = 0.02, $CHCl_3$).

Enone 19:

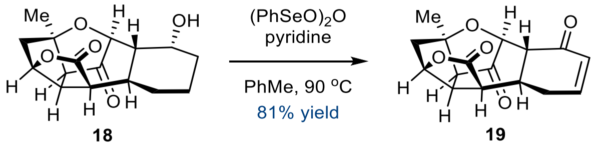

To a flame-dried 10 mL reaction tube charged with β-keto tetrahydrofuran **18** (35 mg, 0.12 mmol, 1.0 equiv), $(PhSeO)_2O$ (0.11 g, 0.30 mmol, 2.5 equiv), and PhMe (5 mL) at 23 °C was added pyridine (24 μL, 0.30 mmol, 2.5 equiv). The resulting mixture was warmed to 90 °C and stirred for 18 h before it was cooled to 23 °C and

concentrated *in vacuo*. Flash column chromatography (silica gel, CH_2Cl_2:acetone 20:1) afforded enone **19** (28 mg, 81%) as a white amorphous solid.

TLC: R_f = 0.55 (silica gel, CH_2Cl_2:acetone 6:1).

^{1}H NMR (600 MHz, $CDCl_3$) δ = 6.97 (ddd, *J* = 10.1, 6.1, 2.2 Hz, 1H), 6.06 (ddd, *J* = 10.1, 3.1, 1.1 Hz, 1H), 5.19 (d, *J* = 1.7 Hz, 1H), 5.15 (t, *J* = 7.3 Hz, 1H), 3.70 – 3.62 (m, 1H), 3.41 (ddd, *J* = 12.2, 9.3, 7.7 Hz, 1H), 3.08 (dd, *J* = 12.1, 2.5 Hz, 1H), 2.82 (dd, *J* = 13.5, 1.7 Hz, 1H), 2.64 (d, *J* = 9.3 Hz, 1H), 2.57 – 2.49 (m, 2H), 2.25 (dt, *J* = 18.9, 5.2 Hz, 1H), 2.13 (dd, *J* = 15.6, 7.2 Hz, 1H), 1.32 ppm (s, 3H).

^{13}C NMR (150 MHz, $CDCl_3$) δ = 212.7, 194.9, 175.9, 150.0, 129.0, 91.3, 83.0, 78.5, 62.4, 46.2, 45.8, 45.4, 42.8, 35.0, 31.1, 20.1 ppm.

IR (film) ν_{max} = 2924, 2851, 2360, 1756, 1673, 1170, 1041, 750 cm^{-1}.

HRMS (m/z): (ESI) calcd for $C_{16}H_{17}O_5^+$ $[M + H]^+$ 289. 1071, found 289. 1071.

$[\alpha]_D^{25}$ = +66.0 (c = 0.02, $CHCl_3$).

(+)-Ineleganolide (4):

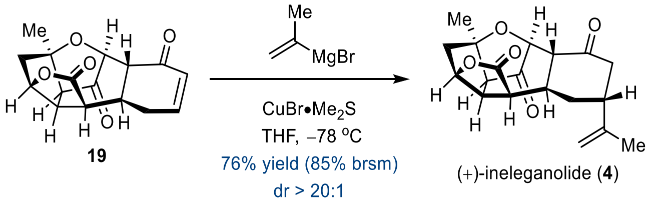

To a flame-dried 25 mL round-bottom flask charged with CuBr·Me_2S (72 mg, 0.35 mmol, 5.0 equiv) and THF (3 mL) at −78 °C was added a solution of *iso*propenylmagnesium bromide (1.4 mL, 0.5 M in THF, 0.70 mmol, 10 equiv) dropwise. The resulting mixture was stirred for 0.5 h before a solution of enone **19** (20 mg, 69 μmol, 1.0 equiv) in THF (1.5 mL) was added dropwise to the flask. The reaction was stirred for an additional 5 h before it was quenched with $NaHCO_3$ (5 mL, sat. aq.) dropwise. The layers were separated, and the aqueous layer was extracted with EtOAc (8 × 5 mL). The combined organic layers were dried (Na_2SO_4) and

concentrated *in vacuo*. Flash column chromatography (silica gel, hexanes:acetone 3:1) afforded (+)-ineleganolide (**4**) (17 mg, 76%) as a white solid, along with recovery of enone **19** (2.5 mg).

TLC: R_f = 0.35 (silica gel, hexanes:acetone 3:1).

^{1}H NMR (600 MHz, $CDCl_3$) δ = 5.12 (t, *J* = 7.4 Hz, 1H), 5.07 (s, 1H), 4.94 (s, 1H), 4.62 (s, 1H), 3.42 (ddd, *J* = 12.1, 9.4, 7.6 Hz, 1H), 3.05 – 2.97 (m, 1H), 3.02 (dd, *J* = 12.1, 2.4 Hz, 1H), 2.79 (br s, 1H), 2.70 (dt, *J* = 12.4, 1.1 Hz, 1H), 2.67 (dt, *J* = 15.6, 2.4 Hz, 1H), 2.59 (d, *J* = 9.3 Hz, 1H), 2.58 (ddd, *J* = 15.2, 6.6, 0.8 Hz, 1H), 2.52 (d, *J* = 15.6 Hz, 1H), 2.26 (tt, *J* = 12.3, 2.8 Hz, 1H), 2.10 (dd, *J* = 15.5, 7.2 Hz, 1H), 1.78 (dq, *J* = 14.0, 3.1 Hz, 1H), 1.71 (s, 3H), 1.29 ppm (s, 3H).

^{13}C NMR (150 MHz, $CDCl_3$) δ = 212.2, 206.4, 176.1, 146.0, 113.9, 91.1, 83.1, 77.5, 62.5, 49.9, 47.1, 45.5, 44.4, 43.8, 40.4, 33.3, 32.7, 22.7, 20.2 ppm.

IR (film) ν_{max} = 2965, 2933, 1758, 1712, 1418, 1365, 1310, 1211, 1190, 1056, 877, 750 cm^{-1}.

HRMS (m/z): (ESI) calcd for $C_{19}H_{23}O_5^+$ $[M + H]^+$ 331.1540, found 331.1540.

$[\alpha]_D^{25}$ = +27.1 (c = 0.02, $CHCl_3$) [Lit.[3] $[\alpha]_D^{25}$ = +26.4 (c = 0.05, $CHCl_3$)].

m.p. = 188–189 °C [Lit.[3] m.p. = 190–192 °C].

III) Natural Product Spectral Comparisons

Table S3. ^1H NMR ($CDCl_3$, 25 °C) Comparison of Natural and Synthetic (+)-Ineleganolide (**4**).

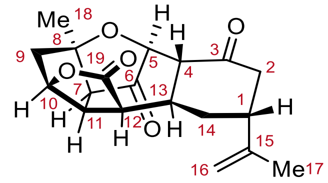

(+)-ineleganolide (**4**)

No.	Isolation[3] δ ^1H [ppm, mult, *J* (Hz)]	Synthetic (Wood)[4] δ ^1H [ppm, mult, *J* (Hz)]	Synthetic (Stoltz)[5] δ ^1H [ppm, mult, *J* (Hz)]	Synthetic (Fürstner)[6] δ ^1H [ppm, mult, *J* (Hz)]	Synthetic (Sarlah)[7] δ ^1H [ppm, mult, *J* (Hz)]	Synthetic (this work) δ ^1H [ppm, mult, *J* (Hz)]
1	2.78 (br s)	2.79 (br s)	2.78 (br s)	2.78 (m)	2.79 (br s)	2.79 (br s)
2a	2.63 (m)	2.64 (m)	2.64 (m)	2.67 (dt, 15.2, 2.4)	2.67 (dt, 15.6, 2.4)	2.67 (dt, 15.6, 2.4)
2b	2.63 (m)	2.64 (m)	2.64 (m)	2.58 (ddd, 15.2, 6.5, 1.0)	2.58 (ddd, 15.2, 6.6, 0.8)	2.58 (ddd, 15.2, 6.6, 0.8)
3						
4	2.70 (d, 13.0)	2.70 (d, 13.1)	2.70 (d, 13.0)	2.70 (dt, 12.4, 1.1)	2.70 (dt, 12.4, 1.4)	2.70 (dt, 12.4, 1.1)
5	5.07 (s)	5.07 (s)	5.07 (s)	5.07 (d, 1.2)	5.07 (s)	5.07 (s)
6						
7	2.59 (d, 9.3)	2.59 (d, 9.3)	2.59 (d, 9.3)	2.59 (d, 9.3)	2.59 (d, 9.3)	2.59 (d, 9.3)
8						
9a	2.10 (dd, 15.6, 7.2)	2.10 (dd, 15.7, 7.3)	2.10 (dd, 15.5, 7.3)	2.10 (dd, 15.5, 7.3)	2.09 (dd, 15.6, 7.2)	2.10 (dd, 15.5, 7.2)
9b	2.51 (d, 15.6)	2.52 (d, 15.5)	2.52 (d, 15.6)	2.52 (d, 15.5)	2.52 (d, 15.6)	2.52 (d, 15.6)
10	5.13 (t, 7.2)	5.12 (t, 7.3)	5.12 (t, 7.4)	5.13 (t, 7.5)	5.12 (t, 7.4)	5.12 (t, 7.4)
11	3.42 (ddd, 12.3, 9.3, 7.2)	3.42 (ddd, 12.2, 9.3, 7.5)	3.42 (ddd, 12.1, 9.2, 7.6)	3.42 (ddd, 12.0, 9.3, 7.6)	3.42 (ddd, 12.1, 9.2, 7.6)	3.42 (ddd, 12.1, 9.4, 7.6)
12	3.02 (dd, 12.3, 2.5)	3.02 (dd, 12.2, 2.5)	3.02 (dd, 12.0, 2.4)	3.02 (dd, 12.0, 2.4)	3.02 (dd, 12.3, 2.0)	3.02 (dd, 12.1, 2.4)
13	2.24 (tt, 13.0, 2.5)	2.25 (tt, 12.6, 2.7)	2.25 (tt, 12.4, 2.8)	2.25 (tt, 12.3, 3.2)	2.24 (tt, 12.4, 2.8)	2.26 (tt, 12.3, 2.8)
14a	3.00 (m)	3.00 (m)	3.00 (m)	3.01 (ddd, 14.0, 12.1, 5.3)	3.00 (m)	3.00 (m)
14b	1.79 (m)	1.79 (dq, 13.0, 2.6)	1.78 (dq, 13.0, 2.8)	1.78 (dq, 13.9, 2.9)	1.78 (dq, 13.9, 2.9)	1.78 (dq, 14.0, 3.1)
15						
16a	4.62 (s)	4.62 (s)	4.62 (s)	4.62 (p, 1.4)	4.62 (s)	4.62 (s)
16b	4.94 (s)	4.94 (s)	4.94 (s)	4.94 (qd, 1.4, 0.7)	4.94 (m)	4.94 (s)
17	1.71 (s)	1.71 (s)	1.71 (s)	1.71 (dt, 1.4, 0.7)	1.71 (dt, 1.3, 0.7)	1.71 (s)
18	1.28 (s)	1.28 (s)	1.28 (s)	1.28 (s)	1.29 (s)	1.29 (s)
19						

Table S4. ^{13}C NMR ($CDCl_3$, 25 °C) Comparison of Natural and Synthetic (+)-Ineleganolide (**4**).

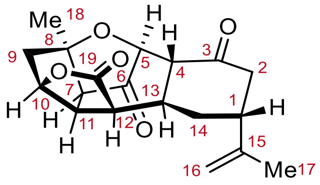

(+)-ineleganolide (**4**)

No.	Isolation[3] δ ^{13}C (ppm)	Synthetic (Wood)[4] δ ^{13}C (ppm)	Synthetic (Stoltz)[5] δ ^{13}C (ppm)	Synthetic (Fürstner)[6] δ ^{13}C (ppm)	Synthetic (Sarlah)[7] δ ^{13}C (ppm)	Synthetic (this work) δ ^{13}C (ppm)
1	40.2	40.2	40.4	40.4	40.4	40.4
2	44.3	44.3	44.4	44.4	44.4	44.4
3	206.2	206.3	206.4	206.5	206.4	206.4
4	49.7	49.7	49.8	49.8	49.8	49.9
5	77.4	77.3	77.4	77.5	77.5	77.5
6	211.9	212.1	212.2	212.2	212.2	212.2
7	62.4	62.4	62.5	62.5	62.5	62.5
8	90.9	91.0	91.1	91.1	91.1	91.1
9	45.4	45.4	45.5	45.5	45.5	45.5
10	83.0	83.0	83.1	83.1	83.1	83.1
11	43.7	43.6	43.8	43.8	43.8	43.8
12	46.9	46.9	47.0	47.1	47.1	47.1
13	33.1	33.1	33.2	33.2	33.2	33.3
14	32.6	32.6	32.7	32.7	32.7	32.7
15	145.8	145.9	146.0	146.0	146.0	146.0
16	113.6	113.7	113.8	113.8	113.8	113.9
17	22.5	22.5	22.7	22.7	22.7	22.7
18	20.1	20.1	20.2	20.2	20.2	20.2
19	175.8	175.9	176.1	176.1	176.1	176.1

Figure S1. Comparison of the ^{1}H NMR Spectra of Reported and Synthetic (+)-Ineleganolide (**4**).

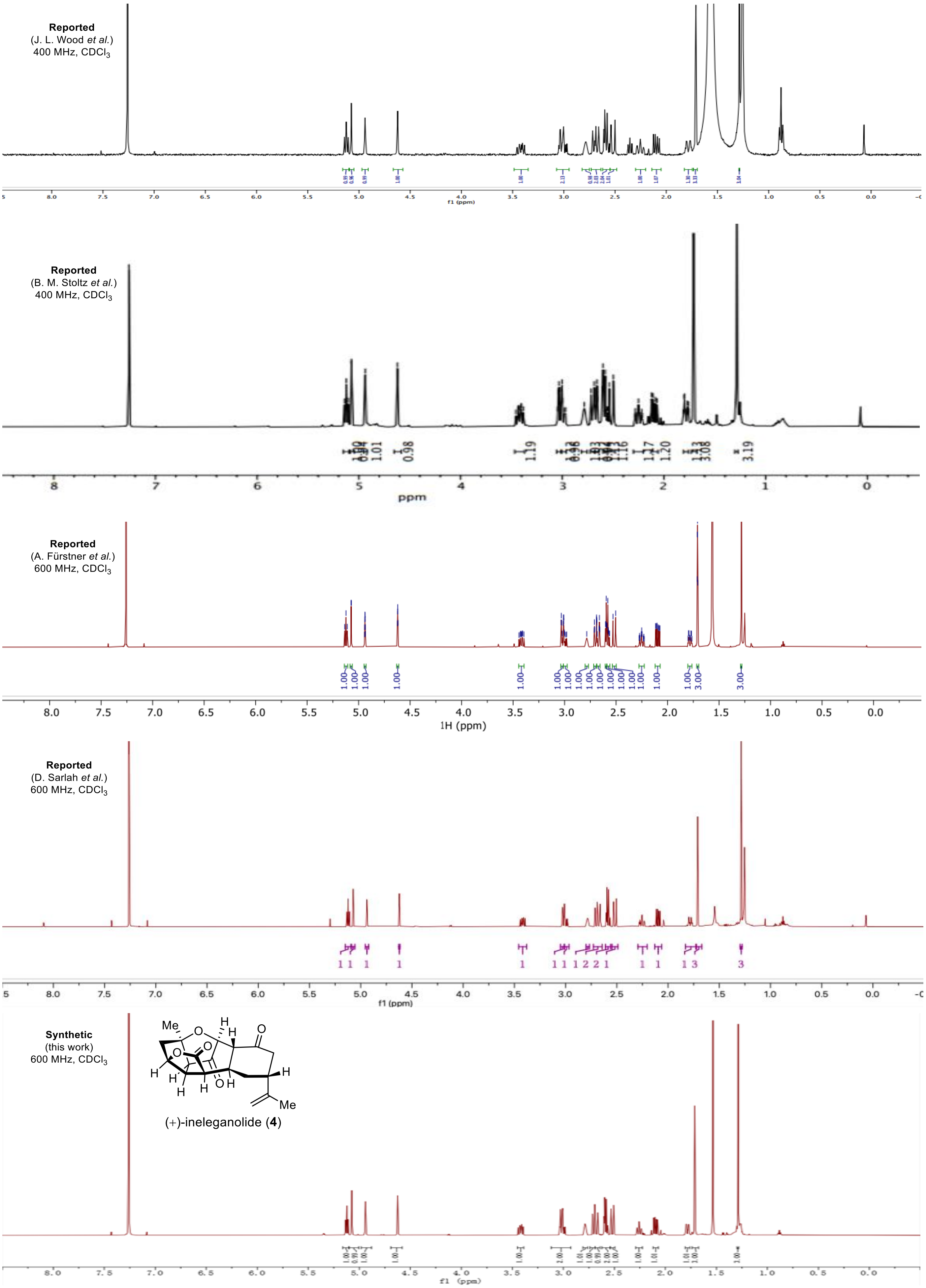

Figure S2. Comparison of the ^{13}C NMR Spectra of Reported and Synthetic (+)-Ineleganolide (**4**).

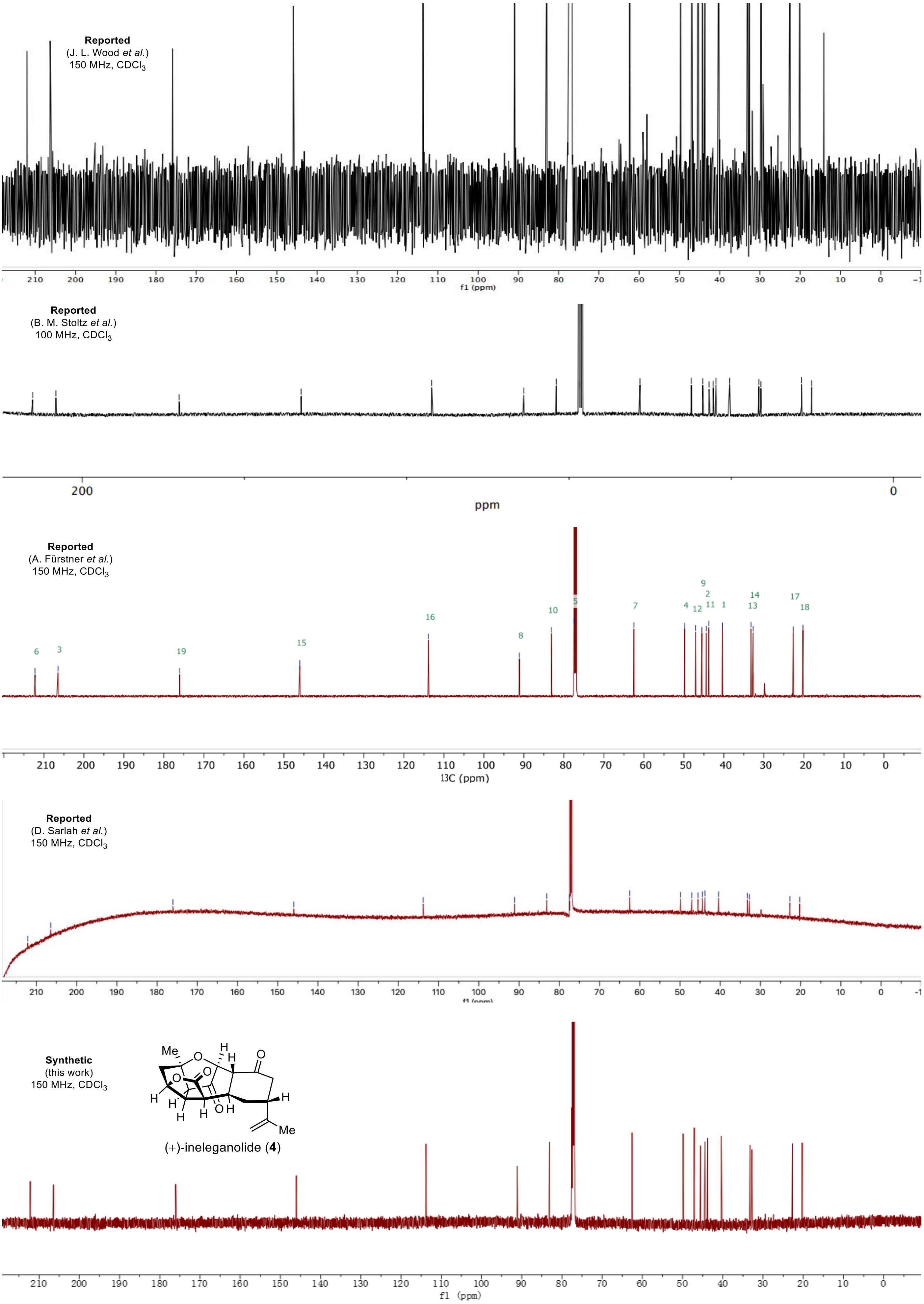

IV) Computational Details and Coordinates of Computed Structures

All geometry optimizations were carried out using the Gaussian 16 program at the Molecular Graphics and Computation Facility at the University of California, Berkeley.[8] A conformer search was conducted with Maestro for all structures; for each structure, a subset of these generated conformers was optimized with density functional theory (DFT), with only the lowest energy conformer reported herein.[9-11] Geometry optimizations and vibrational analyses were conducted using Zhao and Truhlar's M06-2X functional with Grimme's D3 dispersion correction, the Ahlrichs def2-TZVP basis set, and the Solvation Model based on Density (SMD) with MeCN as the solvent.[12-16] Intrinsic reaction coordinate (IRC) analyses were carried out at the B3LYP-D3(BJ)/6-31G/SMD(MeCN) level of theory to ensure correct identification of transition states.[17-20] All optimized minima and transition states were confirmed by the appropriate number and character of imaginary vibrational modes; these structures were visualized using CYLview20.[21]

Cartesian Coordinates and Free Energies of Calculated Structures

20B-H⁺ [M062X-D3/def2-TZVP/SMD(MeCN)]

Free energy: -1073.199882 a.u.

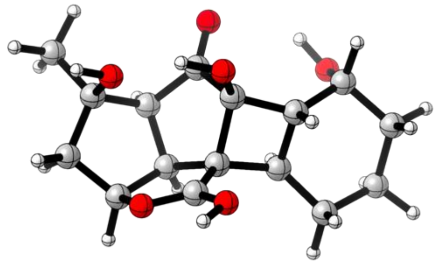

C	0.962301	0.460787	-1.288496	C	-2.922649	-1.240830	0.490742
C	1.511029	-0.926309	-0.907450	H	-2.053667	0.427719	1.469712
C	2.878959	-0.663203	-0.208101	H	-1.542631	-0.068426	-1.470910
C	3.348064	0.658934	-0.821226	O	-2.705151	-2.141186	-0.586729
C	2.099578	1.467273	-1.084659	H	4.084399	1.171105	-0.201037
C	0.478608	-1.533516	0.027751	H	3.799574	0.465908	-1.796848
C	-0.284836	-0.420757	0.787746	H	2.208770	2.209994	-1.868078
C	-0.100159	0.825631	-0.245178	H	3.443843	-0.271073	1.641753
O	1.665651	2.268831	0.104418	H	4.069371	-1.985584	-1.417230
C	0.464729	1.984000	0.422226	H	4.790787	-1.568263	0.147348
O	-0.139329	2.641579	1.334913	H	3.440156	-2.716405	0.073626
O	2.607042	-0.446506	1.186454	H	1.087138	-0.438483	2.131355
C	3.855260	-1.806992	-0.363742	H	-2.851734	2.218531	0.049790
H	1.671894	-1.576515	-1.764505	H	-2.620287	2.179509	-1.706498
H	0.556723	0.495464	-2.295132	H	-4.873847	1.245623	-1.038277
O	0.265290	-2.702262	0.195619	H	-3.873111	0.101353	-1.912437
C	-1.591012	0.600556	-0.606021	H	-4.421224	0.151615	1.093220
C	-1.794979	-0.241298	0.641470	H	-4.989481	-1.041613	-0.066056
O	0.119747	-0.335314	2.103090	H	-3.096486	-1.799781	1.414595
C	-2.737868	1.560454	-0.816049	H	-2.001953	-2.751588	-0.332293
C	-3.969064	0.640145	-0.965993	H	0.412073	3.359573	1.714917
C	-4.155679	-0.379841	0.175459				

20-TS1 [M062X-D3/def2-TZVP/SMD(MeCN)]

Free energy: -1073.175712 a.u.

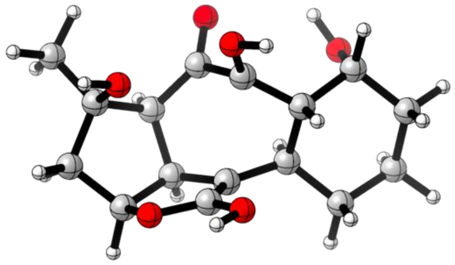

C	1.081551	0.703023	-1.321806
C	1.498758	-0.759121	-0.995829
C	2.826979	-0.650817	-0.181676
C	3.426719	0.674556	-0.649082
C	2.250083	1.598234	-0.865866
C	0.478082	-1.512516	-0.188356
C	-0.384630	-0.818301	0.887579
C	-0.053194	1.173087	-0.459569
O	1.791568	2.211444	0.380373
C	0.484309	1.968585	0.493065
O	-0.130993	2.527305	1.522647
O	2.423984	-0.548012	1.182755
C	3.735517	-1.846669	-0.371190
H	1.685908	-1.342352	-1.896365
H	0.872617	0.803761	-2.385276
O	0.238673	-2.686986	-0.286601
C	-1.451898	0.656916	-0.636151
C	-1.699966	-0.188782	0.603979
O	-0.049848	-1.122683	2.077303
C	-2.625585	1.605389	-0.839914
C	-3.898303	0.762615	-0.982311
C	-4.114747	-0.194280	0.195811
C	-2.925690	-1.117736	0.455961
H	-1.905801	0.475297	1.453959
H	-1.424017	-0.017925	-1.498357
O	-2.780439	-1.992073	-0.645627
H	4.160146	1.061414	0.060093
H	3.920782	0.532725	-1.612692
H	2.459948	2.418581	-1.548509
H	3.206206	-0.381666	1.724521
H	4.029199	-1.949006	-1.416088
H	4.636571	-1.720968	0.233727
H	3.230732	-2.760614	-0.052366
H	-0.709471	-0.817946	2.739194
H	-2.705387	2.277003	0.020550
H	-2.470074	2.218680	-1.728856
H	-4.768792	1.413765	-1.072505
H	-3.834390	0.184785	-1.908214
H	-4.295946	0.379536	1.108355
H	-4.992289	-0.817968	0.018427
H	-3.083094	-1.682661	1.378390
H	-2.229171	-2.742389	-0.390827
H	0.504853	3.030890	2.058163

20-INT1 [M062X-D3/def2-TZVP/SMD(MeCN)]

Free energy: -1073.190400 a.u.

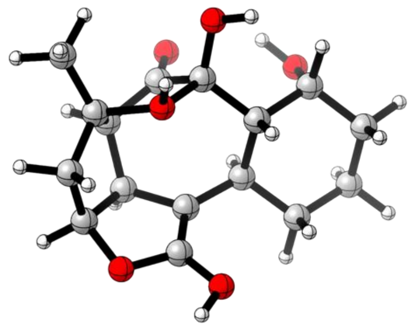

C	-1.426478	0.621603	1.305162	C	2.729461	-0.842664	-0.490003
C	-1.590487	-0.905197	1.077019	H	1.216091	0.190737	-1.599762
C	-2.273271	-1.000331	-0.280173	H	1.310058	0.388301	1.435524
C	-3.155365	0.228433	-0.408119	O	2.959888	-1.449015	0.770442
C	-2.685596	1.239422	0.653734	H	-3.119133	0.650421	-1.413048
C	-0.290329	-1.621438	0.893673	H	-4.182088	-0.083041	-0.218702
C	0.244285	-1.359880	-0.531843	H	-3.473957	1.492104	1.355838
C	-0.336258	1.314523	0.507900	H	-1.165895	-1.180694	-2.072965
O	-2.248955	2.466596	0.044198	H	-3.736290	-2.493254	0.022675
C	-0.908919	2.336355	-0.129593	H	-3.266812	-2.295156	-1.671845
O	-0.361518	3.270513	-0.908892	H	-2.187914	-3.144703	-0.534796
O	-1.033125	-0.803002	-1.175391	H	1.151178	-2.403507	-1.876635
C	-2.893469	-2.319620	-0.646232	H	1.961251	2.397855	-0.745794
H	-2.190996	-1.364887	1.859438	H	1.953813	2.742022	0.979141
H	-1.329667	0.810976	2.375301	H	4.257348	2.200954	0.153068
O	0.302180	-2.320630	1.661724	H	3.713352	1.037865	1.353670
C	1.085185	0.843672	0.463287	H	3.668351	0.657004	-1.678703
C	1.306312	-0.258230	-0.603943	H	4.746906	-0.160593	-0.549096
O	0.538978	-2.535368	-1.131835	H	2.893649	-1.575316	-1.287781
C	2.115839	1.954368	0.241031	H	2.345270	-2.182215	0.906492
C	3.534540	1.404946	0.339899	H	-1.063850	3.831534	-1.273527
C	3.751176	0.272511	-0.658541				

16 [M062X-D3/def2-TZVP/SMD(MeCN)]

Free energy: -1072.833282 a.u.

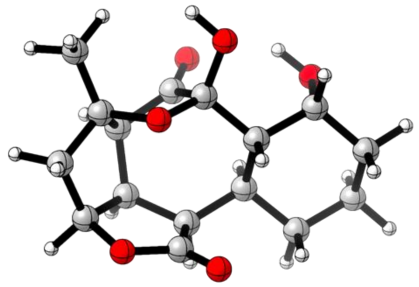

C	-1.445552	0.852089	1.332054	O	2.739552	-1.791959	0.417422
C	-1.639963	-0.683800	1.169310	H	-1.576008	1.099047	2.380894
C	-2.307810	-0.808101	-0.209559	H	-2.226208	-1.098526	1.986144
C	-3.270932	0.358925	-0.271347	H	-3.535091	0.633589	-1.292684
C	-2.569843	1.492661	0.461891	H	-4.181451	0.077941	0.259409
C	-0.415188	-1.496550	0.890123	H	-3.253712	2.111566	1.037369
C	-0.008761	-1.159569	-0.563700	H	1.068132	0.415170	-1.494002
C	1.178794	-0.180785	-0.582476	H	1.274903	0.146043	1.538836
C	1.151244	0.759165	0.639276	H	0.057505	2.346130	1.520957
C	-0.161064	1.541460	0.810432	H	-3.813356	-2.303330	0.104720
O	-1.931524	2.355437	-0.493092	H	-3.241583	-2.181261	-1.569836
C	-0.599513	2.288128	-0.433793	H	-2.253300	-2.983239	-0.334351
O	0.086327	2.828015	-1.259063	H	-0.395457	-2.931882	-1.273289
C	-2.935191	-2.149962	-0.523461	H	2.240292	2.387117	-0.256609
O	-1.171511	-0.535583	-1.079673	H	2.318765	2.344337	1.501278
O	0.098866	-2.350157	1.560796	H	4.495513	1.688110	0.460238
O	0.335139	-2.299150	-1.289466	H	3.817027	0.377071	1.416475
C	2.345188	1.722087	0.603249	H	3.607975	0.665886	-1.617880
C	3.669916	0.975805	0.513936	H	4.615738	-0.501977	-0.764257
C	3.684065	0.064859	-0.707029	H	2.528171	-1.492505	-1.612670
C	2.525188	-0.917899	-0.683818	H	1.993894	-2.403193	0.483345

21A-H$^+$ [M062X-D3/def2-TZVP/SMD(MeCN)]

Free energy: -1073.212237 a.u.

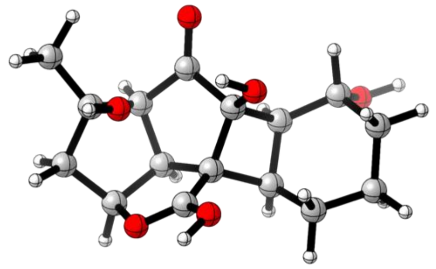

C	1.059197	0.226454	-1.372931
C	1.660290	-1.077089	-0.809803
C	2.883397	-0.609831	0.016711
C	3.417956	0.557210	-0.813368
C	2.177828	1.281965	-1.294057
C	0.639195	-1.655389	0.141841
C	-0.432433	-0.606246	0.431260
C	-0.011148	0.687159	-0.359352
O	1.730535	2.222762	-0.231434
C	0.647943	1.824215	0.298060
O	0.187248	2.420835	1.327987
O	0.649624	-2.751018	0.631164
C	-1.484932	0.798945	-0.868393
C	-1.639754	-0.704969	-0.536983
C	-2.399375	1.690597	-0.033721
C	-3.818509	1.129090	-0.020968
C	-3.813744	-0.219078	0.693388
C	-2.973445	-1.250480	-0.052085
O	-3.645795	-1.678229	-1.237150
H	-1.337673	-1.307760	-1.396659
H	-1.546894	1.053624	-1.925607
H	0.651268	0.121383	-2.374084
H	1.972039	-1.788646	-1.570848
C	3.887522	-1.696168	0.313798
O	2.314009	-0.099910	1.241517
O	-0.792808	-0.497583	1.759900
H	4.101856	1.199661	-0.257343
H	3.938097	0.169883	-1.691612
H	2.313767	1.899020	-2.175055
H	-2.057419	1.726486	1.001988
H	-2.366483	2.709759	-0.422668
H	-4.483482	1.822687	0.495136
H	-4.195414	1.014751	-1.041254
H	-3.410159	-0.092934	1.700924
H	-4.826812	-0.616542	0.798161
H	-2.784748	-2.114776	0.594349
H	-4.536527	-1.955453	-0.990827
H	4.302395	-2.093750	-0.612592
H	4.704943	-1.292645	0.915764
H	3.411437	-2.506626	0.867315
H	3.031176	0.192124	1.821017
H	0.010840	-0.490564	2.302859
H	0.765358	3.157149	1.624785

21-TS1 [M062X-D3/def2-TZVP/SMD(MeCN)]

Free energy: -1073.176005 a.u.

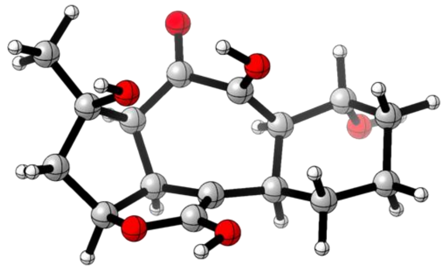

C	1.162659	0.401065	-1.368587	H	2.020633	-1.614907	-1.652237
C	1.644895	-0.983283	-0.847599	C	3.728413	-1.858726	0.390116
C	2.812238	-0.684564	0.131193	O	2.155564	-0.288288	1.351210
C	3.491888	0.501590	-0.543053	O	-0.730974	-0.865334	1.724925
C	2.366452	1.348242	-1.100639	H	4.133564	1.056985	0.142312
C	0.647745	-1.748629	-0.061909	H	4.103714	0.130102	-1.367077
C	-0.572817	-1.020786	0.479504	H	2.656456	1.936977	-1.967745
C	0.040146	1.067110	-0.581394	H	-1.915151	1.544237	1.234049
O	1.912137	2.282291	-0.092148	H	-2.248806	2.645205	-0.086827
C	0.605979	2.087063	0.100808	H	-4.349780	1.786327	0.880414
O	0.059132	2.943806	0.952034	H	-4.193756	1.176961	-0.762204
O	0.664972	-2.920524	0.206174	H	-3.403144	-0.341492	1.774919
C	-1.446762	0.775814	-0.731902	H	-4.853457	-0.659613	0.835394
C	-1.644562	-0.756346	-0.493942	H	-2.968315	-2.232575	0.334973
C	-2.314935	1.600556	0.218158	H	-4.616343	-1.600535	-1.253095
C	-3.761437	1.127702	0.240525	H	4.186121	-2.199797	-0.538553
C	-3.823761	-0.299482	0.766217	H	4.522104	-1.554515	1.076528
C	-3.055344	-1.248767	-0.138433	H	3.179941	-2.686149	0.841930
O	-3.688798	-1.378747	-1.403709	H	2.827843	-0.042014	2.000896
H	-1.346037	-1.274931	-1.409364	H	0.109938	-1.000763	2.223279
H	-1.761442	0.969328	-1.761666	H	0.727588	3.594912	1.221758
H	0.927284	0.329256	-2.429954				

21-INT1 [M062X-D3/def2-TZVP/SMD(MeCN)]

Free energy: -1073.179036 a.u.

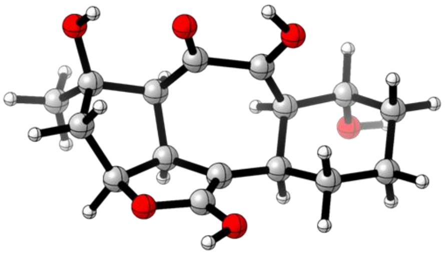

C	1.135770	0.433479	-1.057992	H	1.042606	0.312673	-2.137563
C	1.505231	-0.945503	-0.427527	H	1.209657	-1.779237	-1.072990
C	3.043166	-0.884270	-0.250628	H	2.958627	0.671191	1.245437
C	3.264051	0.564459	0.200285	H	4.305541	0.868548	0.109223
C	2.316829	1.374158	-0.672974	H	2.807807	1.795883	-1.546668
C	0.750517	-1.123816	0.845053	H	-0.996058	-1.344734	-1.262307
C	-0.807752	-1.157131	0.767357	H	-1.750378	0.796376	-1.703854
C	-1.562088	-0.893841	-0.440878	H	0.366623	3.879419	1.100388
C	-1.469471	0.680118	-0.651854	H	-2.221995	1.314857	1.271567
C	-0.053534	1.145226	-0.455707	H	-2.455588	2.462913	-0.032245
O	1.732661	2.476117	0.055454	H	-4.610364	1.426851	0.611877
C	0.405818	2.255646	0.140216	H	-4.195808	1.021106	-1.047077
O	-0.258394	3.200780	0.798876	H	-3.779775	-0.759629	1.404284
O	1.168405	-1.264642	1.963634	H	-4.983561	-1.011473	0.154193
O	-1.362014	-1.379350	1.870001	H	-2.983578	-2.468405	-0.116237
C	-2.496840	1.402160	0.214432	H	-4.251099	-1.659058	-1.954480
C	-3.906151	0.867001	-0.004493	H	3.348689	-0.478125	-2.364702
C	-3.982754	-0.613722	0.340623	H	4.786446	-1.024686	-1.487074
C	-2.996322	-1.434820	-0.478507	H	3.504803	-2.180876	-1.902325
O	-3.322701	-1.411721	-1.859461	H	3.245247	-1.656621	1.531204
C	3.708909	-1.156002	-1.589882	H	-0.673194	-1.483202	2.583848
O	3.566177	-1.844013	0.640290				

21-TS2 [M062X-D3/def2-TZVP/SMD(MeCN)]

Free energy: -1073.161893 a.u.

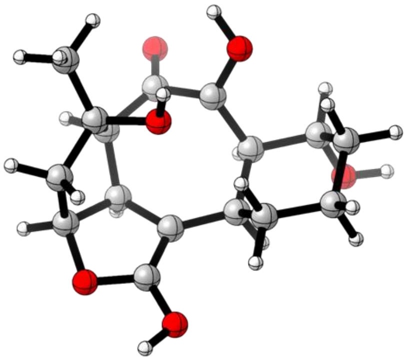

C	1.526575	0.305579	-1.241704	H	1.509959	0.352327	-2.332487
C	1.663479	-1.162708	-0.790340	H	2.481335	-1.631028	-1.337426
C	1.993793	-1.028657	0.703294	H	2.619119	0.864772	1.571640
C	2.914592	0.200020	0.759883	H	3.937813	-0.124761	0.941072
C	2.791763	0.911820	-0.606508	H	3.686866	0.817857	-1.214681
C	0.460750	-2.023895	-0.859991	H	-1.334285	-0.561834	-1.712091
C	-0.714083	-1.506800	-0.030566	H	-1.548315	1.656427	-1.113331
C	-1.485360	-0.375110	-0.637680	H	1.427176	4.080353	0.433032
C	-0.987532	1.067949	-0.380773	H	3.488434	-2.546378	0.893676
C	0.491545	1.247711	-0.616613	H	2.748881	-2.056790	2.424990
O	2.512824	2.309577	-0.455351	H	1.857090	-3.106546	1.307124
C	1.163423	2.361944	-0.313190	H	-0.864753	-3.186669	0.837342
O	0.696172	3.550287	0.078031	H	-0.929668	0.946616	1.777567
C	2.550486	-2.263836	1.370845	H	-1.108717	2.573681	1.148091
O	0.664475	-0.737915	1.250463	H	-3.217594	1.784540	2.193258
O	0.318332	-3.069142	-1.430499	H	-3.438146	2.114274	0.481680
O	-1.350780	-2.344643	0.704904	H	-3.002841	-0.633532	1.748962
C	-1.432015	1.543475	1.013251	H	-4.508224	-0.052721	1.053580
C	-2.942180	1.448234	1.192433	H	-3.288096	-1.552671	-0.491823
C	-3.420136	0.019190	0.976891	H	-4.545423	0.258288	-1.304233
C	-3.000870	-0.503063	-0.389835	H	0.576225	-1.096704	2.151367
O	-3.590050	0.248010	-1.441760				

21-INT2 [M062X-D3/def2-TZVP/SMD(MeCN)]

Free energy: -1073.194246 a.u.

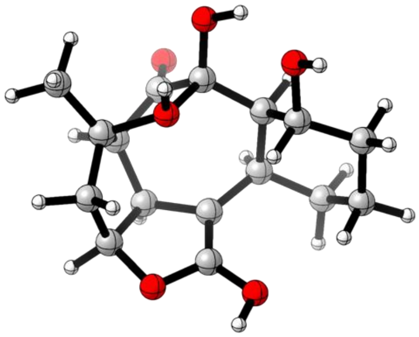

C	-1.265335	1.272388	1.076001	H	-1.385007	1.642336	2.096089
C	-2.065798	-0.043291	0.919484	H	-3.011638	0.005409	1.455813
C	-2.282252	-0.163656	-0.583211	H	-1.748074	1.362165	-2.034624
C	-2.347336	1.255261	-1.129749	H	-3.381805	1.472888	-1.391231
C	-1.850084	2.192558	-0.011561	H	-2.628892	2.863103	0.338640
C	-1.277429	-1.256513	1.304710	H	1.562610	-1.875198	1.031705
C	-0.309604	-1.616138	0.164093	H	0.797230	-0.000504	2.209352
C	1.107068	-1.098122	0.407496	H	1.244150	3.521531	-1.297405
C	1.160540	0.234063	1.203383	H	-4.318262	-0.715738	-0.724189
C	0.201780	1.260596	0.665771	H	-3.356874	-1.186620	-2.132459
O	-0.752744	3.006128	-0.453500	H	-3.226817	-2.106083	-0.610824
C	0.371742	2.312317	-0.139106	H	0.365343	-3.160555	-0.700215
O	1.484029	2.829018	-0.661448	H	2.656886	1.662141	1.811669
C	-3.354615	-1.112343	-1.043280	H	3.058690	-0.017374	2.131336
O	-0.926881	-0.772992	-0.974927	H	3.236761	1.381330	-0.563507
O	-1.317325	-1.893795	2.314012	H	4.545261	0.750814	0.419730
O	-0.415473	-2.918153	-0.165392	H	3.941006	-0.760688	-1.503940
C	2.630602	0.658036	1.385022	H	3.735982	-1.571569	0.055178
C	3.505128	0.588150	0.132742	H	1.519268	-0.176019	-1.491882
C	3.367009	-0.755726	-0.573981	H	2.253935	-2.255503	-2.379180
C	1.906285	-1.006059	-0.888021	H	-0.990527	-1.305502	-1.797864
O	1.696467	-2.232642	-1.591363				

C4-*epi*-**16** [M062X-D3/def2-TZVP/SMD(MeCN)]

Free energy: -1072.827369 a.u.

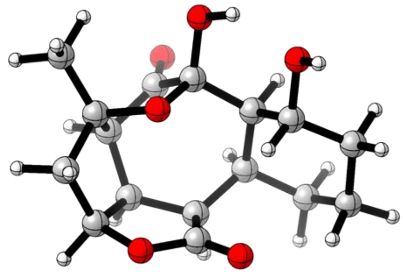

C	-1.308497	1.338579	1.100946	O	1.880960	-2.050449	-1.661931
C	-2.029353	-0.041291	1.036198	H	-1.570944	1.812572	2.041819
C	-2.302687	-0.219547	-0.464286	H	-2.921389	-0.043301	1.658828
C	-2.713578	1.158053	-0.938650	H	-2.554103	1.300775	-2.007678
C	-1.889991	2.132311	-0.106590	H	-3.775753	1.288116	-0.727414
C	-1.181539	-1.254756	1.263315	H	-2.454565	3.011182	0.194713
C	-0.344363	-1.414412	-0.019930	H	1.506221	-1.807697	0.882181
C	1.088047	-0.974879	0.304028	H	0.725064	-0.090678	2.201554
C	1.131551	0.249824	1.245342	H	0.552788	2.245330	1.590217
C	0.229682	1.456596	0.901313	H	-4.263047	-1.090600	-0.440492
O	-0.778219	2.602501	-0.880898	H	-3.338310	-1.403783	-1.921218
C	0.391877	2.131603	-0.446708	H	-2.954184	-2.286419	-0.438466
O	1.400193	2.351285	-1.061587	H	0.296963	-2.847496	-1.096238
C	-3.272504	-1.321387	-0.835018	H	2.627106	1.536993	2.130223
O	-0.959069	-0.516918	-0.935370	H	2.967433	-0.181263	2.225294
O	-1.167505	-2.011446	2.193346	H	3.324737	1.572149	-0.228842
O	-0.420520	-2.732073	-0.451337	H	4.564899	0.766309	0.722041
C	2.597152	0.605508	1.560547	H	4.025869	-0.455313	-1.419483
C	3.537875	0.683440	0.359977	H	3.763052	-1.439917	0.021640
C	3.411003	-0.555863	-0.520880	H	1.597397	-0.007532	-1.558286
C	1.969769	-0.819495	-0.930965	H	2.347363	-1.948313	-2.500006

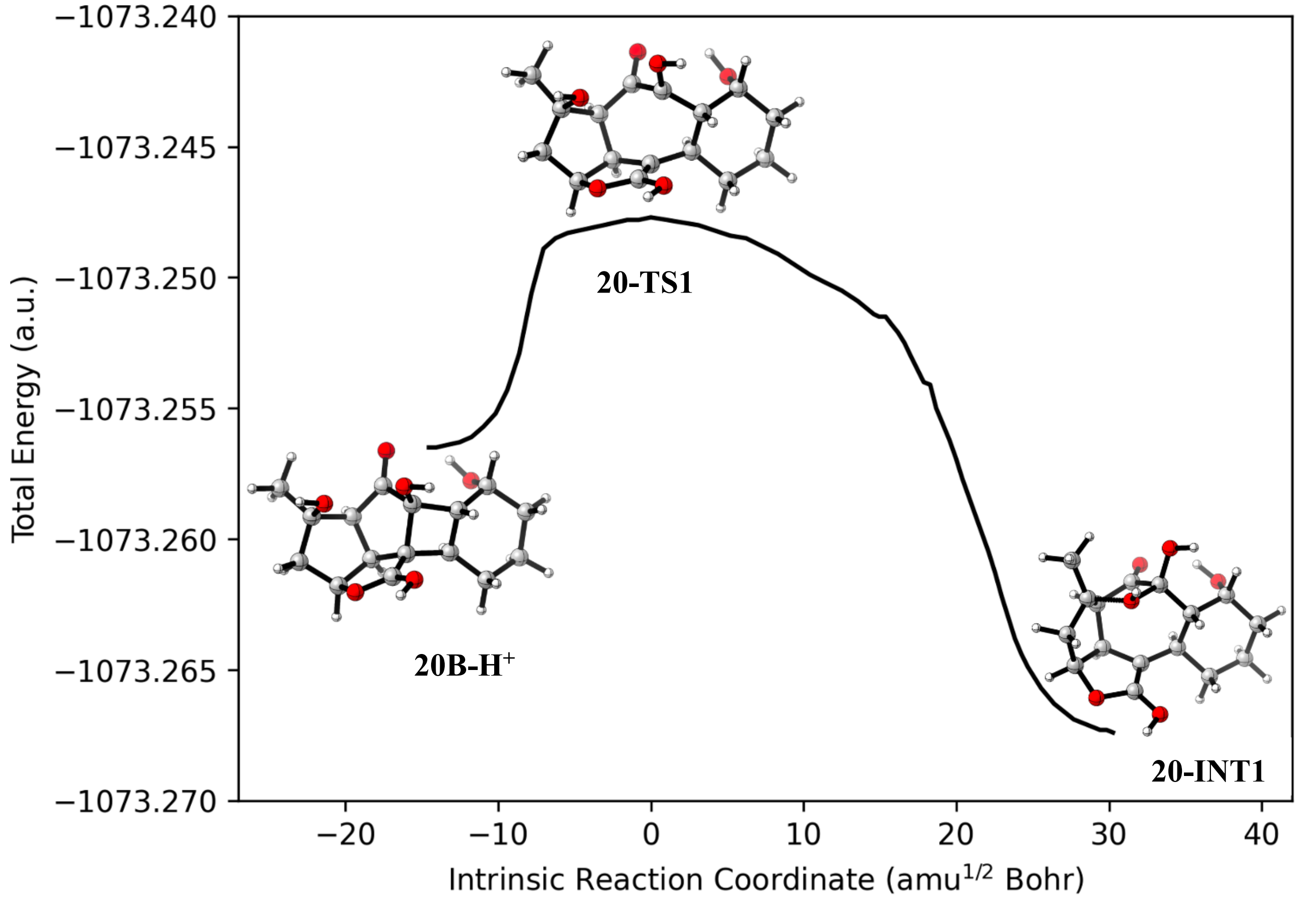

Figure S3. IRC (B3LYP-D3(BJ)/6-31G/SMD(MeCN)) connecting **20-TS1** to **20B-H**$^+$ and **20-INT1**.

V) Crystallographic Data

Bicycle 15: (grown via slow evaporation from EtOAc/hexanes at 23 ºC)

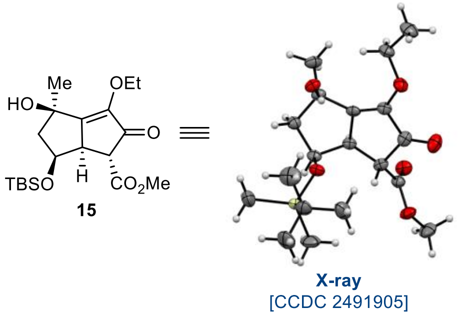

A colorless block 0.10 x 0.05 x 0.04 mm in size was mounted on a Cryoloop with Paratone oil. Data were collected in a nitrogen gas stream at 100(2) K using omega scans. Crystal-to-detector distance was 31.12 mm and exposure time was 1.00 seconds per frame at low angles and 3.00 seconds at high angles, using a scan width of 0.5°. Data collection was 100% complete to 74.000° in θ. A total of 27155 reflections were collected covering the indices -8<=h<=12, -13<=k<=13, -28<=l<=28. 5053 reflections were found to be symmetry independent, with an R_{int} of 0.0438. Indexing and unit cell refinement indicated a primitive, orthorhombic lattice. The space group was found to be P 21 21 21 (No. 19). The data were integrated using the CrysAlis^{Pro} 1.172.43.143a software program and scaled using the SCALE3 ABSPACK scaling algorithm. Solution by intrinsic phasing (SHELXT-2015) produced a heavy-atom phasing model consistent with the proposed structure. All non-hydrogen atoms were refined anisotropically by full-matrix least-squares (SHELXL-2014). All hydrogen atoms were placed using a riding model. Their positions were constrained relative to their parent atom using the appropriate HFIX command in SHELXL-2014. A solvent mask was used within Olex2 to mask the electron density associated with a disordered, partially occupied hexane fragment sitting on a special position. Additionally, a secondary position for the silicon atom was observed and refined at ~9% occupancy, but the associated secondary positions of the methyl and tert-butyl carbons could not be refined due to the weakly diffracting nature of a 9% occupied carbon atom.

Table 1. Crystal data and structure refinement for KYu10_Maimone.

Identification code	KYu10_Maimone	
Empirical formula	C19 H32 O6 Si	
Formula weight	384.53	
Temperature	100(2) K	
Wavelength	1.54184 Å	
Crystal system	Orthorhombic	
Space group	P 21 21 21	
Unit cell dimensions	a = 10.17850(10) Å	α= 90°.
	b = 10.8128(2) Å	β= 90°.
	c = 22.4574(4) Å	γ = 90°.
Volume	2471.62(7) Å^3	
Z	4	
Density (calculated)	1.033 Mg/m^3	
Absorption coefficient	1.056 mm^{-1}	
F(000)	832	
Crystal size	0.100 x 0.050 x 0.040 mm^3	
Theta range for data collection	4.539 to 74.484°.	
Index ranges	-8<=h<=12, -13<=k<=13, -28<=l<=28	
Reflections collected	27155	
Independent reflections	5053 [R(int) = 0.0438]	
Completeness to theta = 74.000°	99.9 %	
Absorption correction	Semi-empirical from equivalents	
Max. and min. transmission	1.00000 and 0.86685	
Refinement method	Full-matrix least-squares on F^2	
Data / restraints / parameters	5053 / 0 / 254	
Goodness-of-fit on F^2	1.048	
Final R indices [I>2sigma(I)]	R1 = 0.0329, wR2 = 0.0833	
R indices (all data)	R1 = 0.0354, wR2 = 0.0850	
Absolute structure parameter	0.007(11)	
Extinction coefficient	n/a	
Largest diff. peak and hole	0.236 and -0.182 e.Å^{-3}	

Table 2. Atomic coordinates (x 10^4) and equivalent isotropic displacement parameters ($Å^2$x 10^3) for KYu10_Maimone. U(eq) is defined as one third of the trace of the orthogonalized U^{ij} tensor.

	x	y	z	U(eq)
Si(1A)	2673(1)	3130(1)	9080(1)	27(1)
Si(1B)	2609(7)	2615(7)	8690(3)	37(3)
O(1)	3927(1)	3471(1)	8634(1)	30(1)
O(2)	7232(1)	3306(1)	8738(1)	31(1)
O(3)	7239(2)	2682(1)	7220(1)	34(1)
O(4)	4908(2)	2887(2)	6569(1)	45(1)
O(5)	3623(2)	5847(1)	6732(1)	36(1)
O(6)	2041(2)	4418(2)	6844(1)	38(1)
C(1)	4327(2)	4711(2)	8516(1)	28(1)
C(2)	5597(2)	4997(2)	8844(1)	30(1)
C(3)	6740(2)	4425(2)	8477(1)	27(1)
C(4)	7895(2)	5322(2)	8456(1)	36(1)
C(5)	6092(2)	4179(2)	7879(1)	25(1)
C(6)	4762(2)	4801(2)	7863(1)	25(1)
C(7)	1131(2)	3691(3)	8715(1)	54(1)
C(8)	2838(3)	3869(3)	9825(1)	50(1)
C(9)	2728(2)	1401(2)	9146(1)	41(1)
C(10)	2649(3)	818(3)	8519(1)	54(1)
C(11)	1545(3)	982(3)	9523(1)	59(1)
C(12)	4010(3)	1009(3)	9450(2)	61(1)
C(13)	6258(2)	3417(2)	7415(1)	28(1)
C(14)	8542(2)	2939(2)	7442(1)	36(1)
C(15)	9450(3)	2011(3)	7159(1)	50(1)
C(16)	5055(2)	3399(2)	7047(1)	31(1)
C(17)	3988(2)	4120(2)	7383(1)	28(1)
C(18)	3219(2)	4913(2)	6957(1)	29(1)
C(19)	1273(3)	5056(3)	6399(1)	48(1)

Table 3. Bond lengths [Å] and angles [°] for KYu10_Maimone.

Si(1A)-O(1)	1.6635(15)
Si(1A)-C(8)	1.862(3)
Si(1A)-C(7)	1.871(3)
Si(1A)-C(9)	1.876(3)
Si(1B)-O(1)	1.634(7)
O(1)-C(1)	1.426(2)
O(2)-C(3)	1.434(2)
O(2)-H(2)	0.8400
O(3)-C(13)	1.349(3)
O(3)-C(14)	1.444(3)
O(4)-C(16)	1.216(3)
O(5)-C(18)	1.202(3)
O(6)-C(18)	1.337(3)
O(6)-C(19)	1.445(3)
C(1)-C(2)	1.520(3)
C(1)-C(6)	1.534(3)
C(1)-H(1)	1.0000
C(2)-C(3)	1.555(3)
C(2)-H(2A)	0.9900
C(2)-H(2B)	0.9900
C(3)-C(5)	1.519(3)
C(3)-C(4)	1.526(3)
C(4)-H(4A)	0.9800
C(4)-H(4B)	0.9800
C(4)-H(4C)	0.9800
C(5)-C(13)	1.339(3)
C(5)-C(6)	1.512(3)
C(6)-C(17)	1.525(3)
C(6)-H(6)	1.0000
C(7)-H(7A)	0.9800
C(7)-H(7B)	0.9800
C(7)-H(7C)	0.9800
C(8)-H(8A)	0.9800
C(8)-H(8B)	0.9800
C(8)-H(8C)	0.9800
C(9)-C(12)	1.532(4)
C(9)-C(11)	1.541(3)
C(9)-C(10)	1.545(4)
C(10)-H(10A)	0.9800
C(10)-H(10B)	0.9800

C(10)-H(10C)	0.9800
C(11)-H(11A)	0.9800
C(11)-H(11B)	0.9800
C(11)-H(11C)	0.9800
C(12)-H(12A)	0.9800
C(12)-H(12B)	0.9800
C(12)-H(12C)	0.9800
C(13)-C(16)	1.478(3)
C(14)-C(15)	1.506(3)
C(14)-H(14A)	0.9900
C(14)-H(14B)	0.9900
C(15)-H(15A)	0.9800
C(15)-H(15B)	0.9800
C(15)-H(15C)	0.9800
C(16)-C(17)	1.535(3)
C(17)-C(18)	1.505(3)
C(17)-H(17)	1.0000
C(19)-H(19A)	0.9800
C(19)-H(19B)	0.9800
C(19)-H(19C)	0.9800

O(1)-Si(1A)-C(8)	112.16(11)
O(1)-Si(1A)-C(7)	107.92(11)
C(8)-Si(1A)-C(7)	109.24(14)
O(1)-Si(1A)-C(9)	104.19(9)
C(8)-Si(1A)-C(9)	110.78(13)
C(7)-Si(1A)-C(9)	112.48(14)
C(1)-O(1)-Si(1B)	141.4(3)
C(1)-O(1)-Si(1A)	122.61(13)
C(3)-O(2)-H(2)	109.5
C(13)-O(3)-C(14)	117.00(16)
C(18)-O(6)-C(19)	115.23(19)
O(1)-C(1)-C(2)	110.11(16)
O(1)-C(1)-C(6)	108.64(17)
C(2)-C(1)-C(6)	101.82(16)
O(1)-C(1)-H(1)	111.9
C(2)-C(1)-H(1)	111.9
C(6)-C(1)-H(1)	111.9
C(1)-C(2)-C(3)	107.36(17)
C(1)-C(2)-H(2A)	110.2
C(3)-C(2)-H(2A)	110.2
C(1)-C(2)-H(2B)	110.2

C(3)-C(2)-H(2B)	110.2
H(2A)-C(2)-H(2B)	108.5
O(2)-C(3)-C(5)	111.39(16)
O(2)-C(3)-C(4)	106.28(16)
C(5)-C(3)-C(4)	114.75(18)
O(2)-C(3)-C(2)	112.38(17)
C(5)-C(3)-C(2)	102.30(16)
C(4)-C(3)-C(2)	109.90(17)
C(3)-C(4)-H(4A)	109.5
C(3)-C(4)-H(4B)	109.5
H(4A)-C(4)-H(4B)	109.5
C(3)-C(4)-H(4C)	109.5
H(4A)-C(4)-H(4C)	109.5
H(4B)-C(4)-H(4C)	109.5
C(13)-C(5)-C(6)	111.62(18)
C(13)-C(5)-C(3)	137.77(19)
C(6)-C(5)-C(3)	109.39(17)
C(5)-C(6)-C(17)	105.33(16)
C(5)-C(6)-C(1)	101.97(16)
C(17)-C(6)-C(1)	119.71(17)
C(5)-C(6)-H(6)	109.7
C(17)-C(6)-H(6)	109.7
C(1)-C(6)-H(6)	109.7
Si(1A)-C(7)-H(7A)	109.5
Si(1A)-C(7)-H(7B)	109.5
H(7A)-C(7)-H(7B)	109.5
Si(1A)-C(7)-H(7C)	109.5
H(7A)-C(7)-H(7C)	109.5
H(7B)-C(7)-H(7C)	109.5
Si(1A)-C(8)-H(8A)	109.5
Si(1A)-C(8)-H(8B)	109.5
H(8A)-C(8)-H(8B)	109.5
Si(1A)-C(8)-H(8C)	109.5
H(8A)-C(8)-H(8C)	109.5
H(8B)-C(8)-H(8C)	109.5
C(12)-C(9)-C(11)	109.8(2)
C(12)-C(9)-C(10)	109.7(2)
C(11)-C(9)-C(10)	109.9(2)
C(12)-C(9)-Si(1A)	109.67(19)
C(11)-C(9)-Si(1A)	108.3(2)
C(10)-C(9)-Si(1A)	109.49(18)
C(9)-C(10)-H(10A)	109.5

C(9)-C(10)-H(10B)	109.5
H(10A)-C(10)-H(10B)	109.5
C(9)-C(10)-H(10C)	109.5
H(10A)-C(10)-H(10C)	109.5
H(10B)-C(10)-H(10C)	109.5
C(9)-C(11)-H(11A)	109.5
C(9)-C(11)-H(11B)	109.5
H(11A)-C(11)-H(11B)	109.5
C(9)-C(11)-H(11C)	109.5
H(11A)-C(11)-H(11C)	109.5
H(11B)-C(11)-H(11C)	109.5
C(9)-C(12)-H(12A)	109.5
C(9)-C(12)-H(12B)	109.5
H(12A)-C(12)-H(12B)	109.5
C(9)-C(12)-H(12C)	109.5
H(12A)-C(12)-H(12C)	109.5
H(12B)-C(12)-H(12C)	109.5
C(5)-C(13)-O(3)	135.1(2)
C(5)-C(13)-C(16)	109.81(18)
O(3)-C(13)-C(16)	115.04(18)
O(3)-C(14)-C(15)	106.8(2)
O(3)-C(14)-H(14A)	110.4
C(15)-C(14)-H(14A)	110.4
O(3)-C(14)-H(14B)	110.4
C(15)-C(14)-H(14B)	110.4
H(14A)-C(14)-H(14B)	108.6
C(14)-C(15)-H(15A)	109.5
C(14)-C(15)-H(15B)	109.5
H(15A)-C(15)-H(15B)	109.5
C(14)-C(15)-H(15C)	109.5
H(15A)-C(15)-H(15C)	109.5
H(15B)-C(15)-H(15C)	109.5
O(4)-C(16)-C(13)	127.0(2)
O(4)-C(16)-C(17)	125.3(2)
C(13)-C(16)-C(17)	107.70(17)
C(18)-C(17)-C(6)	116.33(17)
C(18)-C(17)-C(16)	110.16(18)
C(6)-C(17)-C(16)	103.17(17)
C(18)-C(17)-H(17)	109.0
C(6)-C(17)-H(17)	109.0
C(16)-C(17)-H(17)	109.0
O(5)-C(18)-O(6)	124.2(2)

O(5)-C(18)-C(17)	124.65(19)
O(6)-C(18)-C(17)	111.07(18)
O(6)-C(19)-H(19A)	109.5
O(6)-C(19)-H(19B)	109.5
H(19A)-C(19)-H(19B)	109.5
O(6)-C(19)-H(19C)	109.5
H(19A)-C(19)-H(19C)	109.5
H(19B)-C(19)-H(19C)	109.5

Symmetry transformations used to generate equivalent atoms:

Table 4. Anisotropic displacement parameters (Å^{2}x 10^3) for KYu10_Maimone. The anisotropic displacement factor exponent takes the form: $-2\pi^2[h^2a^{*2}U^{11} + ... + 2 h k a^* b^* U^{12}]$

	U^{11}	U^{22}	U^{33}	U^{23}	U^{13}	U^{12}
Si(1A)	18(1)	37(1)	26(1)	-2(1)	2(1)	-2(1)
Si(1B)	26(3)	48(5)	36(5)	2(3)	-4(3)	-4(3)
O(1)	24(1)	29(1)	38(1)	6(1)	4(1)	1(1)
O(2)	29(1)	24(1)	39(1)	3(1)	-7(1)	1(1)
O(3)	33(1)	32(1)	38(1)	-3(1)	3(1)	9(1)
O(4)	56(1)	34(1)	43(1)	-9(1)	-13(1)	11(1)
O(5)	37(1)	28(1)	42(1)	9(1)	-2(1)	1(1)
O(6)	26(1)	48(1)	40(1)	9(1)	-11(1)	-4(1)
C(1)	24(1)	26(1)	34(1)	2(1)	1(1)	4(1)
C(2)	29(1)	30(1)	30(1)	1(1)	0(1)	2(1)
C(3)	24(1)	23(1)	34(1)	3(1)	-2(1)	-2(1)
C(4)	26(1)	29(1)	55(1)	-4(1)	-3(1)	-3(1)
C(5)	21(1)	23(1)	32(1)	7(1)	2(1)	-2(1)
C(6)	24(1)	21(1)	31(1)	2(1)	0(1)	2(1)
C(7)	23(1)	70(2)	68(2)	2(2)	-3(1)	8(1)
C(8)	47(1)	64(2)	37(1)	-15(1)	10(1)	-5(1)
C(9)	40(1)	45(1)	40(1)	2(1)	9(1)	-10(1)
C(10)	55(2)	49(1)	58(2)	-18(1)	17(1)	-15(1)
C(11)	61(2)	65(2)	52(2)	1(1)	21(1)	-26(2)
C(12)	59(2)	56(2)	68(2)	26(2)	-4(2)	5(2)
C(13)	27(1)	24(1)	34(1)	4(1)	1(1)	3(1)
C(14)	27(1)	39(1)	42(1)	0(1)	6(1)	6(1)
C(15)	37(1)	54(2)	59(2)	-3(1)	10(1)	16(1)
C(16)	38(1)	20(1)	34(1)	1(1)	-4(1)	2(1)
C(17)	27(1)	24(1)	34(1)	5(1)	-5(1)	0(1)
C(18)	25(1)	30(1)	31(1)	0(1)	-1(1)	3(1)
C(19)	35(1)	63(2)	45(1)	9(1)	-15(1)	5(1)

Table 5. Hydrogen coordinates (x 10^4) and isotropic displacement parameters ($Å^2$x 10^3) for KYu10_Maimone.

	x	y	z	U(eq)
H(2)	6720	2719	8658	46
H(1)	3619	5320	8613	34
H(2A)	5575	4634	9249	36
H(2B)	5716	5902	8881	36
H(4A)	8195	5497	8862	54
H(4B)	7618	6094	8265	54
H(4C)	8615	4953	8227	54
H(6)	4867	5689	7749	30
H(7A)	1127	4597	8710	81
H(7B)	368	3390	8939	81
H(7C)	1092	3379	8306	81
H(8A)	3660	3600	10012	74
H(8B)	2094	3627	10076	74
H(8C)	2845	4771	9779	74
H(10A)	1812	1044	8332	81
H(10B)	2707	-84	8552	81
H(10C)	3377	1124	8274	81
H(11A)	1579	1385	9913	89
H(11B)	1575	82	9575	89
H(11C)	728	1211	9320	89
H(12A)	4759	1331	9224	91
H(12B)	4058	104	9464	91
H(12C)	4033	1340	9856	91
H(14A)	8565	2859	7881	43
H(14B)	8810	3791	7334	43
H(15A)	9402	2088	6724	75
H(15B)	9186	1174	7276	75
H(15C)	10353	2166	7291	75
H(17)	3377	3520	7578	34
H(19A)	1717	5000	6012	72
H(19B)	1177	5926	6511	72
H(19C)	402	4673	6370	72

Table 6. Torsion angles [°] for KYu10_Maimone.

C(8)-Si(1A)-O(1)-C(1)	-53.33(19)
C(7)-Si(1A)-O(1)-C(1)	67.05(19)
C(9)-Si(1A)-O(1)-C(1)	-173.20(16)
Si(1B)-O(1)-C(1)-C(2)	149.9(5)
Si(1A)-O(1)-C(1)-C(2)	106.13(17)
Si(1B)-O(1)-C(1)-C(6)	-99.4(5)
Si(1A)-O(1)-C(1)-C(6)	-143.15(14)
O(1)-C(1)-C(2)-C(3)	79.4(2)
C(6)-C(1)-C(2)-C(3)	-35.7(2)
C(1)-C(2)-C(3)-O(2)	-103.75(19)
C(1)-C(2)-C(3)-C(5)	15.8(2)
C(1)-C(2)-C(3)-C(4)	138.12(18)
O(2)-C(3)-C(5)-C(13)	-34.6(3)
C(4)-C(3)-C(5)-C(13)	86.1(3)
C(2)-C(3)-C(5)-C(13)	-154.9(2)
O(2)-C(3)-C(5)-C(6)	131.05(17)
C(4)-C(3)-C(5)-C(6)	-108.16(19)
C(2)-C(3)-C(5)-C(6)	10.8(2)
C(13)-C(5)-C(6)-C(17)	11.3(2)
C(3)-C(5)-C(6)-C(17)	-158.42(16)
C(13)-C(5)-C(6)-C(1)	136.98(18)
C(3)-C(5)-C(6)-C(1)	-32.7(2)
O(1)-C(1)-C(6)-C(5)	-75.39(18)
C(2)-C(1)-C(6)-C(5)	40.80(18)
O(1)-C(1)-C(6)-C(17)	40.2(2)
C(2)-C(1)-C(6)-C(17)	156.40(17)
O(1)-Si(1A)-C(9)-C(12)	63.6(2)
C(8)-Si(1A)-C(9)-C(12)	-57.2(2)
C(7)-Si(1A)-C(9)-C(12)	-179.8(2)
O(1)-Si(1A)-C(9)-C(11)	-176.58(18)
C(8)-Si(1A)-C(9)-C(11)	62.6(2)
C(7)-Si(1A)-C(9)-C(11)	-60.0(2)
O(1)-Si(1A)-C(9)-C(10)	-56.8(2)
C(8)-Si(1A)-C(9)-C(10)	-177.61(18)
C(7)-Si(1A)-C(9)-C(10)	59.8(2)
C(6)-C(5)-C(13)-O(3)	178.7(2)
C(3)-C(5)-C(13)-O(3)	-15.8(4)
C(6)-C(5)-C(13)-C(16)	-2.2(2)
C(3)-C(5)-C(13)-C(16)	163.3(2)
C(14)-O(3)-C(13)-C(5)	-19.6(3)

C(14)-O(3)-C(13)-C(16)	161.31(18)
C(13)-O(3)-C(14)-C(15)	-179.57(19)
C(5)-C(13)-C(16)-O(4)	173.6(2)
O(3)-C(13)-C(16)-O(4)	-7.1(3)
C(5)-C(13)-C(16)-C(17)	-7.9(2)
O(3)-C(13)-C(16)-C(17)	171.46(17)
C(5)-C(6)-C(17)-C(18)	-135.58(18)
C(1)-C(6)-C(17)-C(18)	110.6(2)
C(5)-C(6)-C(17)-C(16)	-14.87(19)
C(1)-C(6)-C(17)-C(16)	-128.70(18)
O(4)-C(16)-C(17)-C(18)	-42.5(3)
C(13)-C(16)-C(17)-C(18)	138.96(18)
O(4)-C(16)-C(17)-C(6)	-167.3(2)
C(13)-C(16)-C(17)-C(6)	14.1(2)
C(19)-O(6)-C(18)-O(5)	2.9(3)
C(19)-O(6)-C(18)-C(17)	-174.8(2)
C(6)-C(17)-C(18)-O(5)	43.3(3)
C(16)-C(17)-C(18)-O(5)	-73.6(3)
C(6)-C(17)-C(18)-O(6)	-138.99(19)
C(16)-C(17)-C(18)-O(6)	104.1(2)

Symmetry transformations used to generate equivalent atoms:

Bicyclic enol 8: (grown via slow evaporation from EtOAc/hexanes at 23 ºC)

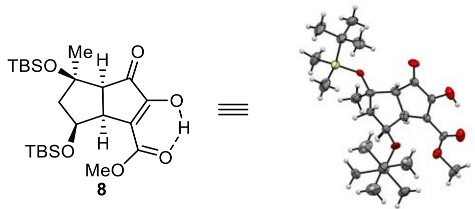

X-ray
[CCDC 2491906]

A colorless block 0.10 x 0.05 x 0.05 mm in size was mounted on a Cryoloop with Paratone oil. Data were collected in a nitrogen gas stream at 100(2) K using omega scans. Crystal-to-detector distance was 31.12 mm and exposure time was 2.50 seconds per frame at low angles and 10.00 seconds at high angles, using a scan width of 0.5°. Data collection was 100% complete to 74.000° in θ. A total of 29729 reflections were collected covering the indices -8<=h<=8, -12<=k<=14, -39<=l<=39. 5487 reflections were found to be symmetry independent, with an R_{int} of 0.0580. Indexing and unit cell refinement indicated a primitive, orthorhombic lattice. The space group was found to be P 21 21 21 (No. 19). The data were integrated using the CrysAlis^{Pro} 1.172.43.143a software program and scaled using the SCALE3 ABSPACK scaling algorithm. Solution by intrinsic phasing (SHELXT-2015) produced a heavy-atom phasing model consistent with the proposed structure. All non-hydrogen atoms were refined anisotropically by full-matrix least-squares (SHELXL-2014). All hydrogen atoms were placed using a riding model. Their positions were constrained relative to their parent atom using the appropriate HFIX command in SHELXL-2014.

Table 1. Crystal data and structure refinement for KYu11_Maimone.

Identification code	KYu11_Maimone	
Empirical formula	C23 H42 O6 Si2	
Formula weight	470.74	
Temperature	100(2) K	
Wavelength	1.54184 Å	
Crystal system	Orthorhombic	
Space group	P 21 21 21	
Unit cell dimensions	a = 7.1830(2) Å	α= 90°.
	b = 11.9682(3) Å	β= 90°.
	c = 31.3044(8) Å	γ = 90°.
Volume	2691.18(12) $Å^3$	
Z	4	
Density (calculated)	1.162 Mg/m^3	
Absorption coefficient	1.465 mm^{-1}	
F(000)	1024	
Crystal size	0.100 x 0.050 x 0.050 mm^3	
Theta range for data collection	3.954 to 74.497°.	
Index ranges	-8<=h<=8, -12<=k<=14, -39<=l<=39	
Reflections collected	29729	
Independent reflections	5487 [R(int) = 0.0580]	
Completeness to theta = 74.000°	100.0 %	
Absorption correction	Semi-empirical from equivalents	
Max. and min. transmission	1.00000 and 0.78044	
Refinement method	Full-matrix least-squares on F^2	
Data / restraints / parameters	5487 / 235 / 538	
Goodness-of-fit on F^2	1.029	
Final R indices [I>2sigma(I)]	R1 = 0.0351, wR2 = 0.0792	
R indices (all data)	R1 = 0.0416, wR2 = 0.0819	
Absolute structure parameter	-0.006(13)	
Extinction coefficient	n/a	
Largest diff. peak and hole	0.222 and -0.165 e.$Å^{-3}$	

Table 2. Atomic coordinates (x 10^4) and equivalent isotropic displacement parameters ($Å^2$x 10^3) for KYu11_Maimone. U(eq) is defined as one third of the trace of the orthogonalized U^{ij} tensor.

	x	y	z	U(eq)
Si(1A)	6763(11)	6011(4)	5909(2)	35(1)
Si(1B)	6191(10)	6074(4)	5849(2)	34(1)
Si(2A)	2551(2)	1636(1)	7024(1)	35(1)
Si(2B)	1499(2)	2243(1)	6934(1)	32(1)
O(1A)	5161(18)	5154(10)	5765(5)	29(2)
O(1B)	4831(19)	4931(10)	5756(5)	33(2)
O(2A)	2536(13)	1839(9)	6505(4)	29(2)
O(2B)	2120(14)	1987(12)	6443(4)	40(3)
O(3A)	-423(10)	2122(8)	5878(3)	44(2)
O(3B)	-439(11)	2504(10)	5761(3)	45(2)
O(4A)	-803(10)	3848(9)	5259(2)	47(2)
O(4B)	-321(12)	4282(8)	5147(3)	46(2)
O(5A)	1490(14)	5068(7)	4746(2)	49(2)
O(5B)	2368(17)	5327(6)	4703(2)	47(2)
O(6A)	4534(15)	4639(6)	4765(2)	37(2)
O(6B)	5332(18)	4732(6)	4787(2)	37(2)
C(1)	5170(3)	3870(2)	5835(1)	32(1)
C(2)	4246(3)	3515(2)	6252(1)	34(1)
C(3)	3726(3)	2287(2)	6187(1)	36(1)
C(4)	5403(4)	1517(2)	6211(1)	52(1)
C(5)	2972(3)	2300(2)	5726(1)	34(1)
C(6)	4158(3)	3176(2)	5484(1)	33(1)
C(7A)	7775(18)	6656(6)	5420(3)	45(2)
C(7B)	6776(17)	6720(6)	5324(2)	46(2)
C(8A)	8635(15)	5343(10)	6240(3)	41(2)
C(8B)	8330(14)	5618(9)	6125(3)	42(2)
C(9A)	5433(18)	7136(13)	6217(5)	43(3)
C(9B)	4818(18)	7082(12)	6196(5)	47(3)
C(10A)	4570(40)	6470(20)	6637(7)	58(4)
C(10B)	4300(40)	6630(20)	6606(7)	54(4)
C(11A)	3884(19)	7685(8)	5946(3)	59(3)
C(11B)	3078(16)	7355(10)	5960(3)	67(2)
C(12A)	6820(20)	8039(11)	6358(4)	60(3)
C(12B)	6015(19)	8116(10)	6288(4)	61(3)
C(13A)	3480(30)	2845(17)	7270(5)	62(5)
C(13B)	3340(20)	3011(19)	7305(6)	50(4)
C(14A)	3869(12)	341(6)	7169(2)	56(2)

C(14B)	-653(10)	3086(6)	6913(3)	56(2)
C(15A)	35(7)	1459(5)	7172(2)	46(1)
C(15B)	1092(7)	821(4)	7176(1)	33(1)
C(16A)	-793(10)	435(7)	6934(3)	56(2)
C(16B)	-61(11)	78(7)	6876(3)	44(2)
C(17A)	-1066(8)	2521(6)	7040(2)	54(1)
C(17B)	2947(11)	237(8)	7268(3)	52(2)
C(18A)	-200(30)	1289(15)	7658(7)	73(4)
C(18B)	50(30)	951(14)	7604(7)	48(3)
C(19A)	938(15)	2556(10)	5707(4)	28(2)
C(19B)	997(17)	2856(11)	5616(4)	30(2)
C(20A)	844(13)	3461(9)	5393(3)	30(2)
C(20B)	1227(17)	3773(9)	5307(3)	29(2)
C(21A)	2541(17)	3761(11)	5256(4)	31(2)
C(21B)	3016(18)	3946(10)	5213(4)	26(2)
C(22A)	2794(16)	4549(8)	4918(3)	37(2)
C(22B)	3510(20)	4723(7)	4872(3)	28(2)
C(23A)	4812(18)	5285(7)	4383(2)	53(2)
C(23B)	5840(20)	5431(7)	4425(3)	53(3)

Table 3. Bond lengths [Å] and angles [°] for KYu11_Maimone.

Si(1A)-O(1A)	1.607(10)
Si(1A)-C(7A)	1.864(8)
Si(1A)-C(8A)	1.876(9)
Si(1A)-C(9A)	1.911(15)
Si(1B)-O(1B)	1.706(9)
Si(1B)-C(8B)	1.845(9)
Si(1B)-C(7B)	1.867(8)
Si(1B)-C(9B)	1.898(15)
Si(2A)-O(2A)	1.643(12)
Si(2A)-C(13A)	1.770(19)
Si(2A)-C(14A)	1.871(7)
Si(2A)-C(15A)	1.878(5)
Si(2B)-O(2B)	1.631(14)
Si(2B)-C(14B)	1.847(7)
Si(2B)-C(15B)	1.885(5)
Si(2B)-C(13B)	1.983(16)
O(1A)-C(1)	1.552(12)
O(1B)-C(1)	1.316(13)
O(2A)-C(3)	1.418(13)
O(2B)-C(3)	1.450(13)
O(3A)-C(19A)	1.229(11)
O(3B)-C(19B)	1.204(13)
O(4A)-C(20A)	1.338(9)
O(4A)-H(4A)	0.84(3)
O(4B)-C(20B)	1.363(12)
O(4B)-H(4B)	0.82(11)
O(5A)-C(22A)	1.246(9)
O(5B)-C(22B)	1.216(11)
O(6A)-C(22A)	1.342(7)
O(6A)-C(23A)	1.439(9)
O(6B)-C(22B)	1.335(10)
O(6B)-C(23B)	1.454(10)
C(1)-C(2)	1.522(3)
C(1)-C(6)	1.560(3)
C(1)-H(1A)	1.0000
C(1)-H(1B)	1.0000
C(2)-C(3)	1.530(3)
C(2)-H(2A)	0.9900
C(2)-H(2B)	0.9900
C(3)-C(4)	1.519(4)

C(3)-C(5)	1.542(3)
C(4)-H(4C)	0.9800
C(4)-H(4D)	0.9800
C(4)-H(4E)	0.9800
C(5)-C(19A)	1.494(11)
C(5)-C(6)	1.549(3)
C(5)-C(19B)	1.604(13)
C(5)-H(5A)	1.0000
C(5)-H(5B)	1.0000
C(6)-C(21B)	1.496(13)
C(6)-C(21A)	1.532(14)
C(6)-H(6A)	1.0000
C(6)-H(6B)	1.0000
C(7A)-H(7AA)	0.9800
C(7A)-H(7AB)	0.9800
C(7A)-H(7AC)	0.9800
C(7B)-H(7BA)	0.9800
C(7B)-H(7BB)	0.9800
C(7B)-H(7BC)	0.9800
C(8A)-H(8AA)	0.9800
C(8A)-H(8AB)	0.9800
C(8A)-H(8AC)	0.9800
C(8B)-H(8BA)	0.9800
C(8B)-H(8BB)	0.9800
C(8B)-H(8BC)	0.9800
C(9A)-C(12A)	1.536(15)
C(9A)-C(11A)	1.546(15)
C(9A)-C(10A)	1.66(3)
C(9B)-C(10B)	1.44(2)
C(9B)-C(11B)	1.487(15)
C(9B)-C(12B)	1.535(14)
C(10A)-H(10A)	0.9800
C(10A)-H(10B)	0.9800
C(10A)-H(10C)	0.9800
C(10B)-H(10D)	0.9800
C(10B)-H(10E)	0.9800
C(10B)-H(10F)	0.9800
C(11A)-H(11A)	0.9800
C(11A)-H(11B)	0.9800
C(11A)-H(11C)	0.9800
C(11B)-H(11D)	0.9800
C(11B)-H(11E)	0.9800

C(11B)-H(11F)	0.9800
C(12A)-H(12A)	0.9800
C(12A)-H(12B)	0.9800
C(12A)-H(12C)	0.9800
C(12B)-H(12D)	0.9800
C(12B)-H(12E)	0.9800
C(12B)-H(12F)	0.9800
C(13A)-H(13A)	0.9800
C(13A)-H(13B)	0.9800
C(13A)-H(13C)	0.9800
C(13B)-H(13D)	0.9800
C(13B)-H(13E)	0.9800
C(13B)-H(13F)	0.9800
C(14A)-H(14A)	0.9800
C(14A)-H(14B)	0.9800
C(14A)-H(14C)	0.9800
C(14B)-H(14D)	0.9800
C(14B)-H(14E)	0.9800
C(14B)-H(14F)	0.9800
C(15A)-C(18A)	1.54(2)
C(15A)-C(16A)	1.553(10)
C(15A)-C(17A)	1.554(9)
C(15B)-C(17B)	1.532(9)
C(15B)-C(16B)	1.536(9)
C(15B)-C(18B)	1.544(19)
C(16A)-H(16A)	0.9800
C(16A)-H(16B)	0.9800
C(16A)-H(16C)	0.9800
C(16B)-H(16D)	0.9800
C(16B)-H(16E)	0.9800
C(16B)-H(16F)	0.9800
C(17A)-H(17A)	0.9800
C(17A)-H(17B)	0.9800
C(17A)-H(17C)	0.9800
C(17B)-H(17D)	0.9800
C(17B)-H(17E)	0.9800
C(17B)-H(17F)	0.9800
C(18A)-H(18A)	0.9800
C(18A)-H(18B)	0.9800
C(18A)-H(18C)	0.9800
C(18B)-H(18D)	0.9800
C(18B)-H(18E)	0.9800

C(18B)-H(18F)	0.9800
C(19A)-C(20A)	1.464(9)
C(19B)-C(20B)	1.473(10)
C(20A)-C(21A)	1.341(9)
C(20B)-C(21B)	1.334(11)
C(21A)-C(22A)	1.430(12)
C(21B)-C(22B)	1.460(12)
C(23A)-H(23A)	0.9800
C(23A)-H(23B)	0.9800
C(23A)-H(23C)	0.9800
C(23B)-H(23D)	0.9800
C(23B)-H(23E)	0.9800
C(23B)-H(23F)	0.9800

O(1A)-Si(1A)-C(7A)	108.2(6)
O(1A)-Si(1A)-C(8A)	113.4(7)
C(7A)-Si(1A)-C(8A)	110.5(5)
O(1A)-Si(1A)-C(9A)	103.5(7)
C(7A)-Si(1A)-C(9A)	108.5(6)
C(8A)-Si(1A)-C(9A)	112.3(6)
O(1B)-Si(1B)-C(8B)	108.7(6)
O(1B)-Si(1B)-C(7B)	108.1(6)
C(8B)-Si(1B)-C(7B)	110.4(4)
O(1B)-Si(1B)-C(9B)	108.0(7)
C(8B)-Si(1B)-C(9B)	110.7(5)
C(7B)-Si(1B)-C(9B)	110.9(6)
O(2A)-Si(2A)-C(13A)	108.1(7)
O(2A)-Si(2A)-C(14A)	111.4(4)
C(13A)-Si(2A)-C(14A)	112.4(7)
O(2A)-Si(2A)-C(15A)	104.7(4)
C(13A)-Si(2A)-C(15A)	110.4(7)
C(14A)-Si(2A)-C(15A)	109.5(3)
O(2B)-Si(2B)-C(14B)	107.3(4)
O(2B)-Si(2B)-C(15B)	104.5(5)
C(14B)-Si(2B)-C(15B)	112.2(3)
O(2B)-Si(2B)-C(13B)	117.2(7)
C(14B)-Si(2B)-C(13B)	109.0(8)
C(15B)-Si(2B)-C(13B)	106.7(6)
C(1)-O(1A)-Si(1A)	126.1(10)
C(1)-O(1B)-Si(1B)	129.4(10)
C(3)-O(2A)-Si(2A)	138.3(6)
C(3)-O(2B)-Si(2B)	133.8(9)

C(20A)-O(4A)-H(4A)	111(10)
C(20B)-O(4B)-H(4B)	104(7)
C(22A)-O(6A)-C(23A)	117.9(7)
C(22B)-O(6B)-C(23B)	113.8(7)
O(1B)-C(1)-C(2)	110.5(7)
C(2)-C(1)-O(1A)	113.4(6)
O(1B)-C(1)-C(6)	107.2(6)
C(2)-C(1)-C(6)	104.62(17)
O(1A)-C(1)-C(6)	115.1(6)
C(2)-C(1)-H(1A)	107.8
O(1A)-C(1)-H(1A)	107.8
C(6)-C(1)-H(1A)	107.8
O(1B)-C(1)-H(1B)	111.4
C(2)-C(1)-H(1B)	111.4
C(6)-C(1)-H(1B)	111.4
C(1)-C(2)-C(3)	105.18(18)
C(1)-C(2)-H(2A)	110.7
C(3)-C(2)-H(2A)	110.7
C(1)-C(2)-H(2B)	110.7
C(3)-C(2)-H(2B)	110.7
H(2A)-C(2)-H(2B)	108.8
O(2A)-C(3)-C(4)	102.3(4)
O(2B)-C(3)-C(4)	116.9(5)
O(2A)-C(3)-C(2)	114.7(5)
O(2B)-C(3)-C(2)	111.1(6)
C(4)-C(3)-C(2)	112.5(2)
O(2A)-C(3)-C(5)	116.7(4)
O(2B)-C(3)-C(5)	103.9(4)
C(4)-C(3)-C(5)	109.4(2)
C(2)-C(3)-C(5)	101.49(19)
C(3)-C(4)-H(4C)	109.5
C(3)-C(4)-H(4D)	109.5
H(4C)-C(4)-H(4D)	109.5
C(3)-C(4)-H(4E)	109.5
H(4C)-C(4)-H(4E)	109.5
H(4D)-C(4)-H(4E)	109.5
C(19A)-C(5)-C(3)	112.5(5)
C(19A)-C(5)-C(6)	112.4(5)
C(3)-C(5)-C(6)	105.81(18)
C(3)-C(5)-C(19B)	121.0(5)
C(6)-C(5)-C(19B)	95.8(5)
C(19A)-C(5)-H(5A)	108.7

C(3)-C(5)-H(5A)	108.7
C(6)-C(5)-H(5A)	108.7
C(3)-C(5)-H(5B)	110.9
C(6)-C(5)-H(5B)	110.9
C(19B)-C(5)-H(5B)	110.9
C(21B)-C(6)-C(5)	113.1(5)
C(21A)-C(6)-C(5)	96.9(5)
C(21B)-C(6)-C(1)	109.1(5)
C(21A)-C(6)-C(1)	115.9(5)
C(5)-C(6)-C(1)	105.71(18)
C(21A)-C(6)-H(6A)	112.4
C(5)-C(6)-H(6A)	112.4
C(1)-C(6)-H(6A)	112.4
C(21B)-C(6)-H(6B)	109.6
C(5)-C(6)-H(6B)	109.6
C(1)-C(6)-H(6B)	109.6
Si(1A)-C(7A)-H(7AA)	109.5
Si(1A)-C(7A)-H(7AB)	109.5
H(7AA)-C(7A)-H(7AB)	109.5
Si(1A)-C(7A)-H(7AC)	109.5
H(7AA)-C(7A)-H(7AC)	109.5
H(7AB)-C(7A)-H(7AC)	109.5
Si(1B)-C(7B)-H(7BA)	109.5
Si(1B)-C(7B)-H(7BB)	109.5
H(7BA)-C(7B)-H(7BB)	109.5
Si(1B)-C(7B)-H(7BC)	109.5
H(7BA)-C(7B)-H(7BC)	109.5
H(7BB)-C(7B)-H(7BC)	109.5
Si(1A)-C(8A)-H(8AA)	109.5
Si(1A)-C(8A)-H(8AB)	109.5
H(8AA)-C(8A)-H(8AB)	109.5
Si(1A)-C(8A)-H(8AC)	109.5
H(8AA)-C(8A)-H(8AC)	109.5
H(8AB)-C(8A)-H(8AC)	109.5
Si(1B)-C(8B)-H(8BA)	109.5
Si(1B)-C(8B)-H(8BB)	109.5
H(8BA)-C(8B)-H(8BB)	109.5
Si(1B)-C(8B)-H(8BC)	109.5
H(8BA)-C(8B)-H(8BC)	109.5
H(8BB)-C(8B)-H(8BC)	109.5
C(12A)-C(9A)-C(11A)	109.0(10)
C(12A)-C(9A)-C(10A)	110.6(15)

C(11A)-C(9A)-C(10A)	111.7(13)
C(12A)-C(9A)-Si(1A)	108.5(7)
C(11A)-C(9A)-Si(1A)	112.5(9)
C(10A)-C(9A)-Si(1A)	104.4(12)
C(10B)-C(9B)-C(11B)	108.0(14)
C(10B)-C(9B)-C(12B)	106.1(16)
C(11B)-C(9B)-C(12B)	112.7(11)
C(10B)-C(9B)-Si(1B)	113.8(13)
C(11B)-C(9B)-Si(1B)	107.1(9)
C(12B)-C(9B)-Si(1B)	109.2(7)
C(9A)-C(10A)-H(10A)	109.5
C(9A)-C(10A)-H(10B)	109.5
H(10A)-C(10A)-H(10B)	109.5
C(9A)-C(10A)-H(10C)	109.5
H(10A)-C(10A)-H(10C)	109.5
H(10B)-C(10A)-H(10C)	109.5
C(9B)-C(10B)-H(10D)	109.5
C(9B)-C(10B)-H(10E)	109.5
H(10D)-C(10B)-H(10E)	109.5
C(9B)-C(10B)-H(10F)	109.5
H(10D)-C(10B)-H(10F)	109.5
H(10E)-C(10B)-H(10F)	109.5
C(9A)-C(11A)-H(11A)	109.5
C(9A)-C(11A)-H(11B)	109.5
H(11A)-C(11A)-H(11B)	109.5
C(9A)-C(11A)-H(11C)	109.5
H(11A)-C(11A)-H(11C)	109.5
H(11B)-C(11A)-H(11C)	109.5
C(9B)-C(11B)-H(11D)	109.5
C(9B)-C(11B)-H(11E)	109.5
H(11D)-C(11B)-H(11E)	109.5
C(9B)-C(11B)-H(11F)	109.5
H(11D)-C(11B)-H(11F)	109.5
H(11E)-C(11B)-H(11F)	109.5
C(9A)-C(12A)-H(12A)	109.5
C(9A)-C(12A)-H(12B)	109.5
H(12A)-C(12A)-H(12B)	109.5
C(9A)-C(12A)-H(12C)	109.5
H(12A)-C(12A)-H(12C)	109.5
H(12B)-C(12A)-H(12C)	109.5
C(9B)-C(12B)-H(12D)	109.5
C(9B)-C(12B)-H(12E)	109.5

H(12D)-C(12B)-H(12E)	109.5
C(9B)-C(12B)-H(12F)	109.5
H(12D)-C(12B)-H(12F)	109.5
H(12E)-C(12B)-H(12F)	109.5
Si(2A)-C(13A)-H(13A)	109.5
Si(2A)-C(13A)-H(13B)	109.5
H(13A)-C(13A)-H(13B)	109.5
Si(2A)-C(13A)-H(13C)	109.5
H(13A)-C(13A)-H(13C)	109.5
H(13B)-C(13A)-H(13C)	109.5
Si(2B)-C(13B)-H(13D)	109.5
Si(2B)-C(13B)-H(13E)	109.5
H(13D)-C(13B)-H(13E)	109.5
Si(2B)-C(13B)-H(13F)	109.5
H(13D)-C(13B)-H(13F)	109.5
H(13E)-C(13B)-H(13F)	109.5
Si(2A)-C(14A)-H(14A)	109.5
Si(2A)-C(14A)-H(14B)	109.5
H(14A)-C(14A)-H(14B)	109.5
Si(2A)-C(14A)-H(14C)	109.5
H(14A)-C(14A)-H(14C)	109.5
H(14B)-C(14A)-H(14C)	109.5
Si(2B)-C(14B)-H(14D)	109.5
Si(2B)-C(14B)-H(14E)	109.5
H(14D)-C(14B)-H(14E)	109.5
Si(2B)-C(14B)-H(14F)	109.5
H(14D)-C(14B)-H(14F)	109.5
H(14E)-C(14B)-H(14F)	109.5
C(18A)-C(15A)-C(16A)	109.1(9)
C(18A)-C(15A)-C(17A)	108.3(7)
C(16A)-C(15A)-C(17A)	108.8(5)
C(18A)-C(15A)-Si(2A)	111.4(9)
C(16A)-C(15A)-Si(2A)	109.8(4)
C(17A)-C(15A)-Si(2A)	109.4(4)
C(17B)-C(15B)-C(16B)	108.7(6)
C(17B)-C(15B)-C(18B)	107.7(9)
C(16B)-C(15B)-C(18B)	109.2(9)
C(17B)-C(15B)-Si(2B)	110.6(4)
C(16B)-C(15B)-Si(2B)	111.1(4)
C(18B)-C(15B)-Si(2B)	109.4(7)
C(15A)-C(16A)-H(16A)	109.5
C(15A)-C(16A)-H(16B)	109.5

H(16A)-C(16A)-H(16B)	109.5
C(15A)-C(16A)-H(16C)	109.5
H(16A)-C(16A)-H(16C)	109.5
H(16B)-C(16A)-H(16C)	109.5
C(15B)-C(16B)-H(16D)	109.5
C(15B)-C(16B)-H(16E)	109.5
H(16D)-C(16B)-H(16E)	109.5
C(15B)-C(16B)-H(16F)	109.5
H(16D)-C(16B)-H(16F)	109.5
H(16E)-C(16B)-H(16F)	109.5
C(15A)-C(17A)-H(17A)	109.5
C(15A)-C(17A)-H(17B)	109.5
H(17A)-C(17A)-H(17B)	109.5
C(15A)-C(17A)-H(17C)	109.5
H(17A)-C(17A)-H(17C)	109.5
H(17B)-C(17A)-H(17C)	109.5
C(15B)-C(17B)-H(17D)	109.5
C(15B)-C(17B)-H(17E)	109.5
H(17D)-C(17B)-H(17E)	109.5
C(15B)-C(17B)-H(17F)	109.5
H(17D)-C(17B)-H(17F)	109.5
H(17E)-C(17B)-H(17F)	109.5
C(15A)-C(18A)-H(18A)	109.5
C(15A)-C(18A)-H(18B)	109.5
H(18A)-C(18A)-H(18B)	109.5
C(15A)-C(18A)-H(18C)	109.5
H(18A)-C(18A)-H(18C)	109.5
H(18B)-C(18A)-H(18C)	109.5
C(15B)-C(18B)-H(18D)	109.5
C(15B)-C(18B)-H(18E)	109.5
H(18D)-C(18B)-H(18E)	109.5
C(15B)-C(18B)-H(18F)	109.5
H(18D)-C(18B)-H(18F)	109.5
H(18E)-C(18B)-H(18F)	109.5
O(3A)-C(19A)-C(20A)	124.6(9)
O(3A)-C(19A)-C(5)	132.4(7)
C(20A)-C(19A)-C(5)	102.9(6)
O(3B)-C(19B)-C(20B)	127.3(10)
O(3B)-C(19B)-C(5)	122.1(8)
C(20B)-C(19B)-C(5)	110.5(8)
O(4A)-C(20A)-C(21A)	127.6(8)
O(4A)-C(20A)-C(19A)	120.5(7)

C(21A)-C(20A)-C(19A)	111.8(8)
C(21B)-C(20B)-O(4B)	129.5(9)
C(21B)-C(20B)-C(19B)	111.6(9)
O(4B)-C(20B)-C(19B)	118.8(9)
C(20A)-C(21A)-C(22A)	121.9(10)
C(20A)-C(21A)-C(6)	114.7(8)
C(22A)-C(21A)-C(6)	123.3(7)
C(20B)-C(21B)-C(22B)	119.6(10)
C(20B)-C(21B)-C(6)	107.9(8)
C(22B)-C(21B)-C(6)	132.3(9)
O(5A)-C(22A)-O(6A)	120.4(8)
O(5A)-C(22A)-C(21A)	123.6(8)
O(6A)-C(22A)-C(21A)	115.8(8)
O(5B)-C(22B)-O(6B)	124.8(8)
O(5B)-C(22B)-C(21B)	122.2(10)
O(6B)-C(22B)-C(21B)	112.9(8)
O(6A)-C(23A)-H(23A)	109.5
O(6A)-C(23A)-H(23B)	109.5
H(23A)-C(23A)-H(23B)	109.5
O(6A)-C(23A)-H(23C)	109.5
H(23A)-C(23A)-H(23C)	109.5
H(23B)-C(23A)-H(23C)	109.5
O(6B)-C(23B)-H(23D)	109.5
O(6B)-C(23B)-H(23E)	109.5
H(23D)-C(23B)-H(23E)	109.5
O(6B)-C(23B)-H(23F)	109.5
H(23D)-C(23B)-H(23F)	109.5
H(23E)-C(23B)-H(23F)	109.5

Symmetry transformations used to generate equivalent atoms:

Table 4. Anisotropic displacement parameters ($Å^2$x 10^3) for KYu11_Maimone. The anisotropic displacement factor exponent takes the form: $-2\pi^2[h^2a^{*2}U^{11} + ... + 2 h k a^* b^* U^{12}]$

	U^{11}	U^{22}	U^{33}	U^{23}	U^{13}	U^{12}
Si(1A)	42(2)	35(1)	28(2)	-5(1)	4(1)	-3(1)
Si(1B)	43(2)	32(1)	28(1)	4(1)	0(1)	-5(1)
Si(2A)	34(1)	40(1)	31(1)	7(1)	2(1)	3(1)
Si(2B)	28(1)	42(1)	26(1)	6(1)	-3(1)	-6(1)
O(1A)	44(4)	10(4)	32(2)	0(3)	-2(3)	-12(3)
O(1B)	54(4)	15(4)	29(2)	2(3)	-2(3)	-20(2)
O(2A)	18(3)	36(2)	34(4)	9(2)	-6(2)	-7(2)
O(2B)	24(5)	78(7)	18(3)	4(3)	0(3)	-23(4)
O(3A)	28(2)	64(4)	38(3)	4(2)	3(2)	-13(2)
O(3B)	24(2)	68(5)	43(4)	0(3)	7(2)	-14(3)
O(4A)	37(3)	69(4)	34(3)	-6(3)	-9(2)	17(3)
O(4B)	39(3)	50(4)	47(4)	-6(3)	-14(3)	16(3)
O(5A)	50(4)	62(3)	35(2)	2(2)	-8(2)	22(3)
O(5B)	56(5)	42(3)	43(3)	3(2)	-11(3)	14(3)
O(6A)	36(4)	47(2)	28(2)	9(2)	1(3)	5(3)
O(6B)	44(5)	39(2)	28(2)	4(2)	4(3)	4(3)
C(1)	31(1)	33(1)	31(1)	6(1)	0(1)	-2(1)
C(2)	29(1)	47(1)	27(1)	2(1)	-4(1)	-10(1)
C(3)	29(1)	48(1)	32(1)	3(1)	5(1)	-13(1)
C(4)	48(1)	50(2)	58(2)	20(1)	-2(1)	-4(1)
C(5)	25(1)	44(1)	32(1)	-8(1)	5(1)	-3(1)
C(6)	32(1)	37(1)	30(1)	-1(1)	4(1)	7(1)
C(7A)	64(6)	30(3)	41(3)	-1(2)	9(3)	-9(3)
C(7B)	65(5)	37(3)	36(3)	-1(2)	9(3)	-13(4)
C(8A)	36(4)	47(5)	40(5)	1(3)	-11(3)	-3(3)
C(8B)	40(4)	42(4)	43(5)	-4(3)	-6(3)	-4(3)
C(9A)	57(6)	41(4)	29(3)	0(3)	7(4)	3(4)
C(9B)	63(6)	38(4)	39(4)	-3(3)	14(4)	-10(4)
C(10A)	83(8)	56(10)	35(6)	-10(5)	33(6)	8(7)
C(10B)	88(9)	34(5)	39(5)	3(3)	9(4)	9(5)
C(11A)	81(6)	51(4)	46(4)	-5(3)	0(4)	6(4)
C(11B)	71(5)	73(6)	59(4)	3(4)	15(4)	5(4)
C(12A)	80(7)	42(4)	57(5)	-21(3)	14(5)	-9(5)
C(12B)	87(7)	33(3)	62(6)	-8(3)	28(5)	-8(5)
C(13A)	92(9)	69(8)	23(4)	-3(6)	9(5)	-4(6)
C(13B)	46(5)	62(7)	40(6)	-28(4)	14(4)	-32(6)
C(14A)	68(5)	53(4)	47(4)	20(3)	4(3)	18(4)

C(14B)	43(4)	52(4)	73(5)	8(3)	-11(3)	-1(3)
C(15A)	42(3)	61(3)	35(3)	11(2)	8(2)	-2(3)
C(15B)	33(3)	45(3)	20(2)	8(2)	5(2)	-3(2)
C(16A)	44(4)	60(5)	64(5)	24(4)	6(3)	-9(3)
C(16B)	44(4)	48(4)	39(3)	8(3)	-4(3)	-10(3)
C(17A)	32(3)	75(4)	54(3)	-1(3)	8(2)	10(3)
C(17B)	58(5)	60(4)	39(4)	16(3)	-4(4)	10(4)
C(18A)	59(5)	113(12)	46(8)	32(9)	20(5)	7(9)
C(18B)	53(7)	60(6)	32(5)	6(4)	17(4)	-1(5)
C(19A)	23(3)	40(5)	20(4)	-6(3)	0(2)	-4(3)
C(19B)	29(3)	40(6)	22(5)	-8(3)	3(3)	-4(3)
C(20A)	25(3)	44(4)	21(4)	-2(3)	1(2)	1(3)
C(20B)	33(5)	37(4)	19(3)	-4(3)	1(3)	-1(3)
C(21A)	26(5)	42(4)	24(3)	-2(3)	5(3)	-1(3)
C(21B)	25(5)	31(4)	21(4)	-5(3)	0(3)	1(3)
C(22A)	39(4)	44(4)	26(3)	-3(2)	11(3)	5(3)
C(22B)	39(6)	29(3)	18(3)	3(2)	7(3)	4(3)
C(23A)	64(6)	58(4)	36(3)	13(3)	7(3)	4(4)
C(23B)	77(7)	46(4)	35(3)	16(3)	16(4)	7(4)

Table 5. Hydrogen coordinates (x 10^4) and isotropic displacement parameters ($Å^2$x 10^3) for KYu11_Maimone.

	x	y	z	U(eq)
H(1A)	6497	3619	5845	38
H(1B)	6537	3713	5839	38
H(2A)	3123	3971	6308	41
H(2B)	5117	3600	6495	41
H(4C)	5941	1551	6499	78
H(4D)	6336	1753	6002	78
H(4E)	5013	749	6149	78
H(5A)	3189	1550	5595	40
H(5B)	3088	1546	5591	40
H(6A)	5048	2823	5278	39
H(6B)	5100	2786	5302	39
H(7AA)	8219	6067	5228	67
H(7AB)	8818	7140	5500	67
H(7AC)	6820	7101	5274	67
H(7BA)	7411	6169	5144	69
H(7BB)	7593	7365	5369	69
H(7BC)	5629	6965	5182	69
H(8AA)	8065	4915	6473	62
H(8AB)	9443	5924	6360	62
H(8AC)	9376	4841	6060	62
H(8BA)	8967	5054	5952	62
H(8BB)	8004	5297	6404	62
H(8BC)	9154	6261	6167	62
H(10A)	3747	5868	6541	87
H(10B)	3858	6996	6814	87
H(10C)	5589	6155	6807	87
H(10D)	3516	5972	6565	80
H(10E)	3618	7197	6769	80
H(10F)	5430	6419	6763	80
H(11A)	4447	8089	5706	89
H(11B)	3178	8209	6124	89
H(11C)	3046	7106	5836	89
H(11D)	3392	7677	5682	101
H(11E)	2349	7897	6126	101
H(11F)	2345	6673	5919	101
H(12A)	7806	7697	6531	90
H(12B)	6170	8604	6528	90

H(12C)	7372	8392	6106	90
H(12D)	7149	7890	6439	91
H(12E)	5308	8640	6466	91
H(12F)	6350	8480	6018	91
H(13A)	4825	2881	7216	92
H(13B)	3258	2812	7578	92
H(13C)	2881	3511	7151	92
H(13D)	3382	3806	7231	74
H(13E)	4568	2677	7263	74
H(13F)	2965	2929	7605	74
H(14A)	3452	-279	6988	84
H(14B)	3642	158	7469	84
H(14C)	5203	467	7124	84
H(14D)	-1579	2704	6735	84
H(14E)	-375	3820	6789	84
H(14F)	-1147	3182	7202	84
H(16A)	-697	554	6625	84
H(16B)	-2105	346	7013	84
H(16C)	-101	-239	7013	84
H(16D)	-1284	421	6829	66
H(16E)	-224	-661	7005	66
H(16F)	587	2	6602	66
H(17A)	-544	3174	7186	80
H(17B)	-2377	2434	7120	80
H(17C)	-971	2626	6730	80
H(17D)	3609	107	6999	79
H(17E)	2711	-479	7410	79
H(17F)	3708	711	7454	79
H(18A)	562	657	7751	109
H(18B)	-1514	1136	7722	109
H(18C)	190	1967	7808	109
H(18D)	723	1479	7787	72
H(18E)	-27	224	7747	72
H(18F)	-1209	1234	7551	72
H(23A)	4013	4995	4155	79
H(23B)	6119	5233	4295	79
H(23C)	4496	6068	4439	79
H(23D)	5284	5124	4164	79
H(23E)	7195	5448	4396	79
H(23F)	5372	6191	4472	79
H(4B)	100(140)	4750(90)	4980(40)	90(40)
H(4A)	-660(190)	4440(70)	5110(40)	129

Table 6. Torsion angles [°] for KYu11_Maimone.

C(7A)-Si(1A)-O(1A)-C(1)	116.5(11)
C(8A)-Si(1A)-O(1A)-C(1)	-6.5(14)
C(9A)-Si(1A)-O(1A)-C(1)	-128.5(12)
C(8B)-Si(1B)-O(1B)-C(1)	-3.3(15)
C(7B)-Si(1B)-O(1B)-C(1)	116.4(12)
C(9B)-Si(1B)-O(1B)-C(1)	-123.5(13)
C(13A)-Si(2A)-O(2A)-C(3)	42.8(14)
C(14A)-Si(2A)-O(2A)-C(3)	-81.2(12)
C(15A)-Si(2A)-O(2A)-C(3)	160.6(10)
C(14B)-Si(2B)-O(2B)-C(3)	116.2(10)
C(15B)-Si(2B)-O(2B)-C(3)	-124.5(10)
C(13B)-Si(2B)-O(2B)-C(3)	-6.7(15)
Si(1B)-O(1B)-C(1)-C(2)	98.3(12)
Si(1B)-O(1B)-C(1)-C(6)	-148.3(11)
Si(1A)-O(1A)-C(1)-C(2)	91.2(11)
Si(1A)-O(1A)-C(1)-C(6)	-148.4(9)
O(1B)-C(1)-C(2)-C(3)	147.8(6)
O(1A)-C(1)-C(2)-C(3)	159.0(6)
C(6)-C(1)-C(2)-C(3)	32.8(2)
Si(2A)-O(2A)-C(3)-C(4)	62.1(11)
Si(2A)-O(2A)-C(3)-C(2)	-60.1(11)
Si(2A)-O(2A)-C(3)-C(5)	-178.6(8)
Si(2B)-O(2B)-C(3)-C(4)	87.4(12)
Si(2B)-O(2B)-C(3)-C(2)	-43.6(11)
Si(2B)-O(2B)-C(3)-C(5)	-152.0(9)
C(1)-C(2)-C(3)-O(2A)	-168.7(4)
C(1)-C(2)-C(3)-O(2B)	-151.9(4)
C(1)-C(2)-C(3)-C(4)	74.8(2)
C(1)-C(2)-C(3)-C(5)	-42.0(2)
O(2A)-C(3)-C(5)-C(19A)	37.2(7)
C(4)-C(3)-C(5)-C(19A)	152.7(5)
C(2)-C(3)-C(5)-C(19A)	-88.2(5)
O(2A)-C(3)-C(5)-C(6)	160.3(5)
O(2B)-C(3)-C(5)-C(6)	150.2(6)
C(4)-C(3)-C(5)-C(6)	-84.2(2)
C(2)-C(3)-C(5)-C(6)	34.8(2)
O(2B)-C(3)-C(5)-C(19B)	43.2(8)
C(4)-C(3)-C(5)-C(19B)	168.7(6)
C(2)-C(3)-C(5)-C(19B)	-72.2(6)
C(3)-C(5)-C(6)-C(21B)	-134.6(6)

C(19B)-C(5)-C(6)-C(21B)	-10.1(7)
C(19A)-C(5)-C(6)-C(21A)	-11.7(7)
C(3)-C(5)-C(6)-C(21A)	-134.8(5)
C(19A)-C(5)-C(6)-C(1)	107.7(5)
C(3)-C(5)-C(6)-C(1)	-15.4(2)
C(19B)-C(5)-C(6)-C(1)	109.2(5)
O(1B)-C(1)-C(6)-C(21B)	-5.8(9)
C(2)-C(1)-C(6)-C(21B)	111.5(6)
C(2)-C(1)-C(6)-C(21A)	95.5(5)
O(1A)-C(1)-C(6)-C(21A)	-29.6(8)
O(1B)-C(1)-C(6)-C(5)	-127.7(7)
C(2)-C(1)-C(6)-C(5)	-10.4(2)
O(1A)-C(1)-C(6)-C(5)	-135.5(6)
O(1B)-Si(1B)-C(9B)-C(10B)	61.2(17)
C(8B)-Si(1B)-C(9B)-C(10B)	-57.7(16)
C(7B)-Si(1B)-C(9B)-C(10B)	179.5(15)
O(1B)-Si(1B)-C(9B)-C(11B)	-58.1(10)
C(8B)-Si(1B)-C(9B)-C(11B)	-176.9(7)
C(7B)-Si(1B)-C(9B)-C(11B)	60.2(9)
O(1B)-Si(1B)-C(9B)-C(12B)	179.5(9)
C(8B)-Si(1B)-C(9B)-C(12B)	60.7(10)
C(7B)-Si(1B)-C(9B)-C(12B)	-62.2(10)
O(2A)-Si(2A)-C(15A)-C(18A)	-178.4(8)
C(13A)-Si(2A)-C(15A)-C(18A)	-62.3(10)
C(14A)-Si(2A)-C(15A)-C(18A)	62.0(8)
O(2A)-Si(2A)-C(15A)-C(16A)	60.6(6)
C(13A)-Si(2A)-C(15A)-C(16A)	176.7(7)
C(14A)-Si(2A)-C(15A)-C(16A)	-59.0(5)
O(2A)-Si(2A)-C(15A)-C(17A)	-58.8(6)
C(13A)-Si(2A)-C(15A)-C(17A)	57.4(7)
C(14A)-Si(2A)-C(15A)-C(17A)	-178.4(4)
O(2B)-Si(2B)-C(15B)-C(17B)	75.0(6)
C(14B)-Si(2B)-C(15B)-C(17B)	-169.1(5)
C(13B)-Si(2B)-C(15B)-C(17B)	-49.8(9)
O(2B)-Si(2B)-C(15B)-C(16B)	-45.9(6)
C(14B)-Si(2B)-C(15B)-C(16B)	70.1(5)
C(13B)-Si(2B)-C(15B)-C(16B)	-170.7(8)
O(2B)-Si(2B)-C(15B)-C(18B)	-166.5(10)
C(14B)-Si(2B)-C(15B)-C(18B)	-50.5(10)
C(13B)-Si(2B)-C(15B)-C(18B)	68.7(12)
C(3)-C(5)-C(19A)-O(3A)	-55.1(13)
C(6)-C(5)-C(19A)-O(3A)	-174.4(11)

C(3)-C(5)-C(19A)-C(20A)	129.0(6)
C(6)-C(5)-C(19A)-C(20A)	9.7(8)
C(3)-C(5)-C(19B)-O(3B)	-62.9(13)
C(6)-C(5)-C(19B)-O(3B)	-175.3(11)
C(3)-C(5)-C(19B)-C(20B)	120.1(7)
C(6)-C(5)-C(19B)-C(20B)	7.8(8)
O(3A)-C(19A)-C(20A)-O(4A)	-2.6(15)
C(5)-C(19A)-C(20A)-O(4A)	173.7(7)
O(3A)-C(19A)-C(20A)-C(21A)	-179.2(11)
C(5)-C(19A)-C(20A)-C(21A)	-2.9(11)
O(3B)-C(19B)-C(20B)-C(21B)	-179.8(12)
C(5)-C(19B)-C(20B)-C(21B)	-3.1(12)
O(3B)-C(19B)-C(20B)-O(4B)	-2.3(16)
C(5)-C(19B)-C(20B)-O(4B)	174.4(8)
O(4A)-C(20A)-C(21A)-C(22A)	-2.7(17)
C(19A)-C(20A)-C(21A)-C(22A)	173.6(10)
O(4A)-C(20A)-C(21A)-C(6)	178.7(8)
C(19A)-C(20A)-C(21A)-C(6)	-5.1(12)
C(5)-C(6)-C(21A)-C(20A)	10.1(9)
C(1)-C(6)-C(21A)-C(20A)	-101.1(9)
C(5)-C(6)-C(21A)-C(22A)	-168.6(10)
C(1)-C(6)-C(21A)-C(22A)	80.2(11)
O(4B)-C(20B)-C(21B)-C(22B)	-4.9(17)
C(19B)-C(20B)-C(21B)-C(22B)	172.3(9)
O(4B)-C(20B)-C(21B)-C(6)	179.2(9)
C(19B)-C(20B)-C(21B)-C(6)	-3.6(12)
C(5)-C(6)-C(21B)-C(20B)	9.5(10)
C(1)-C(6)-C(21B)-C(20B)	-107.8(8)
C(5)-C(6)-C(21B)-C(22B)	-165.6(10)
C(1)-C(6)-C(21B)-C(22B)	77.0(13)
C(23A)-O(6A)-C(22A)-O(5A)	-4.2(12)
C(23A)-O(6A)-C(22A)-C(21A)	170.5(8)
C(20A)-C(21A)-C(22A)-O(5A)	5.0(16)
C(6)-C(21A)-C(22A)-O(5A)	-176.4(8)
C(20A)-C(21A)-C(22A)-O(6A)	-169.5(9)
C(6)-C(21A)-C(22A)-O(6A)	9.0(14)
C(23B)-O(6B)-C(22B)-O(5B)	-8.2(12)
C(23B)-O(6B)-C(22B)-C(21B)	175.2(8)
C(20B)-C(21B)-C(22B)-O(5B)	9.2(15)
C(6)-C(21B)-C(22B)-O(5B)	-176.1(10)
C(20B)-C(21B)-C(22B)-O(6B)	-174.1(9)
C(6)-C(21B)-C(22B)-O(6B)	0.6(16)

Symmetry transformations used to generate equivalent atoms:

Pentacycle 16: (grown via slow evaporation from EtOAc/CH_2Cl_2/hexanes at 23 °C)

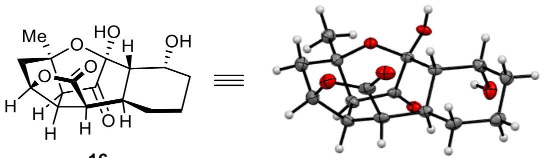

16

X-ray
[CCDC 2491903]

A colorless plate 0.13 x 0.09 x 0.04 mm in size was mounted on a Cryoloop with Paratone oil. Data were collected in a nitrogen gas stream at 100(2) K using omega scans. Crystal-to-detector distance was 31.11 mm and exposure time was 0.50 seconds per frame at low angles and 2.00 seconds at high angles, using a scan width of 0.5°. Data collection was 100% complete to 74.000° in θ. A total of 17382 reflections were collected covering the indices -11<=h<=12, -6<=k<=6, -18<=l<=18. 3189 reflections were found to be symmetry independent, with an R_{int} of 0.0380. Indexing and unit cell refinement indicated a primitive, monoclinic lattice. The space group was found to be P 21 (No. 4). The data were integrated using the CrysAlis^{Pro} 1.172.43.143a software program and scaled using the SCALE3 ABSPACK scaling algorithm. Solution by intrinsic phasing (SHELXT-2015) produced a heavy-atom phasing model consistent with the proposed structure. All non-hydrogen atoms were refined anisotropically by full-matrix least-squares (SHELXL-2014). All hydrogen atoms were placed using a riding model. Their positions were constrained relative to their parent atom using the appropriate HFIX command in SHELXL-2014. A solvent mask was applied within the Olex2 software package to mask the electron density associated with a highly disordered outer-sphere solvent molecule. The identity of this solvent could not be definitively identified, so it was not included in the overall empirical formula of the crystallized molecule.

Table 1. Crystal data and structure refinement for KYu08_Maimone.

Identification code	KYu08_Maimone	
Empirical formula	C16 H20 O6	
Formula weight	308.32	
Temperature	100(2) K	
Wavelength	1.54184 Å	
Crystal system	Monoclinic	
Space group	P 21	
Unit cell dimensions	a = 9.6782(2) Å	α= 90°.
	b = 5.48080(10) Å	β= 92.8280(10)°.
	c = 15.0301(2) Å	γ = 90°.
Volume	796.29(2) Å^3	
Z	2	
Density (calculated)	1.286 Mg/m^3	
Absorption coefficient	0.823 mm^{-1}	
F(000)	328	
Crystal size	0.130 x 0.090 x 0.040 mm^3	
Theta range for data collection	4.574 to 74.501°.	
Index ranges	-11<=h<=12, -6<=k<=6, -18<=l<=18	
Reflections collected	17382	
Independent reflections	3189 [R(int) = 0.0380]	
Completeness to theta = 74.000°	99.9 %	
Absorption correction	Semi-empirical from equivalents	
Max. and min. transmission	1.00000 and 0.87966	
Refinement method	Full-matrix least-squares on F^2	
Data / restraints / parameters	3189 / 1 / 208	
Goodness-of-fit on F^2	1.066	
Final R indices [I>2sigma(I)]	R1 = 0.0288, wR2 = 0.0719	
R indices (all data)	R1 = 0.0308, wR2 = 0.0738	
Absolute structure parameter	-0.01(7)	
Extinction coefficient	n/a	
Largest diff. peak and hole	0.160 and -0.150 e.Å^{-3}	

Table 2. Atomic coordinates (x 10^4) and equivalent isotropic displacement parameters ($Å^2$x 10^3) for KYu08_Maimone. U(eq) is defined as one third of the trace of the orthogonalized U^{ij} tensor.

	x	y	z	U(eq)
O(1)	2750(1)	7019(3)	3828(1)	19(1)
O(2)	328(1)	4691(3)	2735(1)	29(1)
O(3)	4474(2)	8027(3)	608(1)	29(1)
O(4)	6293(2)	6235(3)	1277(1)	29(1)
O(5)	4694(1)	6939(3)	2959(1)	19(1)
O(6)	2693(1)	2007(3)	3575(1)	21(1)
C(1)	3322(2)	6048(4)	3066(1)	17(1)
C(2)	2455(2)	6503(4)	2189(1)	18(1)
C(3)	909(2)	6828(4)	2348(1)	23(1)
C(4)	88(2)	7290(4)	1476(1)	26(1)
C(5)	283(2)	5208(4)	818(1)	28(1)
C(6)	1811(2)	4896(4)	641(1)	25(1)
C(7)	2683(2)	4440(4)	1510(1)	20(1)
C(8)	4211(2)	4013(4)	1301(1)	22(1)
C(9)	4944(2)	6300(4)	1020(1)	25(1)
C(10)	6600(2)	4240(4)	1893(1)	28(1)
C(11)	5245(2)	2738(4)	1977(1)	23(1)
C(12)	6994(2)	5289(5)	2807(1)	28(1)
C(13)	5694(2)	5124(4)	3326(1)	22(1)
C(14)	5922(2)	5408(4)	4325(1)	26(1)
C(15)	4981(2)	2758(4)	2997(1)	22(1)
C(16)	3531(2)	3307(4)	3238(1)	18(1)

Table 3. Bond lengths [Å] and angles [°] for KYu08_Maimone.

O(1)-C(1)	1.402(2)
O(1)-H(1)	0.84(4)
O(2)-C(3)	1.435(3)
O(2)-H(2)	0.87(4)
O(3)-C(9)	1.208(3)
O(4)-C(9)	1.344(3)
O(4)-C(10)	1.453(3)
O(5)-C(1)	1.431(2)
O(5)-C(13)	1.476(2)
O(6)-C(16)	1.210(2)
C(1)-C(16)	1.536(3)
C(1)-C(2)	1.547(2)
C(2)-C(3)	1.537(3)
C(2)-C(7)	1.546(3)
C(2)-H(2A)	1.0000
C(3)-C(4)	1.519(3)
C(3)-H(3)	1.0000
C(4)-C(5)	1.528(3)
C(4)-H(4A)	0.9900
C(4)-H(4B)	0.9900
C(5)-C(6)	1.525(3)
C(5)-H(5A)	0.9900
C(5)-H(5B)	0.9900
C(6)-C(7)	1.540(3)
C(6)-H(6A)	0.9900
C(6)-H(6B)	0.9900
C(7)-C(8)	1.544(3)
C(7)-H(7)	1.0000
C(8)-C(9)	1.511(3)
C(8)-C(11)	1.556(3)
C(8)-H(8)	1.0000
C(10)-C(12)	1.521(3)
C(10)-C(11)	1.560(3)
C(10)-H(10)	1.0000
C(11)-C(15)	1.567(3)
C(11)-H(11)	1.0000
C(12)-C(13)	1.515(3)
C(12)-H(12A)	0.9900
C(12)-H(12B)	0.9900
C(13)-C(14)	1.515(3)

C(13)-C(15)	1.539(3)
C(14)-H(14A)	0.9800
C(14)-H(14B)	0.9800
C(14)-H(14C)	0.9800
C(15)-C(16)	1.497(3)
C(15)-H(15)	1.0000

C(1)-O(1)-H(1)	108(2)
C(3)-O(2)-H(2)	109(3)
C(9)-O(4)-C(10)	111.61(16)
C(1)-O(5)-C(13)	108.88(14)
O(1)-C(1)-O(5)	111.58(15)
O(1)-C(1)-C(16)	106.70(15)
O(5)-C(1)-C(16)	103.70(15)
O(1)-C(1)-C(2)	114.58(16)
O(5)-C(1)-C(2)	108.49(14)
C(16)-C(1)-C(2)	111.26(15)
C(3)-C(2)-C(7)	111.04(15)
C(3)-C(2)-C(1)	112.18(14)
C(7)-C(2)-C(1)	110.80(15)
C(3)-C(2)-H(2A)	107.5
C(7)-C(2)-H(2A)	107.5
C(1)-C(2)-H(2A)	107.5
O(2)-C(3)-C(4)	106.71(17)
O(2)-C(3)-C(2)	111.85(16)
C(4)-C(3)-C(2)	110.89(15)
O(2)-C(3)-H(3)	109.1
C(4)-C(3)-H(3)	109.1
C(2)-C(3)-H(3)	109.1
C(3)-C(4)-C(5)	110.96(17)
C(3)-C(4)-H(4A)	109.4
C(5)-C(4)-H(4A)	109.4
C(3)-C(4)-H(4B)	109.4
C(5)-C(4)-H(4B)	109.4
H(4A)-C(4)-H(4B)	108.0
C(6)-C(5)-C(4)	110.40(17)
C(6)-C(5)-H(5A)	109.6
C(4)-C(5)-H(5A)	109.6
C(6)-C(5)-H(5B)	109.6
C(4)-C(5)-H(5B)	109.6
H(5A)-C(5)-H(5B)	108.1
C(5)-C(6)-C(7)	111.45(16)

C(5)-C(6)-H(6A)	109.3
C(7)-C(6)-H(6A)	109.3
C(5)-C(6)-H(6B)	109.3
C(7)-C(6)-H(6B)	109.3
H(6A)-C(6)-H(6B)	108.0
C(6)-C(7)-C(8)	110.00(15)
C(6)-C(7)-C(2)	110.54(16)
C(8)-C(7)-C(2)	114.51(15)
C(6)-C(7)-H(7)	107.1
C(8)-C(7)-H(7)	107.1
C(2)-C(7)-H(7)	107.1
C(9)-C(8)-C(7)	113.49(17)
C(9)-C(8)-C(11)	105.01(16)
C(7)-C(8)-C(11)	121.78(15)
C(9)-C(8)-H(8)	105.0
C(7)-C(8)-H(8)	105.0
C(11)-C(8)-H(8)	105.0
O(3)-C(9)-O(4)	120.26(19)
O(3)-C(9)-C(8)	128.6(2)
O(4)-C(9)-C(8)	111.15(19)
O(4)-C(10)-C(12)	109.00(18)
O(4)-C(10)-C(11)	107.58(17)
C(12)-C(10)-C(11)	107.50(16)
O(4)-C(10)-H(10)	110.9
C(12)-C(10)-H(10)	110.9
C(11)-C(10)-H(10)	110.9
C(8)-C(11)-C(10)	103.11(16)
C(8)-C(11)-C(15)	120.35(16)
C(10)-C(11)-C(15)	104.70(16)
C(8)-C(11)-H(11)	109.3
C(10)-C(11)-H(11)	109.3
C(15)-C(11)-H(11)	109.3
C(13)-C(12)-C(10)	105.34(16)
C(13)-C(12)-H(12A)	110.7
C(10)-C(12)-H(12A)	110.7
C(13)-C(12)-H(12B)	110.7
C(10)-C(12)-H(12B)	110.7
H(12A)-C(12)-H(12B)	108.8
O(5)-C(13)-C(12)	108.31(16)
O(5)-C(13)-C(14)	111.20(16)
C(12)-C(13)-C(14)	114.81(17)
O(5)-C(13)-C(15)	99.96(14)

C(12)-C(13)-C(15)	104.82(16)
C(14)-C(13)-C(15)	116.45(18)
C(13)-C(14)-H(14A)	109.5
C(13)-C(14)-H(14B)	109.5
H(14A)-C(14)-H(14B)	109.5
C(13)-C(14)-H(14C)	109.5
H(14A)-C(14)-H(14C)	109.5
H(14B)-C(14)-H(14C)	109.5
C(16)-C(15)-C(13)	99.41(16)
C(16)-C(15)-C(11)	115.94(16)
C(13)-C(15)-C(11)	103.18(16)
C(16)-C(15)-H(15)	112.4
C(13)-C(15)-H(15)	112.4
C(11)-C(15)-H(15)	112.4
O(6)-C(16)-C(15)	129.71(19)
O(6)-C(16)-C(1)	124.10(18)
C(15)-C(16)-C(1)	105.89(16)

Symmetry transformations used to generate equivalent atoms:

Table 4. Anisotropic displacement parameters ($Å^2$x 10^3) for KYu08_Maimone. The anisotropic displacement factor exponent takes the form: $-2\pi^2[h^2a^{*2}U^{11} + ... + 2 h k a^* b^* U^{12}]$

	U^{11}	U^{22}	U^{33}	U^{23}	U^{13}	U^{12}
O(1)	29(1)	15(1)	14(1)	-1(1)	5(1)	2(1)
O(2)	23(1)	35(1)	30(1)	10(1)	4(1)	-1(1)
O(3)	43(1)	25(1)	21(1)	0(1)	12(1)	-5(1)
O(4)	30(1)	32(1)	26(1)	-2(1)	11(1)	-9(1)
O(5)	22(1)	15(1)	20(1)	1(1)	2(1)	-2(1)
O(6)	27(1)	16(1)	21(1)	1(1)	4(1)	-1(1)
C(1)	21(1)	14(1)	16(1)	0(1)	4(1)	-1(1)
C(2)	24(1)	15(1)	15(1)	1(1)	1(1)	-1(1)
C(3)	26(1)	23(1)	20(1)	0(1)	2(1)	5(1)
C(4)	26(1)	26(1)	24(1)	2(1)	-3(1)	4(1)
C(5)	32(1)	26(1)	24(1)	0(1)	-6(1)	-2(1)
C(6)	34(1)	26(1)	16(1)	-2(1)	-2(1)	-4(1)
C(7)	23(1)	19(1)	17(1)	-1(1)	2(1)	-3(1)
C(8)	30(1)	20(1)	15(1)	-5(1)	8(1)	-3(1)
C(9)	33(1)	26(1)	17(1)	-7(1)	11(1)	-7(1)
C(10)	22(1)	30(1)	32(1)	-6(1)	8(1)	-1(1)
C(11)	24(1)	20(1)	25(1)	-4(1)	7(1)	1(1)
C(12)	22(1)	30(1)	34(1)	-4(1)	2(1)	-2(1)
C(13)	21(1)	20(1)	24(1)	1(1)	-1(1)	1(1)
C(14)	27(1)	26(1)	25(1)	-1(1)	-3(1)	1(1)
C(15)	24(1)	16(1)	24(1)	0(1)	4(1)	3(1)
C(16)	23(1)	17(1)	13(1)	-2(1)	0(1)	0(1)

Table 5. Hydrogen coordinates (x 10^4) and isotropic displacement parameters ($Å^2$x 10^3) for KYu08_Maimone.

	x	y	z	U(eq)
H(2A)	2791	8056	1925	22
H(3)	790	8247	2755	27
H(4A)	397	8841	1213	31
H(4B)	-905	7451	1593	31
H(5A)	-80	3674	1064	33
H(5B)	-244	5563	252	33
H(6A)	2150	6382	348	31
H(6B)	1922	3503	231	31
H(7)	2338	2896	1775	24
H(8)	4175	2939	762	26
H(10)	7359	3190	1676	33
H(11)	5402	1023	1779	27
H(12A)	7752	4331	3103	34
H(12B)	7299	7006	2758	34
H(14A)	6342	7001	4459	39
H(14B)	6538	4112	4556	39
H(14C)	5032	5293	4607	39
H(15)	5366	1280	3311	26
H(1)	2740(30)	8550(70)	3770(20)	47(9)
H(2)	800(40)	4360(80)	3230(20)	65(10)

Table 6. Torsion angles [°] for KYu08_Maimone.

C(13)-O(5)-C(1)-O(1)	97.15(17)
C(13)-O(5)-C(1)-C(16)	-17.34(17)
C(13)-O(5)-C(1)-C(2)	-135.69(15)
O(1)-C(1)-C(2)-C(3)	-26.4(2)
O(5)-C(1)-C(2)-C(3)	-151.78(16)
C(16)-C(1)-C(2)-C(3)	94.77(19)
O(1)-C(1)-C(2)-C(7)	-151.09(16)
O(5)-C(1)-C(2)-C(7)	83.49(18)
C(16)-C(1)-C(2)-C(7)	-30.0(2)
C(7)-C(2)-C(3)-O(2)	63.15(19)
C(1)-C(2)-C(3)-O(2)	-61.4(2)
C(7)-C(2)-C(3)-C(4)	-55.8(2)
C(1)-C(2)-C(3)-C(4)	179.56(17)
O(2)-C(3)-C(4)-C(5)	-64.4(2)
C(2)-C(3)-C(4)-C(5)	57.7(2)
C(3)-C(4)-C(5)-C(6)	-58.1(2)
C(4)-C(5)-C(6)-C(7)	57.0(2)
C(5)-C(6)-C(7)-C(8)	177.34(18)
C(5)-C(6)-C(7)-C(2)	-55.2(2)
C(3)-C(2)-C(7)-C(6)	54.3(2)
C(1)-C(2)-C(7)-C(6)	179.66(15)
C(3)-C(2)-C(7)-C(8)	179.20(16)
C(1)-C(2)-C(7)-C(8)	-55.4(2)
C(6)-C(7)-C(8)-C(9)	72.3(2)
C(2)-C(7)-C(8)-C(9)	-52.9(2)
C(6)-C(7)-C(8)-C(11)	-160.76(18)
C(2)-C(7)-C(8)-C(11)	74.0(2)
C(10)-O(4)-C(9)-O(3)	169.85(17)
C(10)-O(4)-C(9)-C(8)	-11.2(2)
C(7)-C(8)-C(9)-O(3)	-32.8(3)
C(11)-C(8)-C(9)-O(3)	-168.10(18)
C(7)-C(8)-C(9)-O(4)	148.34(16)
C(11)-C(8)-C(9)-O(4)	13.0(2)
C(9)-O(4)-C(10)-C(12)	-111.81(18)
C(9)-O(4)-C(10)-C(11)	4.5(2)
C(9)-C(8)-C(11)-C(10)	-9.39(19)
C(7)-C(8)-C(11)-C(10)	-140.04(18)
C(9)-C(8)-C(11)-C(15)	106.61(19)
C(7)-C(8)-C(11)-C(15)	-24.0(3)
O(4)-C(10)-C(11)-C(8)	3.62(19)

C(12)-C(10)-C(11)-C(8)	120.87(18)
O(4)-C(10)-C(11)-C(15)	-123.08(17)
C(12)-C(10)-C(11)-C(15)	-5.8(2)
O(4)-C(10)-C(12)-C(13)	98.64(19)
C(11)-C(10)-C(12)-C(13)	-17.7(2)
C(1)-O(5)-C(13)-C(12)	149.62(15)
C(1)-O(5)-C(13)-C(14)	-83.34(19)
C(1)-O(5)-C(13)-C(15)	40.25(17)
C(10)-C(12)-C(13)-O(5)	-71.1(2)
C(10)-C(12)-C(13)-C(14)	163.95(19)
C(10)-C(12)-C(13)-C(15)	34.9(2)
O(5)-C(13)-C(15)-C(16)	-45.64(16)
C(12)-C(13)-C(15)-C(16)	-157.76(15)
C(14)-C(13)-C(15)-C(16)	74.2(2)
O(5)-C(13)-C(15)-C(11)	73.97(17)
C(12)-C(13)-C(15)-C(11)	-38.14(19)
C(14)-C(13)-C(15)-C(11)	-166.19(16)
C(8)-C(11)-C(15)-C(16)	18.9(3)
C(10)-C(11)-C(15)-C(16)	134.07(19)
C(8)-C(11)-C(15)-C(13)	-88.6(2)
C(10)-C(11)-C(15)-C(13)	26.6(2)
C(13)-C(15)-C(16)-O(6)	-137.2(2)
C(11)-C(15)-C(16)-O(6)	113.1(2)
C(13)-C(15)-C(16)-C(1)	36.62(17)
C(11)-C(15)-C(16)-C(1)	-73.1(2)
O(1)-C(1)-C(16)-O(6)	43.0(2)
O(5)-C(1)-C(16)-O(6)	160.90(16)
C(2)-C(1)-C(16)-O(6)	-82.7(2)
O(1)-C(1)-C(16)-C(15)	-131.27(15)
O(5)-C(1)-C(16)-C(15)	-13.34(17)
C(2)-C(1)-C(16)-C(15)	103.08(17)

Symmetry transformations used to generate equivalent atoms:

Chloride 17: (grown via slow evaporation from EtOAc/CH_2Cl_2/hexanes at 23 °C)

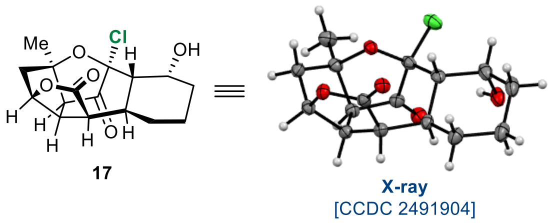

A colorless plate 0.14 x 0.06 x 0.02 mm in size was mounted on a Cryoloop with Paratone oil. Data were collected in a nitrogen gas stream at 100(2) K using omega scans. Crystal-to-detector distance was 31.11 mm and exposure time was 0.50 seconds per frame at low angles and 1.50 seconds at high angles, using a scan width of 0.5°. Data collection was 100% complete to 74.000° in θ. A total of 24533 reflections were collected covering the indices -8<=h<=8, -18<=k<=18, -19<=l<=15. 2966 reflections were found to be symmetry independent, with an R_{int} of 0.0493. Indexing and unit cell refinement indicated a primitive, orthorhombic lattice. The space group was found to be P 21 21 21 (No. 19). The data were integrated using the CrysAlis^{Pro} 1.172.43.143a software program and scaled using the SCALE3 ABSPACK scaling algorithm. Solution by intrinsic phasing (SHELXT-2015) produced a heavy-atom phasing model consistent with the proposed structure. All non-hydrogen atoms were refined anisotropically by full-matrix least-squares (SHELXL-2014). All hydrogen atoms were placed using a riding model. Their positions were constrained relative to their parent atom using the appropriate HFIX command in SHELXL-2014.

Table 1. Crystal data and structure refinement for KYu07_Maimone.

Identification code	KYu07_Maimone	
Empirical formula	C16 H19 Cl O5	
Formula weight	326.76	
Temperature	100(2) K	
Wavelength	1.54184 Å	
Crystal system	Orthorhombic	
Space group	P 21 21 21	
Unit cell dimensions	a = 6.49347(11) Å	α= 90°.
	b = 14.6375(3) Å	β= 90°.
	c = 15.4374(3) Å	γ = 90°.
Volume	1467.30(5) $Å^3$	
Z	4	
Density (calculated)	1.479 Mg/m^3	
Absorption coefficient	2.511 mm^{-1}	
F(000)	688	
Crystal size	0.140 x 0.060 x 0.020 mm^3	
Theta range for data collection	4.162 to 74.491°.	
Index ranges	-8<=h<=8, -18<=k<=18, -19<=l<=15	
Reflections collected	24533	
Independent reflections	2966 [R(int) = 0.0493]	
Completeness to theta = 74.000°	99.9 %	
Absorption correction	Semi-empirical from equivalents	
Max. and min. transmission	1.00000 and 0.81604	
Refinement method	Full-matrix least-squares on F^2	
Data / restraints / parameters	2966 / 0 / 204	
Goodness-of-fit on F^2	1.029	
Final R indices [I>2sigma(I)]	R1 = 0.0299, wR2 = 0.0747	
R indices (all data)	R1 = 0.0316, wR2 = 0.0756	
Absolute structure parameter	0.003(7)	
Extinction coefficient	n/a	
Largest diff. peak and hole	0.215 and -0.213 e.$Å^{-3}$	

Table 2. Atomic coordinates (x 10^4) and equivalent isotropic displacement parameters ($Å^2$x 10^3) for KYu07_Maimone. U(eq) is defined as one third of the trace of the orthogonalized U^{ij} tensor.

	x	y	z	U(eq)
Cl(1)	-194(1)	5271(1)	7350(1)	33(1)
O(1)	3294(3)	3816(1)	6763(1)	33(1)
O(2)	4042(3)	6806(1)	4511(1)	24(1)
O(3)	4359(3)	7909(1)	5485(1)	23(1)
O(4)	1598(3)	6722(1)	6653(1)	22(1)
O(5)	4258(3)	5268(1)	8037(1)	29(1)
C(1)	2074(4)	5817(2)	6848(2)	23(1)
C(2)	2811(4)	5305(2)	6036(2)	24(1)
C(3)	2357(4)	4271(2)	6042(2)	30(1)
C(4)	3178(5)	3813(2)	5227(2)	36(1)
C(5)	5487(5)	3969(2)	5130(2)	36(1)
C(6)	5947(4)	4986(2)	5101(2)	30(1)
C(7)	5151(4)	5481(2)	5914(1)	23(1)
C(8)	5795(4)	6492(2)	5884(1)	22(1)
C(9)	4636(4)	7044(2)	5219(1)	22(1)
C(10)	4930(4)	8028(2)	6387(1)	22(1)
C(11)	2996(4)	8226(2)	6915(2)	23(1)
C(12)	2436(4)	7329(2)	7336(2)	23(1)
C(13)	970(4)	7405(2)	8097(2)	30(1)
C(14)	4493(4)	6843(2)	7515(1)	22(1)
C(15)	5824(4)	7101(2)	6711(1)	21(1)
C(16)	3768(4)	5874(2)	7552(2)	23(1)

Table 3. Bond lengths [Å] and angles [°] for KYu07_Maimone.

Cl(1)-C(1)	1.847(3)
O(1)-C(3)	1.433(3)
O(1)-H(1)	0.85(4)
O(2)-C(9)	1.210(3)
O(3)-C(9)	1.343(3)
O(3)-C(10)	1.452(3)
O(4)-C(1)	1.394(3)
O(4)-C(12)	1.482(3)
O(5)-C(16)	1.204(3)
C(1)-C(2)	1.536(3)
C(1)-C(16)	1.549(3)
C(2)-C(3)	1.542(3)
C(2)-C(7)	1.552(3)
C(2)-H(2)	1.0000
C(3)-C(4)	1.522(4)
C(3)-H(3)	1.0000
C(4)-C(5)	1.524(5)
C(4)-H(4A)	0.9900
C(4)-H(4B)	0.9900
C(5)-C(6)	1.519(4)
C(5)-H(5A)	0.9900
C(5)-H(5B)	0.9900
C(6)-C(7)	1.539(3)
C(6)-H(6A)	0.9900
C(6)-H(6B)	0.9900
C(7)-C(8)	1.539(3)
C(7)-H(7)	1.0000
C(8)-C(9)	1.508(3)
C(8)-C(15)	1.557(3)
C(8)-H(8)	1.0000
C(10)-C(11)	1.525(3)
C(10)-C(15)	1.558(3)
C(10)-H(10)	1.0000
C(11)-C(12)	1.510(3)
C(11)-H(11A)	0.9900
C(11)-H(11B)	0.9900
C(12)-C(13)	1.516(3)
C(12)-C(14)	1.538(3)
C(13)-H(13A)	0.9800
C(13)-H(13B)	0.9800

C(13)-H(13C)	0.9800
C(14)-C(16)	1.496(3)
C(14)-C(15)	1.560(3)
C(14)-H(14)	1.0000
C(15)-H(15)	1.0000

C(3)-O(1)-H(1)	109(3)
C(9)-O(3)-C(10)	111.87(17)
C(1)-O(4)-C(12)	109.53(17)
O(4)-C(1)-C(2)	110.86(19)
O(4)-C(1)-C(16)	104.94(19)
C(2)-C(1)-C(16)	112.2(2)
O(4)-C(1)-Cl(1)	109.01(17)
C(2)-C(1)-Cl(1)	112.36(16)
C(16)-C(1)-Cl(1)	107.16(16)
C(1)-C(2)-C(3)	114.5(2)
C(1)-C(2)-C(7)	108.87(19)
C(3)-C(2)-C(7)	110.5(2)
C(1)-C(2)-H(2)	107.6
C(3)-C(2)-H(2)	107.6
C(7)-C(2)-H(2)	107.6
O(1)-C(3)-C(4)	106.8(2)
O(1)-C(3)-C(2)	112.3(2)
C(4)-C(3)-C(2)	111.2(2)
O(1)-C(3)-H(3)	108.8
C(4)-C(3)-H(3)	108.8
C(2)-C(3)-H(3)	108.8
C(3)-C(4)-C(5)	111.1(2)
C(3)-C(4)-H(4A)	109.4
C(5)-C(4)-H(4A)	109.4
C(3)-C(4)-H(4B)	109.4
C(5)-C(4)-H(4B)	109.4
H(4A)-C(4)-H(4B)	108.0
C(6)-C(5)-C(4)	110.1(2)
C(6)-C(5)-H(5A)	109.6
C(4)-C(5)-H(5A)	109.6
C(6)-C(5)-H(5B)	109.6
C(4)-C(5)-H(5B)	109.6
H(5A)-C(5)-H(5B)	108.2
C(5)-C(6)-C(7)	111.8(2)
C(5)-C(6)-H(6A)	109.3
C(7)-C(6)-H(6A)	109.3

C(5)-C(6)-H(6B)	109.3
C(7)-C(6)-H(6B)	109.3
H(6A)-C(6)-H(6B)	107.9
C(6)-C(7)-C(8)	109.7(2)
C(6)-C(7)-C(2)	110.5(2)
C(8)-C(7)-C(2)	115.40(19)
C(6)-C(7)-H(7)	107.0
C(8)-C(7)-H(7)	107.0
C(2)-C(7)-H(7)	107.0
C(9)-C(8)-C(7)	113.60(19)
C(9)-C(8)-C(15)	104.91(19)
C(7)-C(8)-C(15)	121.95(19)
C(9)-C(8)-H(8)	105.0
C(7)-C(8)-H(8)	105.0
C(15)-C(8)-H(8)	105.0
O(2)-C(9)-O(3)	120.3(2)
O(2)-C(9)-C(8)	128.3(2)
O(3)-C(9)-C(8)	111.31(19)
O(3)-C(10)-C(11)	108.98(19)
O(3)-C(10)-C(15)	107.33(17)
C(11)-C(10)-C(15)	107.53(18)
O(3)-C(10)-H(10)	111.0
C(11)-C(10)-H(10)	111.0
C(15)-C(10)-H(10)	111.0
C(12)-C(11)-C(10)	105.28(19)
C(12)-C(11)-H(11A)	110.7
C(10)-C(11)-H(11A)	110.7
C(12)-C(11)-H(11B)	110.7
C(10)-C(11)-H(11B)	110.7
H(11A)-C(11)-H(11B)	108.8
O(4)-C(12)-C(11)	107.66(18)
O(4)-C(12)-C(13)	111.4(2)
C(11)-C(12)-C(13)	114.9(2)
O(4)-C(12)-C(14)	99.78(18)
C(11)-C(12)-C(14)	105.64(19)
C(13)-C(12)-C(14)	116.1(2)
C(12)-C(13)-H(13A)	109.5
C(12)-C(13)-H(13B)	109.5
H(13A)-C(13)-H(13B)	109.5
C(12)-C(13)-H(13C)	109.5
H(13A)-C(13)-H(13C)	109.5
H(13B)-C(13)-H(13C)	109.5

C(16)-C(14)-C(12)	99.88(18)
C(16)-C(14)-C(15)	115.73(19)
C(12)-C(14)-C(15)	103.10(18)
C(16)-C(14)-H(14)	112.4
C(12)-C(14)-H(14)	112.4
C(15)-C(14)-H(14)	112.4
C(8)-C(15)-C(10)	103.41(17)
C(8)-C(15)-C(14)	120.49(19)
C(10)-C(15)-C(14)	105.01(19)
C(8)-C(15)-H(15)	109.1
C(10)-C(15)-H(15)	109.1
C(14)-C(15)-H(15)	109.1
O(5)-C(16)-C(14)	129.8(2)
O(5)-C(16)-C(1)	125.8(2)
C(14)-C(16)-C(1)	104.36(18)

Symmetry transformations used to generate equivalent atoms:

Table 4. Anisotropic displacement parameters ($Å^2 x 10^3$) for KYu07_Maimone. The anisotropic displacement factor exponent takes the form: $-2\pi^2[h^2a^{*2}U^{11} + ... + 2 h k a^* b^* U^{12}]$

	U^{11}	U^{22}	U^{33}	U^{23}	U^{13}	U^{12}
Cl(1)	27(1)	36(1)	36(1)	9(1)	-2(1)	-10(1)
O(1)	43(1)	22(1)	35(1)	6(1)	-14(1)	-4(1)
O(2)	26(1)	27(1)	20(1)	0(1)	-2(1)	0(1)
O(3)	30(1)	20(1)	21(1)	1(1)	-4(1)	1(1)
O(4)	23(1)	22(1)	22(1)	1(1)	-4(1)	-2(1)
O(5)	34(1)	28(1)	26(1)	7(1)	-6(1)	-2(1)
C(1)	21(1)	22(1)	27(1)	6(1)	-3(1)	-5(1)
C(2)	25(1)	22(1)	24(1)	2(1)	-8(1)	-1(1)
C(3)	35(1)	23(1)	33(1)	3(1)	-13(1)	-5(1)
C(4)	50(2)	21(1)	36(1)	-2(1)	-18(1)	-1(1)
C(5)	47(2)	26(1)	34(1)	-8(1)	-11(1)	7(1)
C(6)	36(1)	26(1)	28(1)	-4(1)	-4(1)	6(1)
C(7)	27(1)	19(1)	23(1)	-1(1)	-5(1)	3(1)
C(8)	21(1)	22(1)	22(1)	0(1)	0(1)	0(1)
C(9)	19(1)	22(1)	24(1)	2(1)	1(1)	-2(1)
C(10)	25(1)	21(1)	21(1)	1(1)	-4(1)	-2(1)
C(11)	25(1)	20(1)	25(1)	-2(1)	-4(1)	3(1)
C(12)	23(1)	26(1)	21(1)	-2(1)	-4(1)	0(1)
C(13)	27(1)	39(1)	24(1)	-2(1)	0(1)	0(1)
C(14)	23(1)	22(1)	19(1)	0(1)	-3(1)	-1(1)
C(15)	22(1)	20(1)	22(1)	1(1)	-2(1)	0(1)
C(16)	23(1)	25(1)	21(1)	1(1)	-1(1)	0(1)

Table 5. Hydrogen coordinates (x 10^4) and isotropic displacement parameters ($Å^2$x 10^3) for KYu07_Maimone.

	x	y	z	U(eq)
H(2)	2083	5576	5526	28
H(3)	833	4178	6070	36
H(4A)	2456	4062	4714	43
H(4B)	2898	3149	5255	43
H(5A)	6222	3687	5623	43
H(5B)	5980	3676	4590	43
H(6A)	5293	5256	4581	36
H(6B)	7453	5078	5053	36
H(7)	5866	5201	6423	27
H(8)	7253	6485	5675	26
H(10)	5962	8530	6454	27
H(11A)	3273	8698	7359	28
H(11B)	1868	8441	6535	28
H(13A)	-324	7684	7904	45
H(13B)	1594	7786	8548	45
H(13C)	693	6795	8331	45
H(14)	5137	7046	8071	26
H(15)	7283	7194	6898	25
H(1)	3010(60)	4110(30)	7220(30)	56(12)

Table 6. Torsion angles [°] for KYu07_Maimone.

C(12)-O(4)-C(1)-C(2)	-136.51(19)
C(12)-O(4)-C(1)-C(16)	-15.2(2)
C(12)-O(4)-C(1)-Cl(1)	99.30(18)
O(4)-C(1)-C(2)-C(3)	-151.1(2)
C(16)-C(1)-C(2)-C(3)	92.0(2)
Cl(1)-C(1)-C(2)-C(3)	-28.8(3)
O(4)-C(1)-C(2)-C(7)	84.7(2)
C(16)-C(1)-C(2)-C(7)	-32.2(3)
Cl(1)-C(1)-C(2)-C(7)	-153.04(16)
C(1)-C(2)-C(3)-O(1)	-59.1(3)
C(7)-C(2)-C(3)-O(1)	64.2(3)
C(1)-C(2)-C(3)-C(4)	-178.7(2)
C(7)-C(2)-C(3)-C(4)	-55.4(3)
O(1)-C(3)-C(4)-C(5)	-65.2(3)
C(2)-C(3)-C(4)-C(5)	57.6(3)
C(3)-C(4)-C(5)-C(6)	-58.1(3)
C(4)-C(5)-C(6)-C(7)	57.5(3)
C(5)-C(6)-C(7)-C(8)	175.9(2)
C(5)-C(6)-C(7)-C(2)	-55.9(3)
C(1)-C(2)-C(7)-C(6)	-179.48(18)
C(3)-C(2)-C(7)-C(6)	54.0(3)
C(1)-C(2)-C(7)-C(8)	-54.4(2)
C(3)-C(2)-C(7)-C(8)	179.09(19)
C(6)-C(7)-C(8)-C(9)	71.7(3)
C(2)-C(7)-C(8)-C(9)	-53.8(3)
C(6)-C(7)-C(8)-C(15)	-161.1(2)
C(2)-C(7)-C(8)-C(15)	73.4(3)
C(10)-O(3)-C(9)-O(2)	172.5(2)
C(10)-O(3)-C(9)-C(8)	-9.8(3)
C(7)-C(8)-C(9)-O(2)	-35.7(3)
C(15)-C(8)-C(9)-O(2)	-171.2(2)
C(7)-C(8)-C(9)-O(3)	146.93(19)
C(15)-C(8)-C(9)-O(3)	11.4(3)
C(9)-O(3)-C(10)-C(11)	-112.2(2)
C(9)-O(3)-C(10)-C(15)	4.0(2)
O(3)-C(10)-C(11)-C(12)	100.2(2)
C(15)-C(10)-C(11)-C(12)	-15.9(2)
C(1)-O(4)-C(12)-C(11)	148.45(19)
C(1)-O(4)-C(12)-C(13)	-84.7(2)
C(1)-O(4)-C(12)-C(14)	38.5(2)

C(10)-C(11)-C(12)-O(4)	-72.8(2)
C(10)-C(11)-C(12)-C(13)	162.4(2)
C(10)-C(11)-C(12)-C(14)	33.1(2)
O(4)-C(12)-C(14)-C(16)	-45.1(2)
C(11)-C(12)-C(14)-C(16)	-156.65(18)
C(13)-C(12)-C(14)-C(16)	74.8(2)
O(4)-C(12)-C(14)-C(15)	74.47(19)
C(11)-C(12)-C(14)-C(15)	-37.1(2)
C(13)-C(12)-C(14)-C(15)	-165.7(2)
C(9)-C(8)-C(15)-C(10)	-8.2(2)
C(7)-C(8)-C(15)-C(10)	-139.0(2)
C(9)-C(8)-C(15)-C(14)	108.5(2)
C(7)-C(8)-C(15)-C(14)	-22.3(3)
O(3)-C(10)-C(15)-C(8)	3.1(2)
C(11)-C(10)-C(15)-C(8)	120.2(2)
O(3)-C(10)-C(15)-C(14)	-124.05(19)
C(11)-C(10)-C(15)-C(14)	-6.9(2)
C(16)-C(14)-C(15)-C(8)	18.5(3)
C(12)-C(14)-C(15)-C(8)	-89.4(2)
C(16)-C(14)-C(15)-C(10)	134.4(2)
C(12)-C(14)-C(15)-C(10)	26.4(2)
C(12)-C(14)-C(16)-O(5)	-139.0(3)
C(15)-C(14)-C(16)-O(5)	111.2(3)
C(12)-C(14)-C(16)-C(1)	37.1(2)
C(15)-C(14)-C(16)-C(1)	-72.7(2)
O(4)-C(1)-C(16)-O(5)	161.4(2)
C(2)-C(1)-C(16)-O(5)	-78.2(3)
Cl(1)-C(1)-C(16)-O(5)	45.6(3)
O(4)-C(1)-C(16)-C(14)	-15.0(2)
C(2)-C(1)-C(16)-C(14)	105.5(2)
Cl(1)-C(1)-C(16)-C(14)	-130.76(17)

Symmetry transformations used to generate equivalent atoms:

VI) Abbreviations

(*S*)-BINOL	(*S*)-(−)-1,1'-binaphthalene-2,2'-diol
4CzIPN	1,2,3,5-tetrakis(carbazol-9-yl)-4,6-dicyanobenzene
dF(CF_3)ppy	2-(2,4-difluorophenyl)-5-(trifluoromethyl)pyridine
4-DMAP	*N,N'*-dimethylaminopyridine
DMAc	*N,N'*-dimethylacetamide
DMSO	dimethyl sulfoxide
dtbbpy	4,4'-di-*tert*-butyl-2,2'-bipyridyl
i-Pr	isopropyl
LiHMDS	lithium bis(trimethylsilyl)amide
2,6-lutidine	2,6-dimethylpyridine
4Å MS	4Å molecular sieve
n-Bu	*n*-butyl
Pd	palladium
PF_6	hexafluorophosphate
Py	pyridine
$Sc(OTf)_3$	Scandium trifluoromethanesulfonate
TBSCl	*tert*-butyldimethylsilyl chloride
terpy	terpyridine
TBSOTf	*tert*-butyldimethylsilyl trifluoromethanesulfonate
TFA	trifluoroacetic acid

VII) References

1. Mandal, S. K., Jensen, D. R., Pugsley, J. S. & Sigman, M. S. Scope of enantioselective Palladium(II)-catalyzed aerobic alcohol oxidations with (−)-sparteine. *J. Org. Chem.* **2003**, *68*, 4600–4603.

2. Anastasia, M., Allevi, P., Ciuffreda, P., Fiecchi, A., Gariboldi, P. & Scala. A. Conversion of nor-ketones into prochiral terminal methylene groups: synthesis of (24*E*)- and (24*Z*) - [28-^{2}H] ergosta - 5, 24 (28)-dien-3β-ols. *J. Chem. Soc., Perkin Trans. 1*, **1985**, 595–599.

3. Duh, C.-Y., Wang, S.-K., Chia, M.-C. & Chiang, M. Y. A novel cytotoxic norditerpenoid from the Formosan soft coral *Sinularia inelegans*. *Tetrahedron Lett.* **1999**, *40*, 6033−6035.

4. Tuccinardi, J. P. & Wood, J. L. Total syntheses of (+)-ineleganolide and (−)-sinulochmodin C. *J. Am. Chem. Soc.* **2022**, *144*, 20539−20547.

5. Gross, B. M., Han, S.-J., Virgil, S. C. & Stoltz, B. M. A convergent total synthesis of (+)-ineleganolide. *J. Am. Chem. Soc.* **2023**, *145*, 7763−7767.

6. Lin, D. S., Späth, G., Meng, Z., Wieske, L. H. E., Farès, C. & Fürstner, A. Total synthesis of the norcembranoid scabrolide B and its transformation into sinuscalide C, ineleganolide, and horiolide. *J. Am. Chem. Soc.* **2024**, *146*, 24250−24256.

7. Simmons, E. J., Ryffel, D. B., Lopez, D. A., Boyko, Y. D. & Sarlah, D. Total syntheses of scabrolide B, ineleganolide, and related norcembranoids. *J. Am. Chem. Soc.* **2025**, *147*, 130−135.

8. Frisch, M. J. et al. *Gaussian 16, Revision B.01* (Gaussian, 2016).

9. Schrödinger Release 2024-4: Maestro, Schrödinger, LLC, New York, NY, 2024.

10. Hohenberg, P. & Kohn, W. Inhomogeneous Electron Gas. *Phys. Rev.* **1964**, *136*, B864–B871.

11. Kohn, W. & Sham, L. J. Self-Consistent Equations Including Exchange and Correlation Effects. *Phys. Rev.* **1965**, *140*, A1133–A1138.

12. Zhao, Y. & Truhlar, D. G. The M06 suite of density functionals for main group thermochemistry, thermochemical kinetics, noncovalent interactions, excited states, and transition elements: two new

functionals and systematic testing of four M06-class functionals and 12 other functionals. *Theor. Chem. Acc.* **2008**, *120*, 215–241.

13. Grimme, S., Antony, J., Ehrlich, S. & Krieg, H. A consistent and accurate ab initio parameterization of density functional dispersion correction (DFT-D) for the 94 elements H-Pu. *J. Chem. Phys.* **2010**, *132*, 154104.

14. Weigend, F. & Ahlrichs, R. Balanced basis sets of split valence, triple zeta valence and quadruple zeta valence quality for H to Rn: Design and assessment of accuracy. *Phys. Chem. Chem. Phys.* **2005**, *7*, 3297–3305.

15. Weigend, F. Accurate Coulomb-fitting basis sets for H to Rn. *Phys. Chem. Chem. Phys.* **2006**, *8*, 1057–1065.

16. Marenich, A. V., Cramer, C. J. & Truhlar, D. G. Universal Solvation Model Based on Solute Electron Density and on a Continuum Model of the Solvent Defined by the Bulk Dielectric Constant and Atomic Surface Tensions. *J. Phys. Chem. B.* **2009**, *113*, 6378–6396.

17. Stephens, P. J., Devlin, F. J., Chabalowski, C. F. & Frisch, M. J. Ab Initio Calculation of Vibrational Absorption and Circular Dichroism Spectra Using Density Functional Force Fields. *J. Phys. Chem.* **1994**, *98*, 11623–11627.

18. Grimme, S., Ehrlich, S. & Goerigk, L. Effect of the damping function in dispersion corrected density functional theory. *J. Comp. Chem.* **2011**, *32*, 1456–1465.

19. Fukui, K. The Path of Chemical Reactions – The IRC Approach. *Acc. Chem. Res.* **1981**, *14*, 363–368.

20. Ditchfield, R., Hehre, W. J. & Pople, J. A. Self-Consistent Molecular Orbital Methods. IX. An Extended Gaussian-Type Basis for Molecular-Orbital Studies of Organic Molecules. *J. Chem. Phys.* **1971**, *54*, 724–728.

21. Legault, C. Y. CYLview20; Université de Sherbrooke, 2020 (http://www.cylview.org).

VIII) ^{1}H and ^{13}C NMR Spectra of Compounds

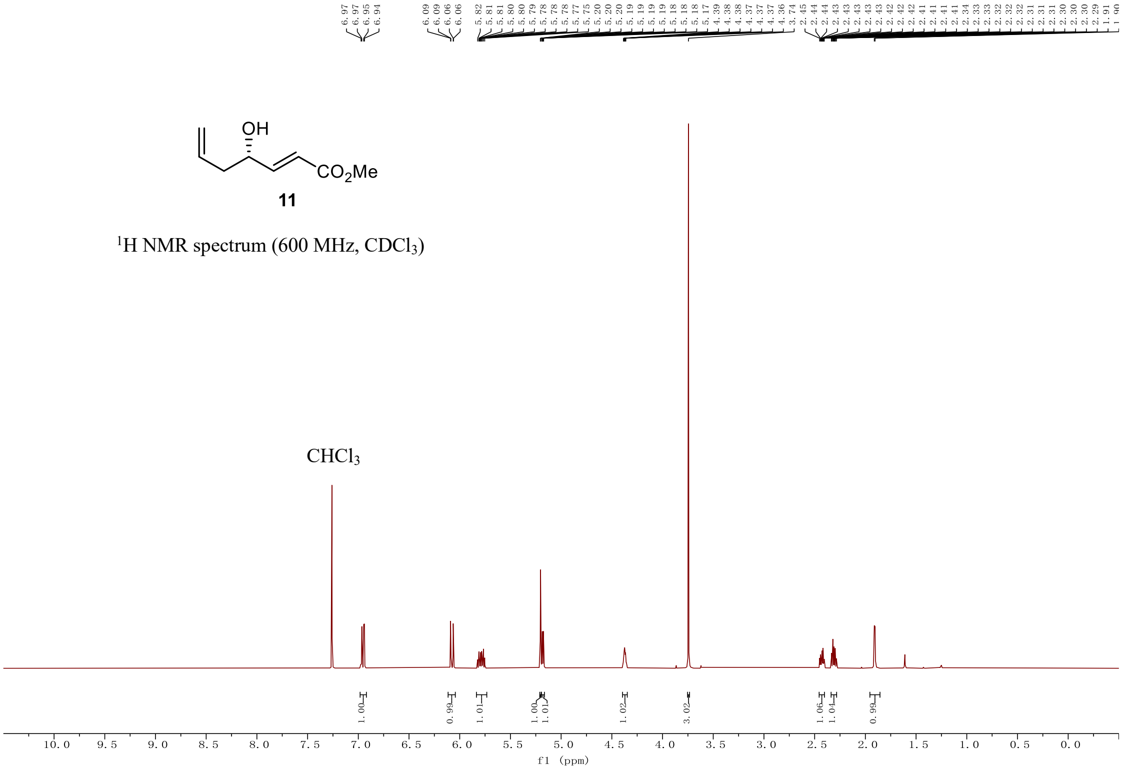

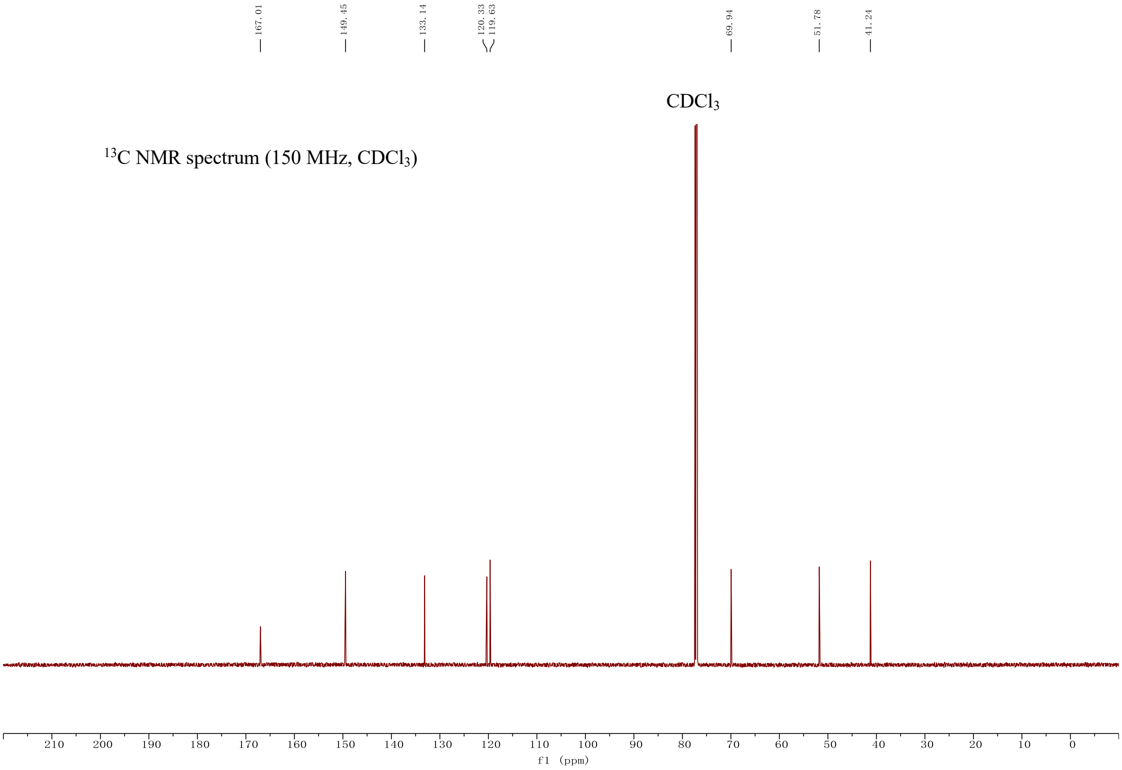

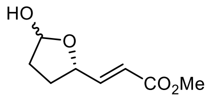

12'

(dr = 1:0.73)

^{1}H NMR spectrum (500 MHz, $CDCl_3$)

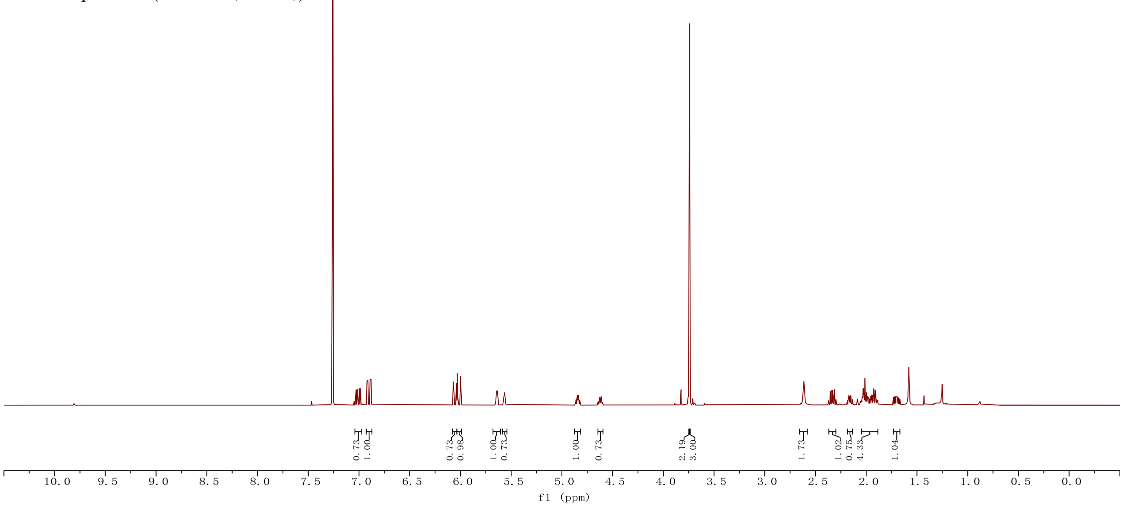

^{13}C NMR spectrum (125 MHz, $CDCl_3$)

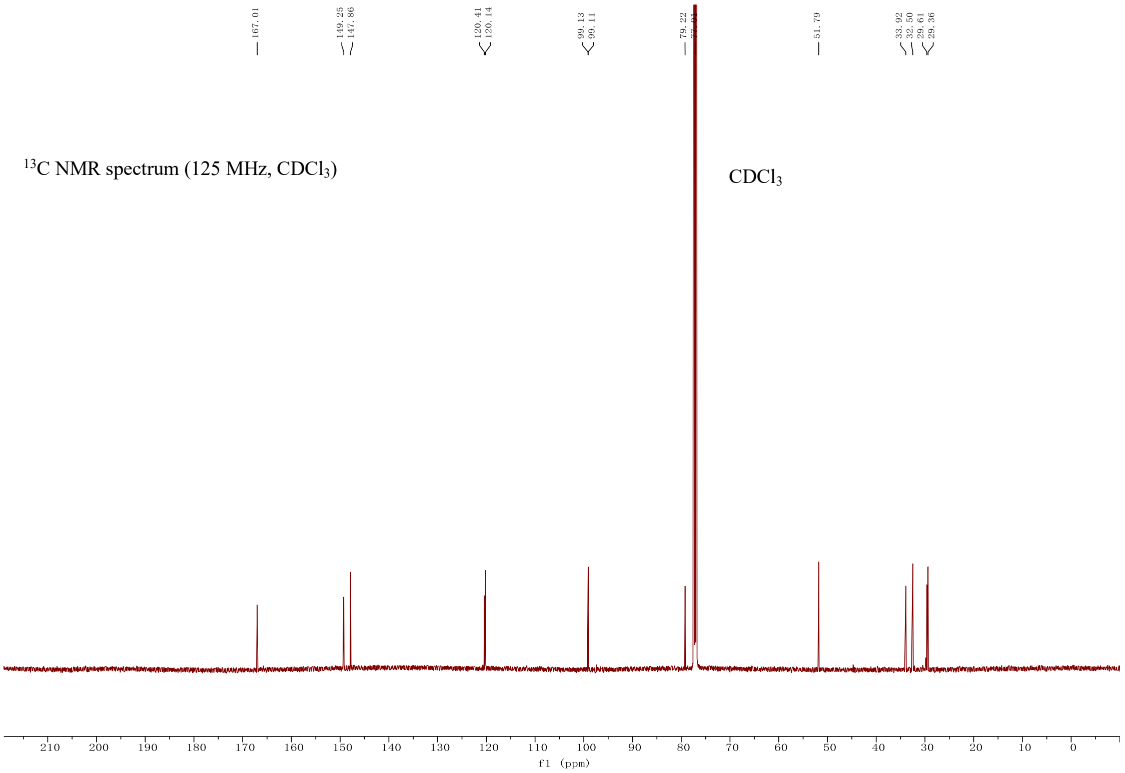

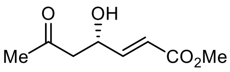

12

^{1}H NMR spectrum (500 MHz, $CDCl_3$)

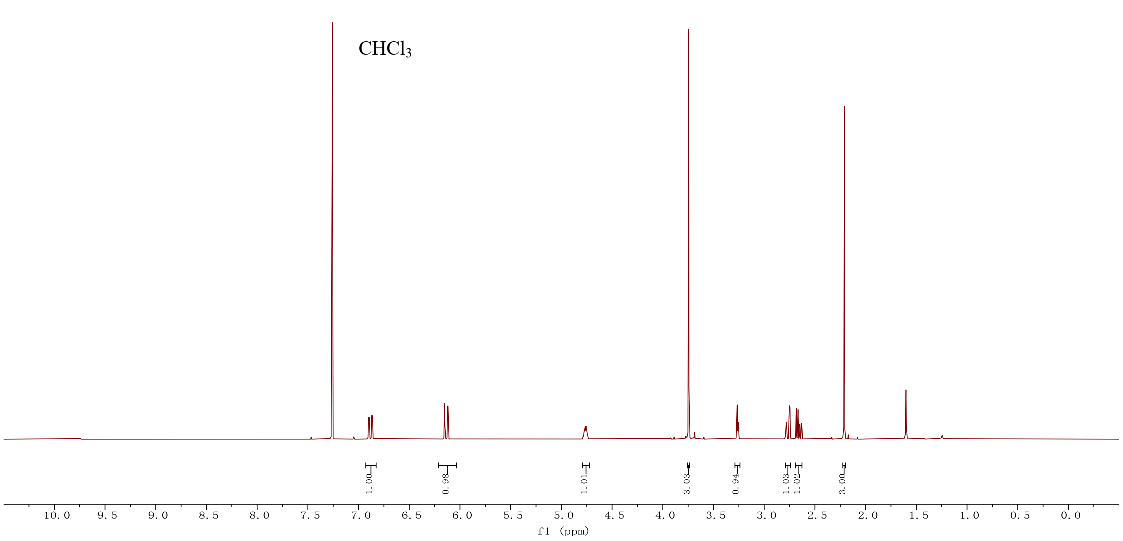

^{13}C NMR spectrum (125 MHz, $CDCl_3$)

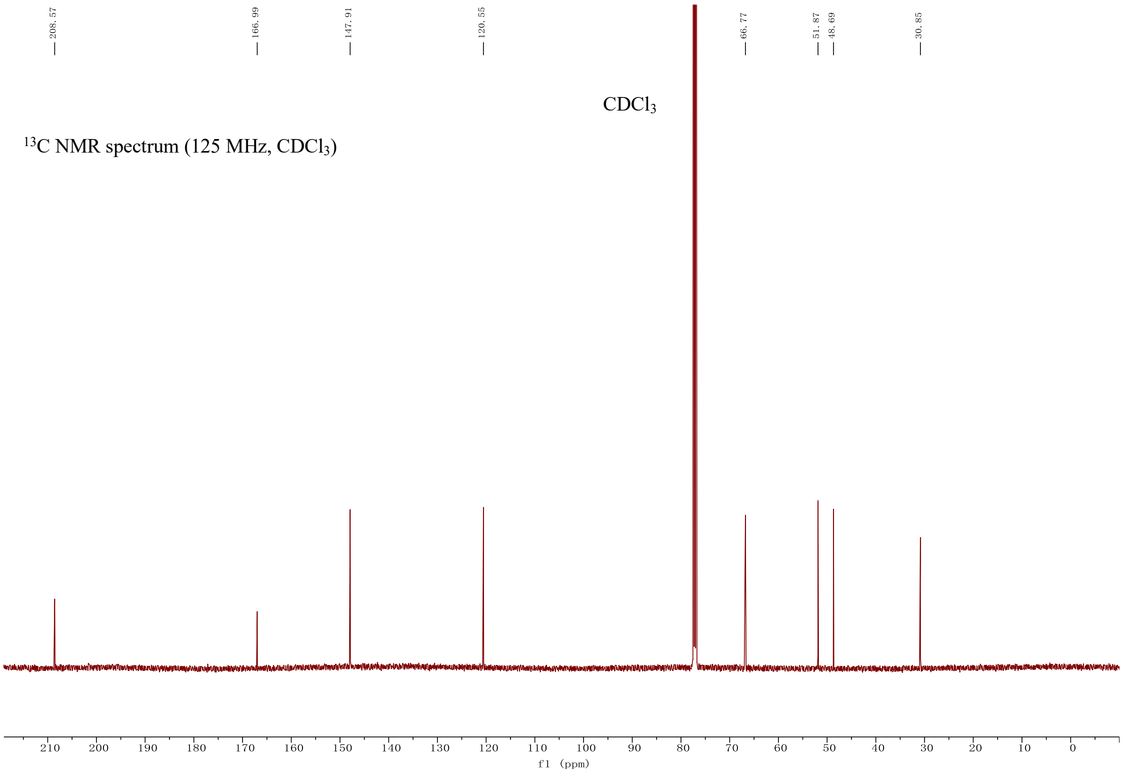

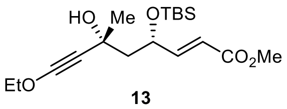

13

^{1}H NMR spectrum (600 MHz, $CDCl_3$)

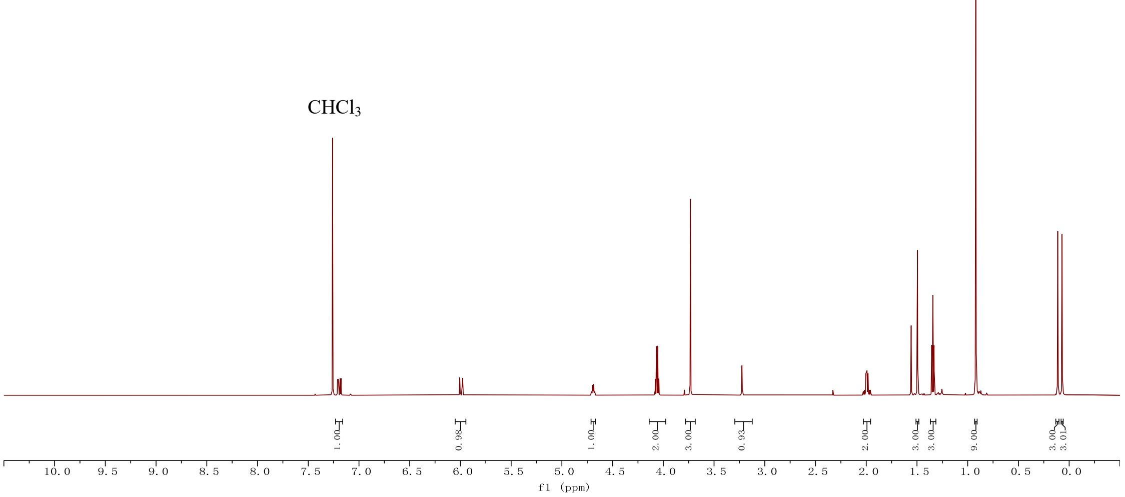

^{13}C NMR spectrum (150 MHz, $CDCl_3$)

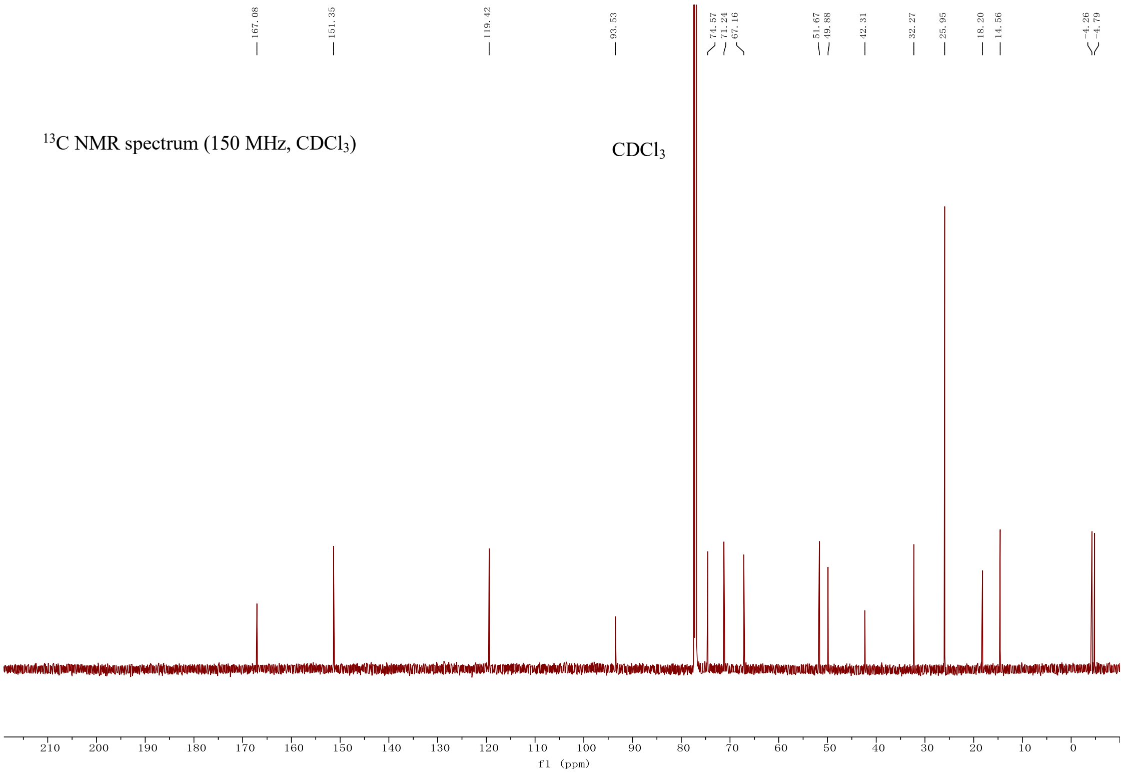

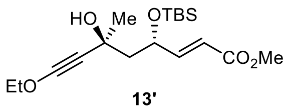

13'

^{1}H NMR spectrum (600 MHz, C_6D_6)

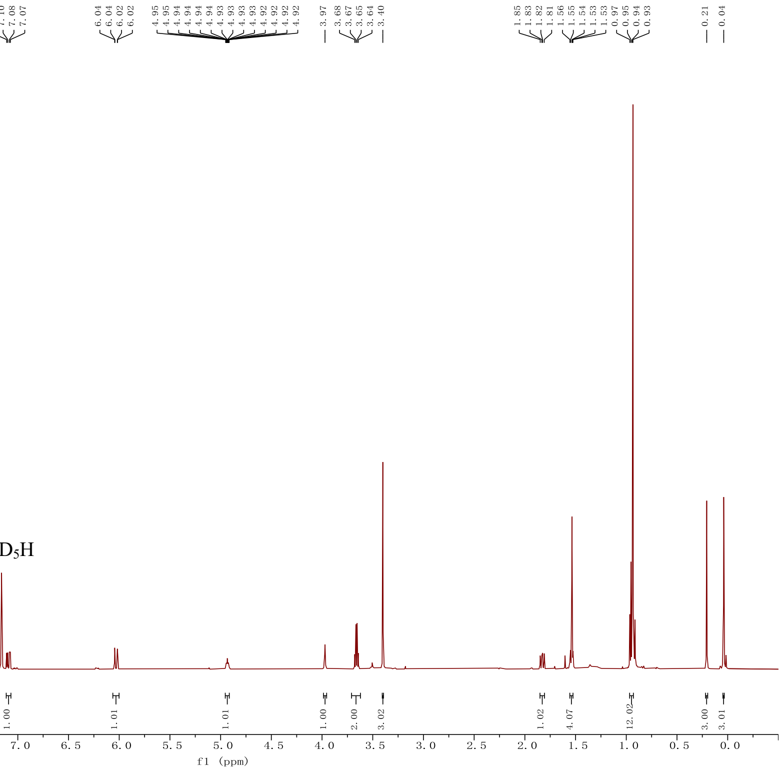

^{13}C NMR spectrum (150 MHz, C_6D_6)

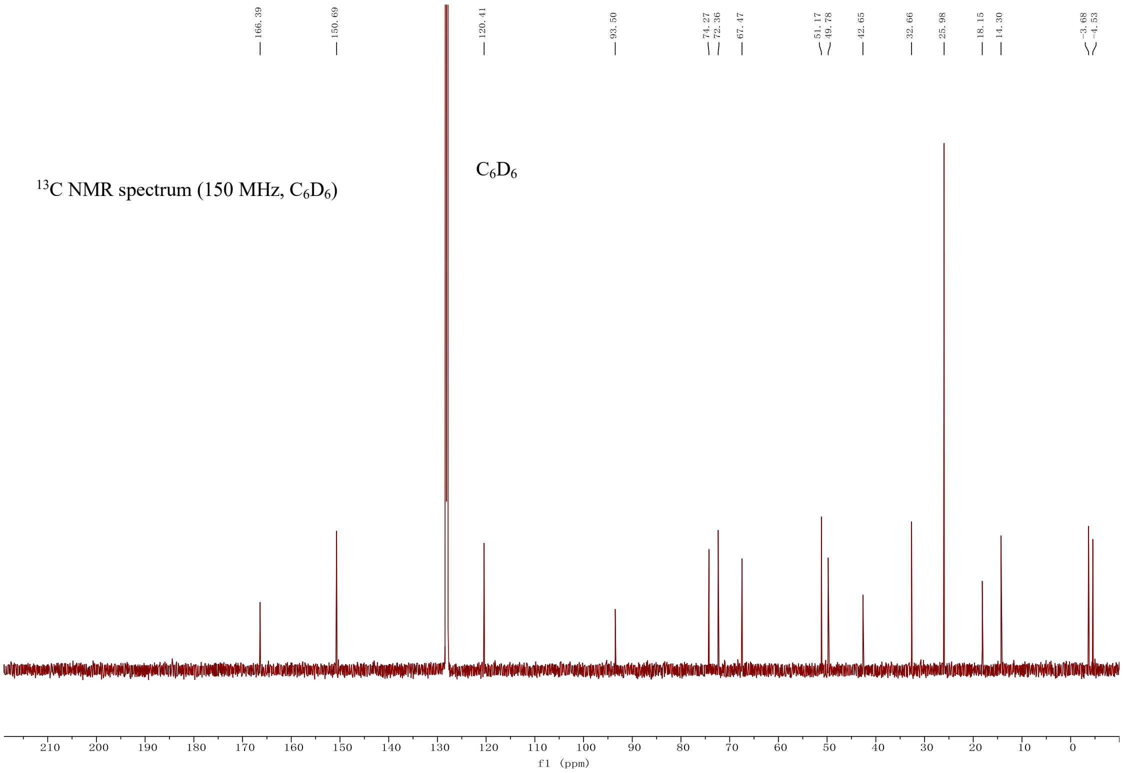

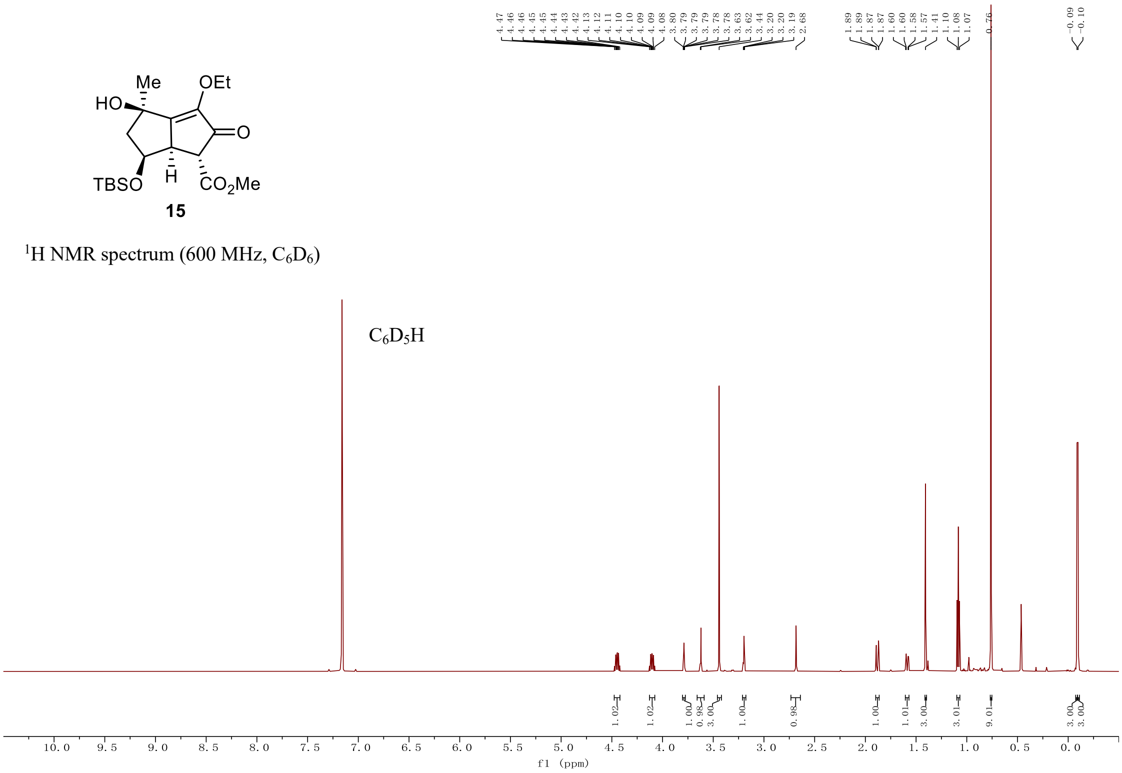

15

^1H NMR spectrum (600 MHz, C_6D_6)

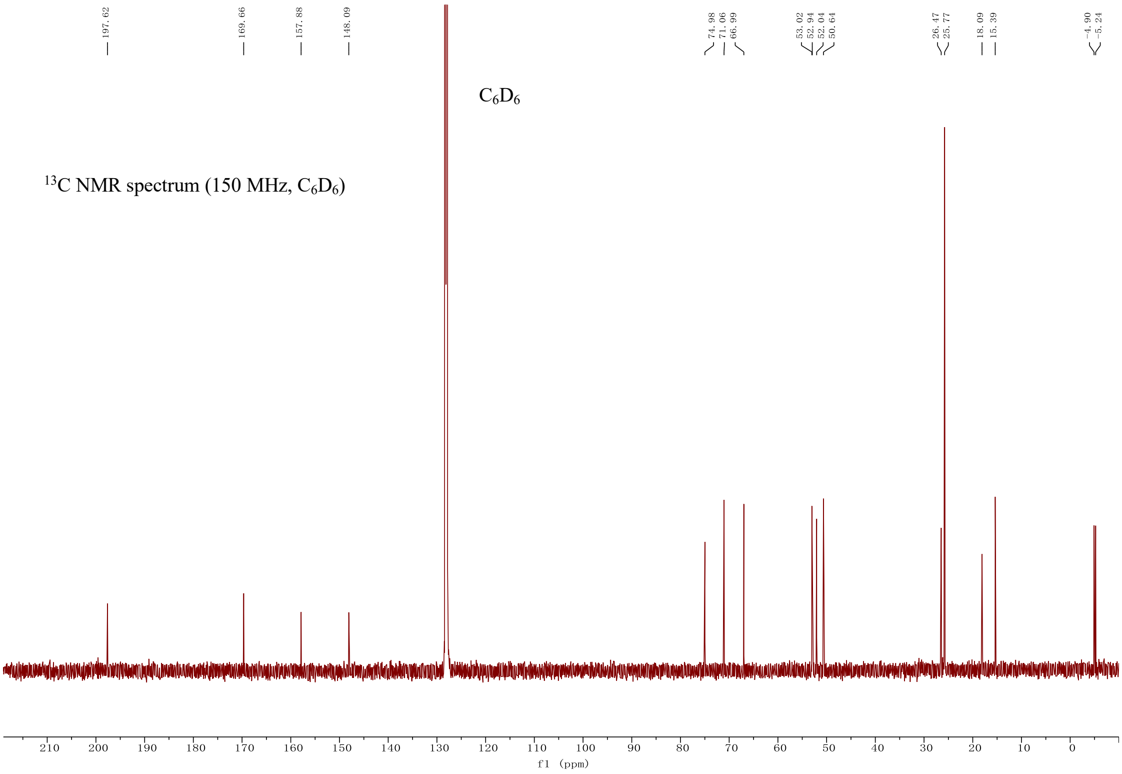

^{13}C NMR spectrum (150 MHz, C_6D_6)

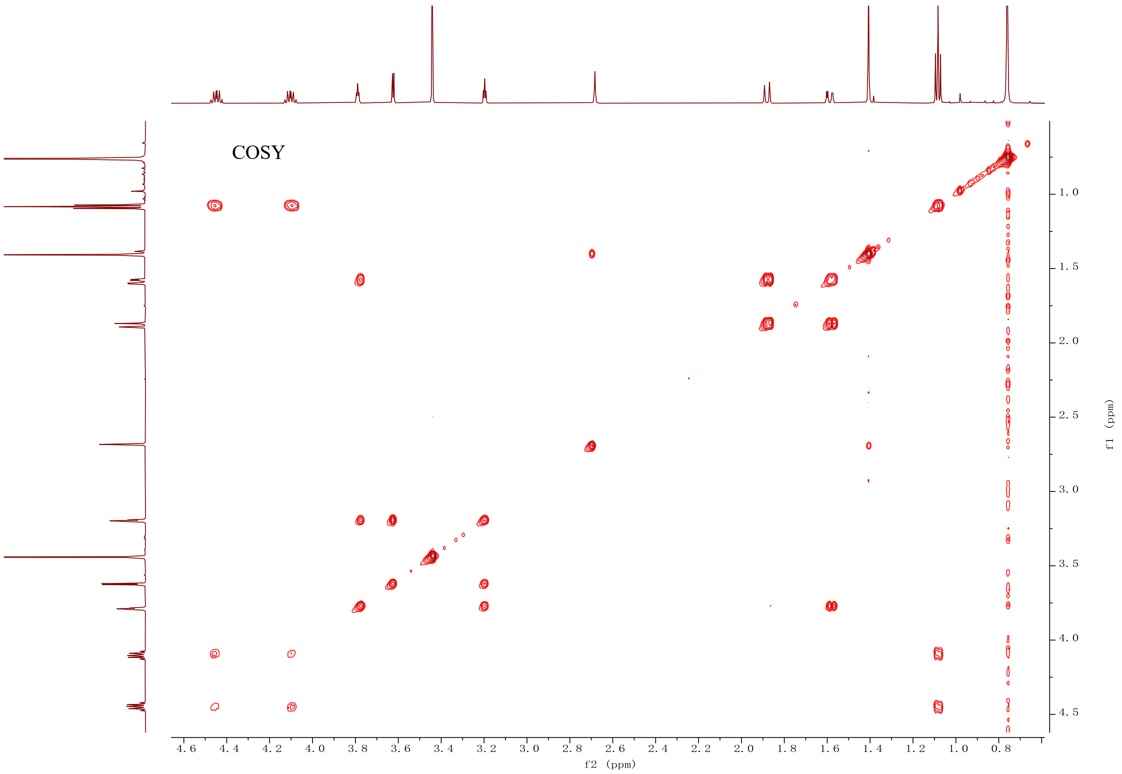
COSY
f2 (ppm)
f1 (ppm)

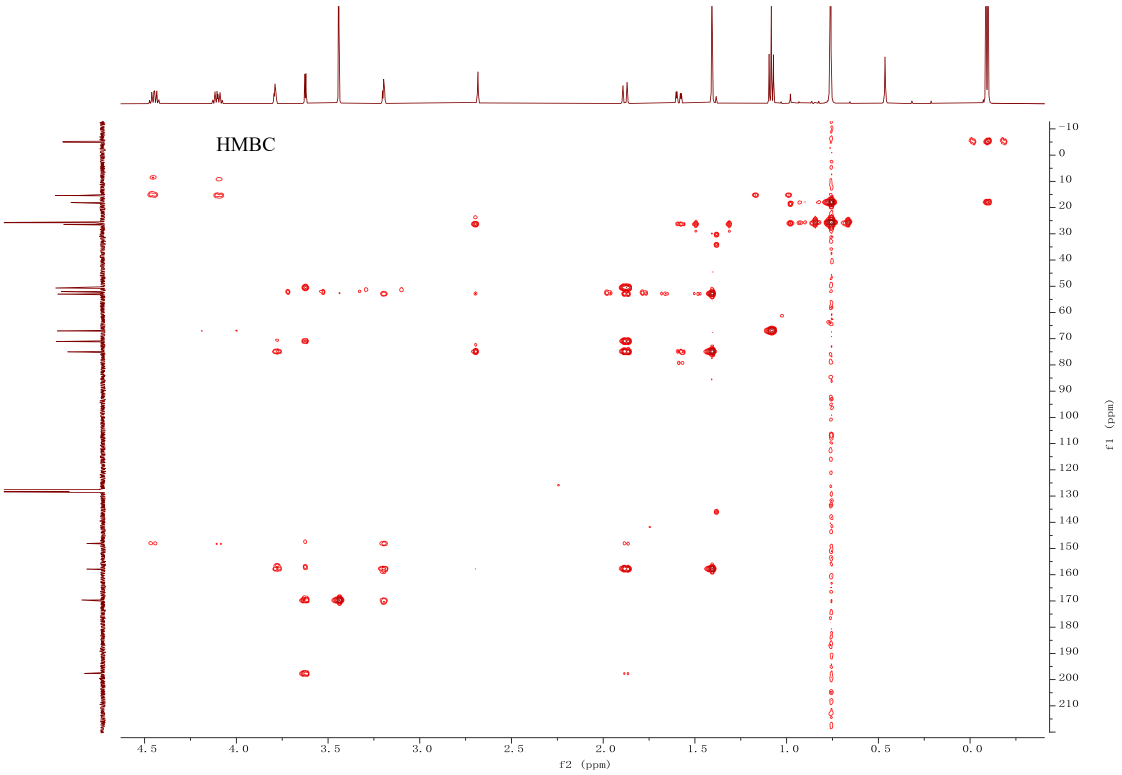
HMBC
f2 (ppm)
f1 (ppm)

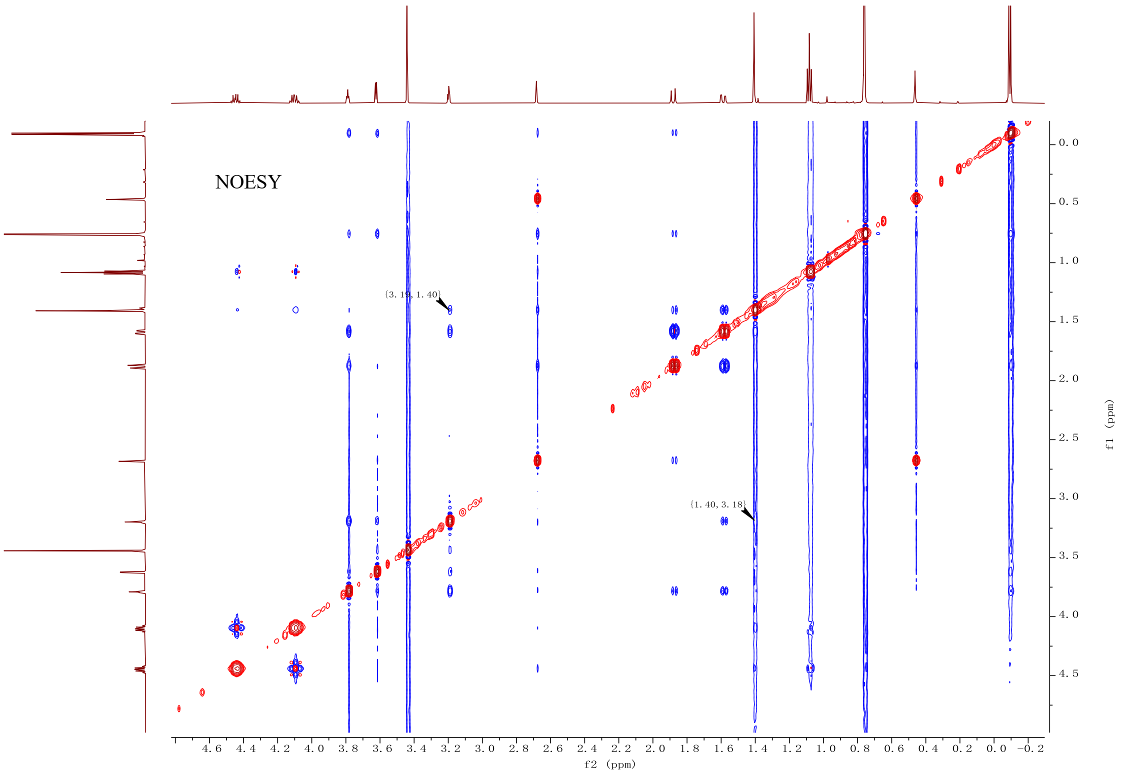
NOESY
(3.19, 1.40)
(1.40, 3.18)
f2 (ppm)
f1 (ppm)

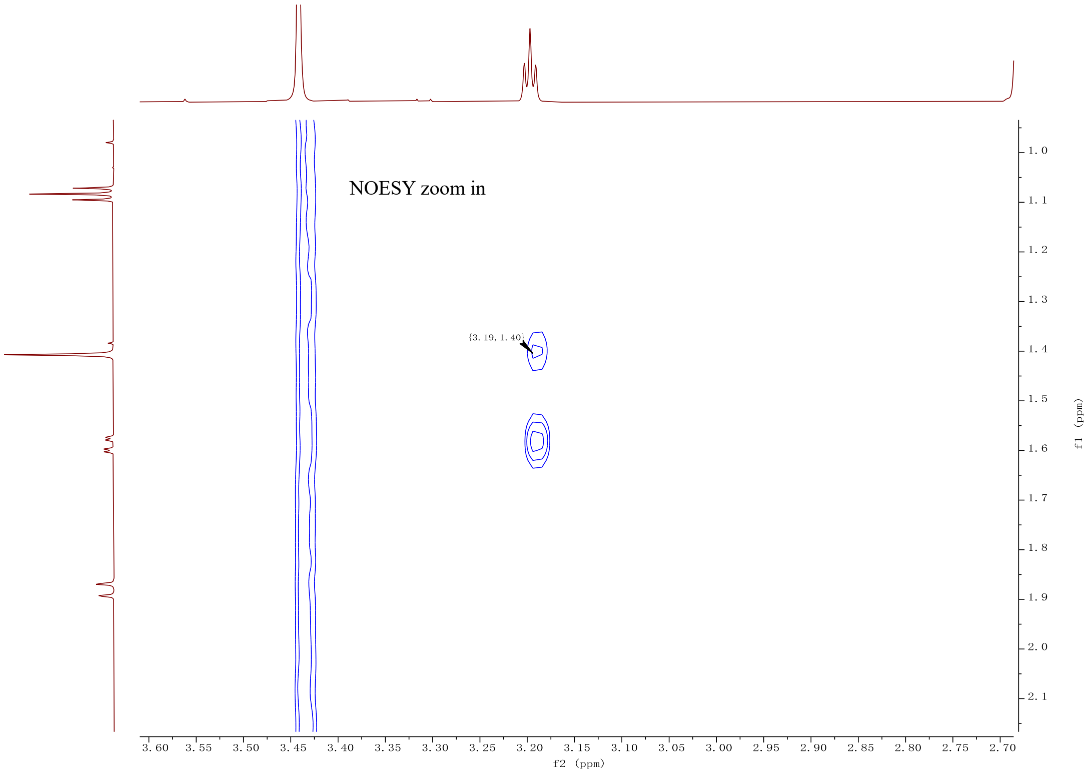
NOESY zoom in
(3.19, 1.40)
f2 (ppm)
f1 (ppm)

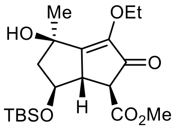

15'

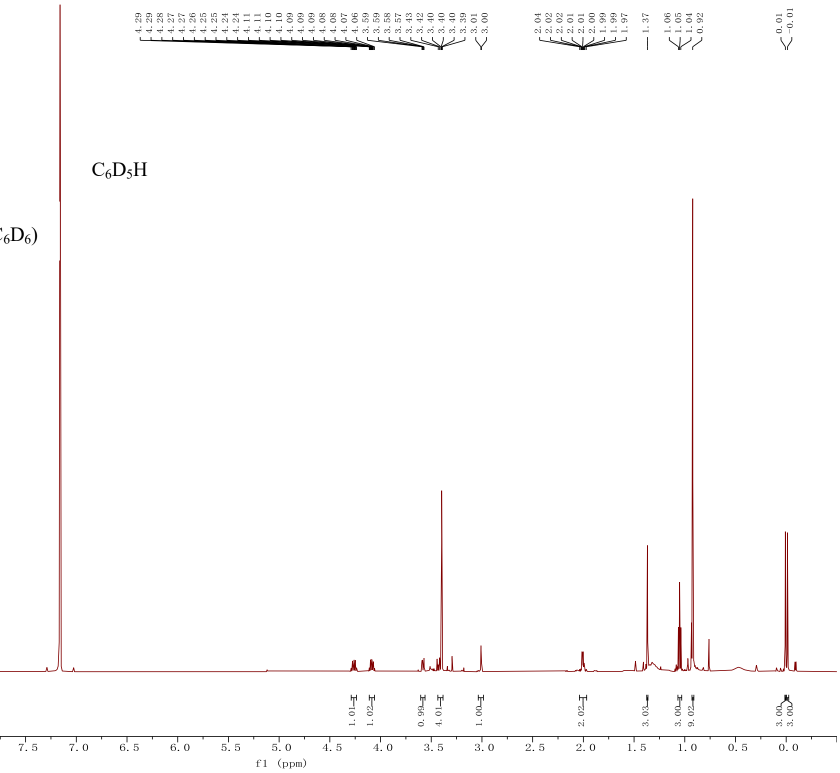

^{1}H NMR spectrum (600 MHz, C_6D_6)

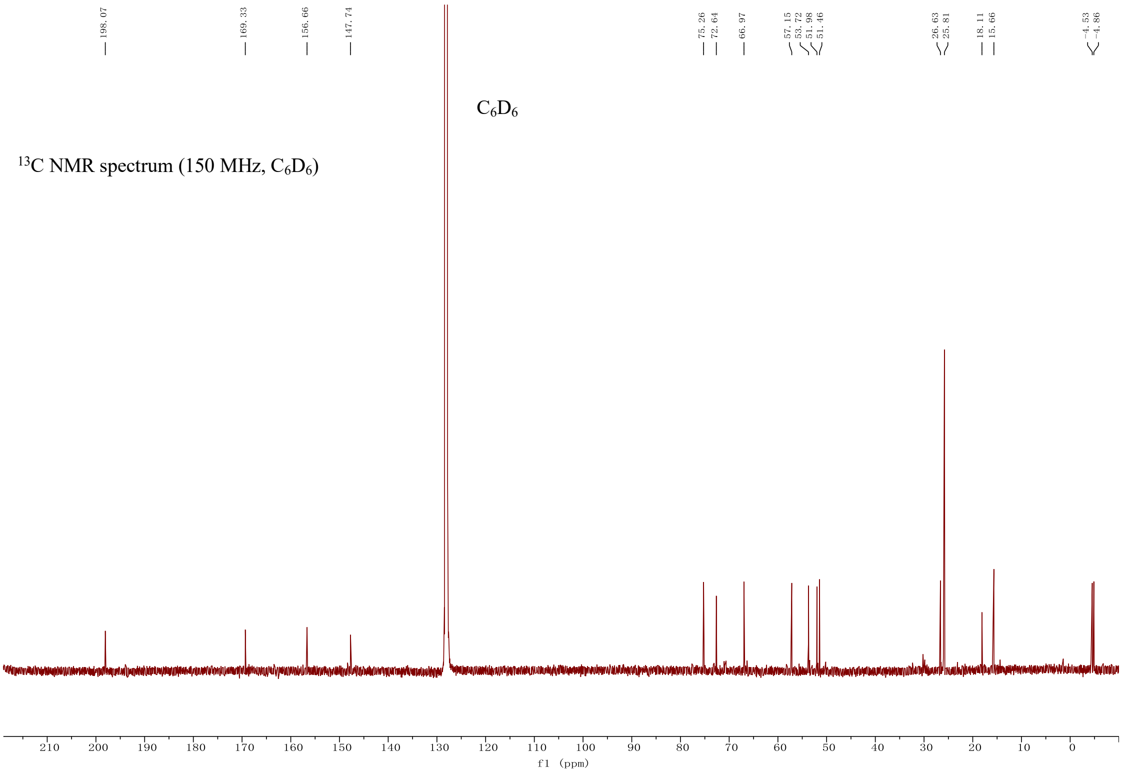

^{13}C NMR spectrum (150 MHz, C_6D_6)

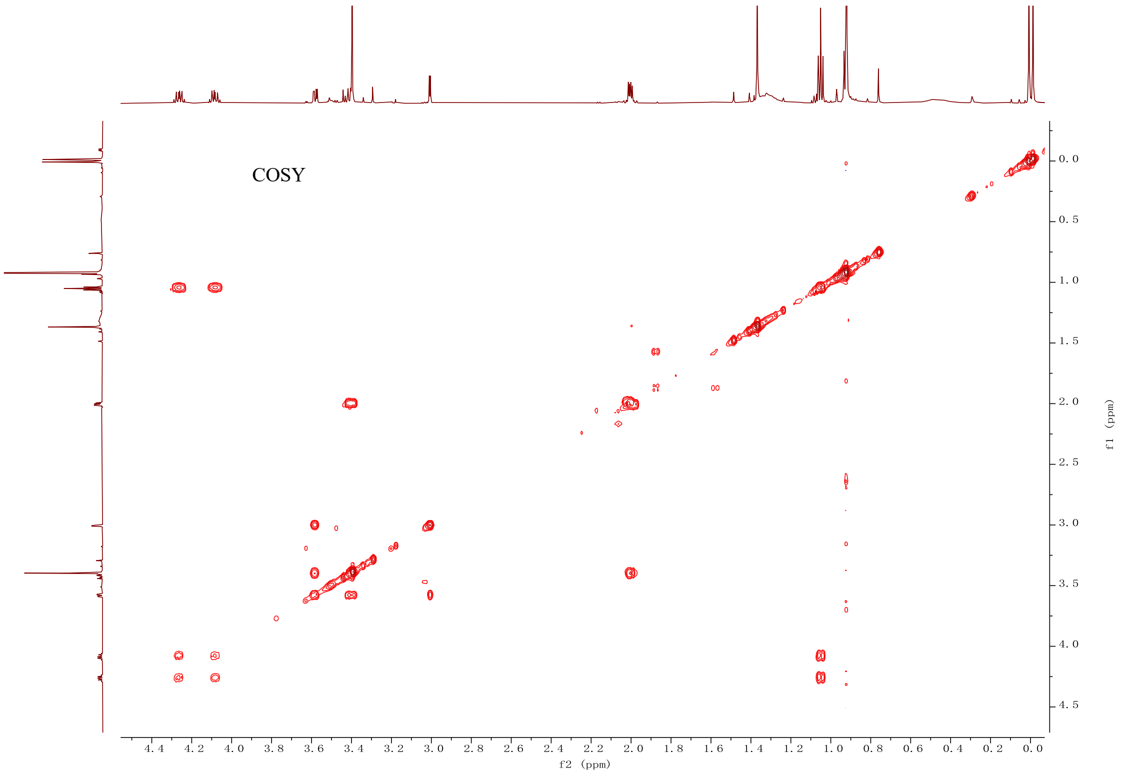
COSY
f2 (ppm)
f1 (ppm)

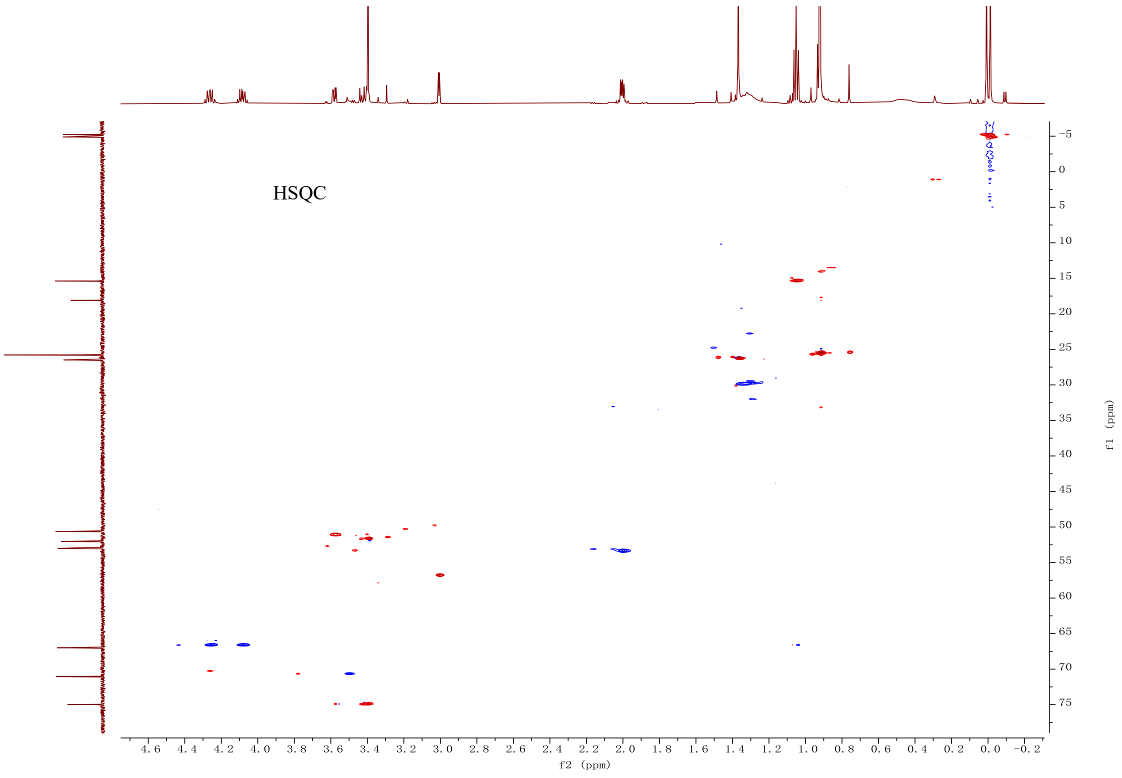
HSQC
f2 (ppm)
f1 (ppm)

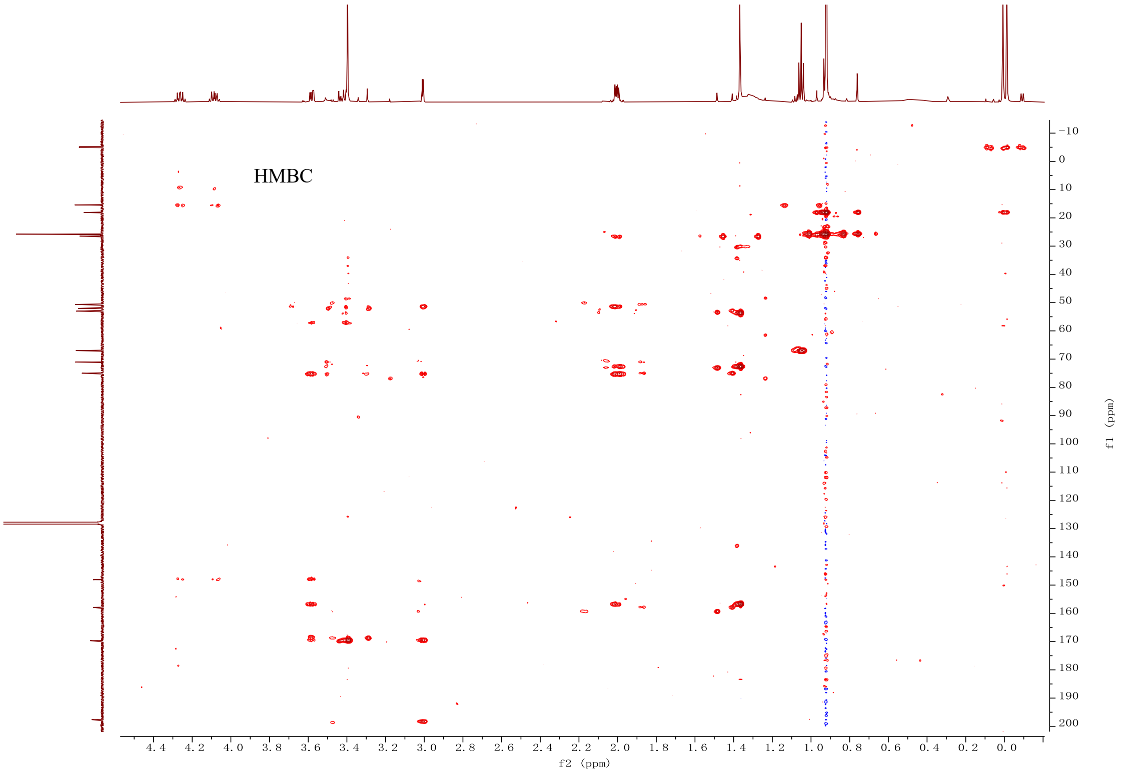

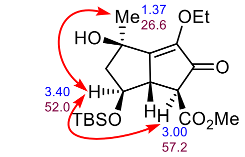

15'

selected key NOESY correlations

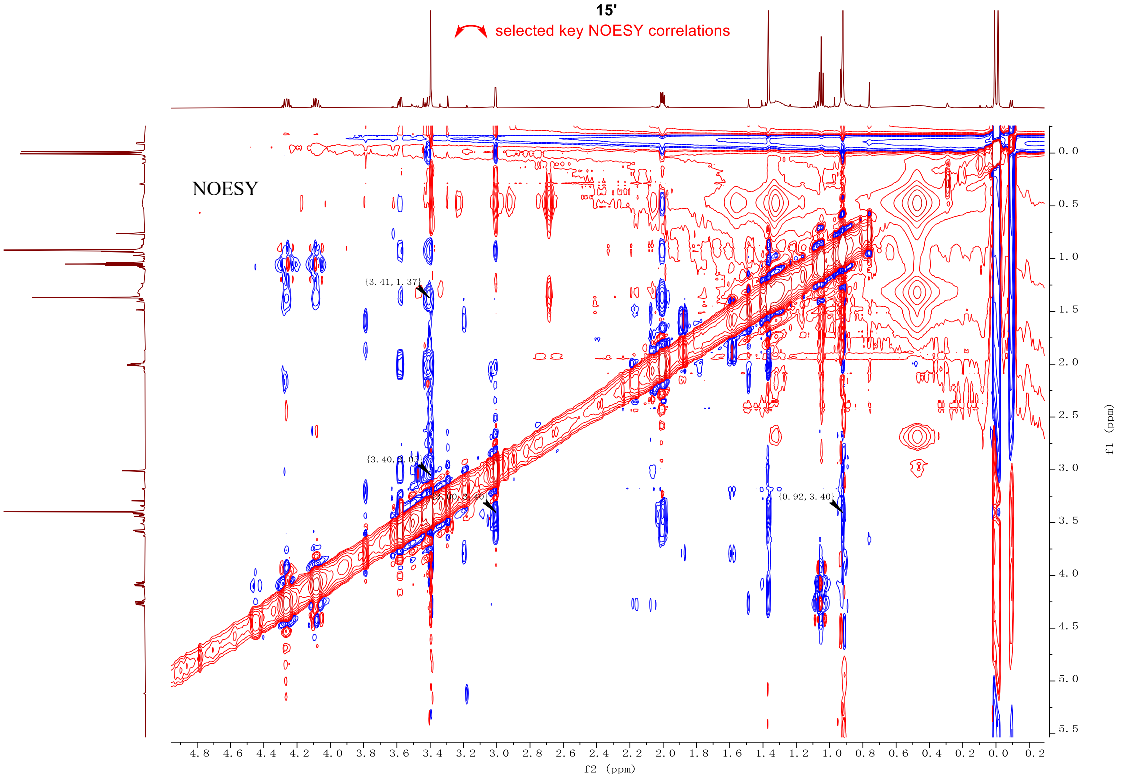

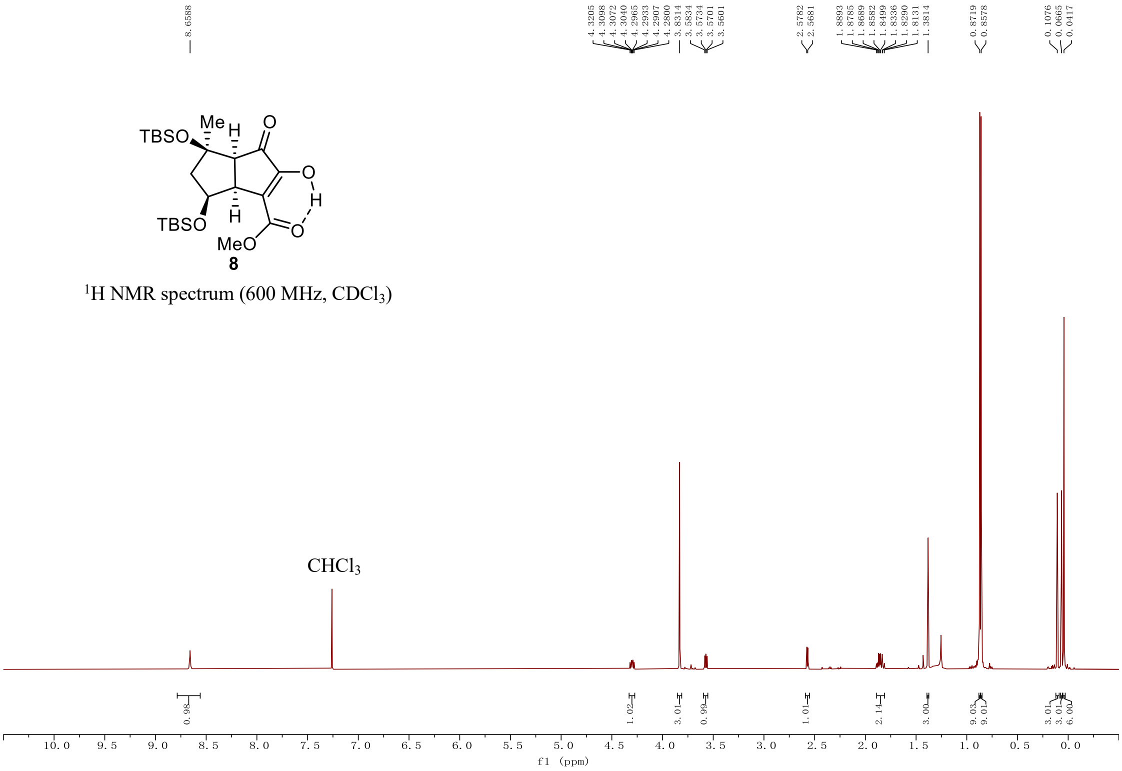
8.6588
4.3205
4.3098
4.3072
4.3040
4.2965
4.2933
4.2907
4.2800
3.8314
3.5834
3.5734
3.5701
3.5601
2.5782
2.5681
1.8893
1.8785
1.8689
1.8582
1.8499
1.8336
1.8290
1.8131
1.3814
0.8719
0.8578
0.1076
0.0665
0.0417
TBSO
Me
H
O
O
H
TBSO
H
MeO
O
8
^{1}H NMR spectrum (600 MHz, $CDCl_3$)
$CHCl_3$
0.98
1.02
3.01
0.99
1.01
2.14
3.00
9.03
9.01
3.01
3.01
6.00
f1 (ppm)

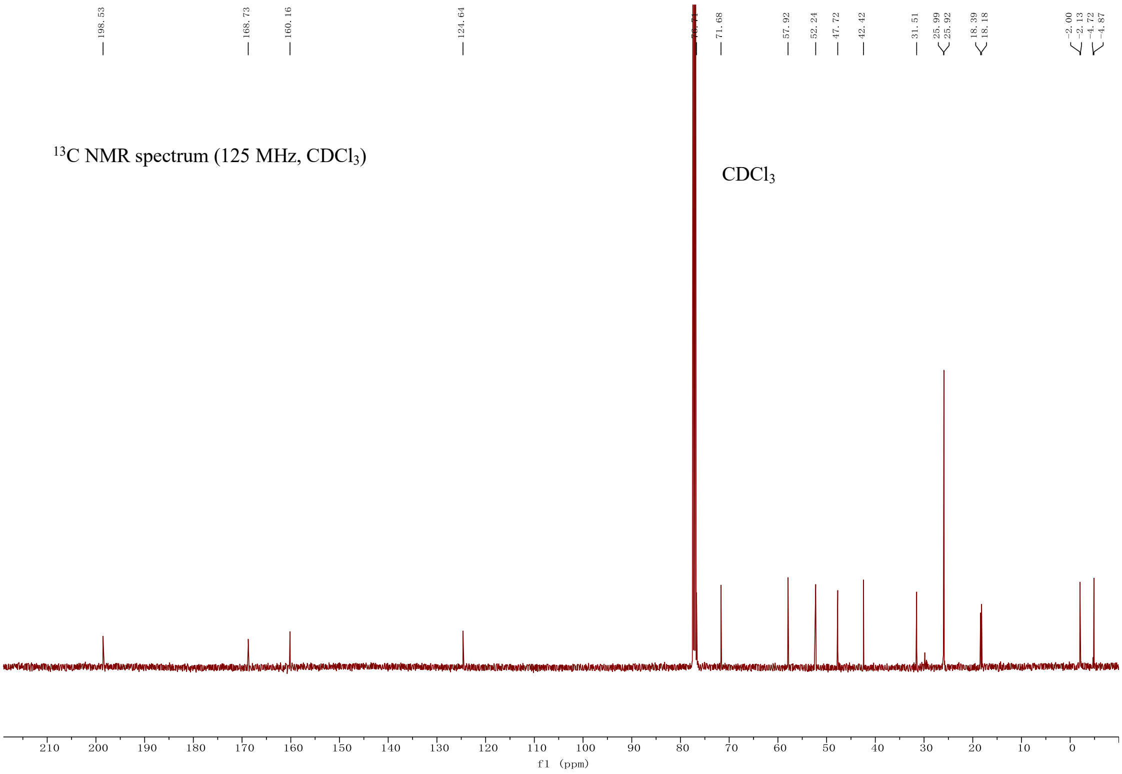
198.53
168.73
160.16
124.64
71.68
57.92
52.24
47.72
42.42
31.51
25.99
25.92
18.39
18.18
-2.00
-2.13
-4.72
-4.87
^{13}C NMR spectrum (125 MHz, $CDCl_3$)
$CDCl_3$
f1 (ppm)

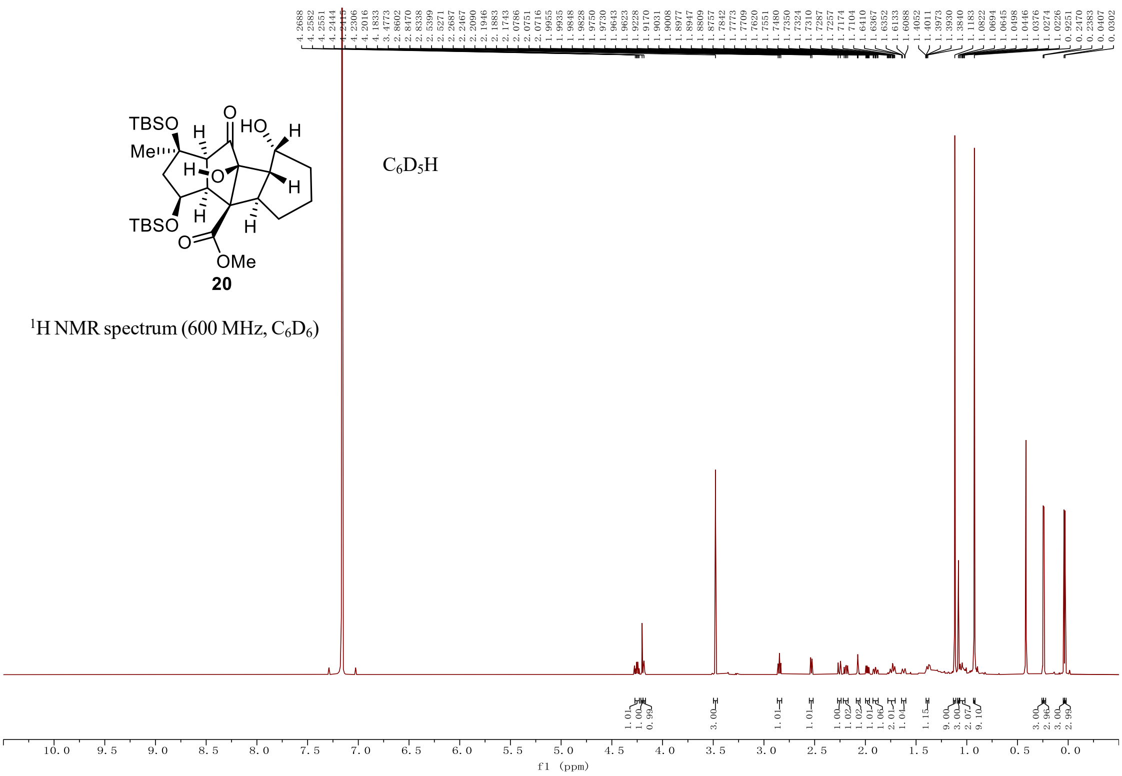

¹H NMR spectrum (600 MHz, C_6D_6)

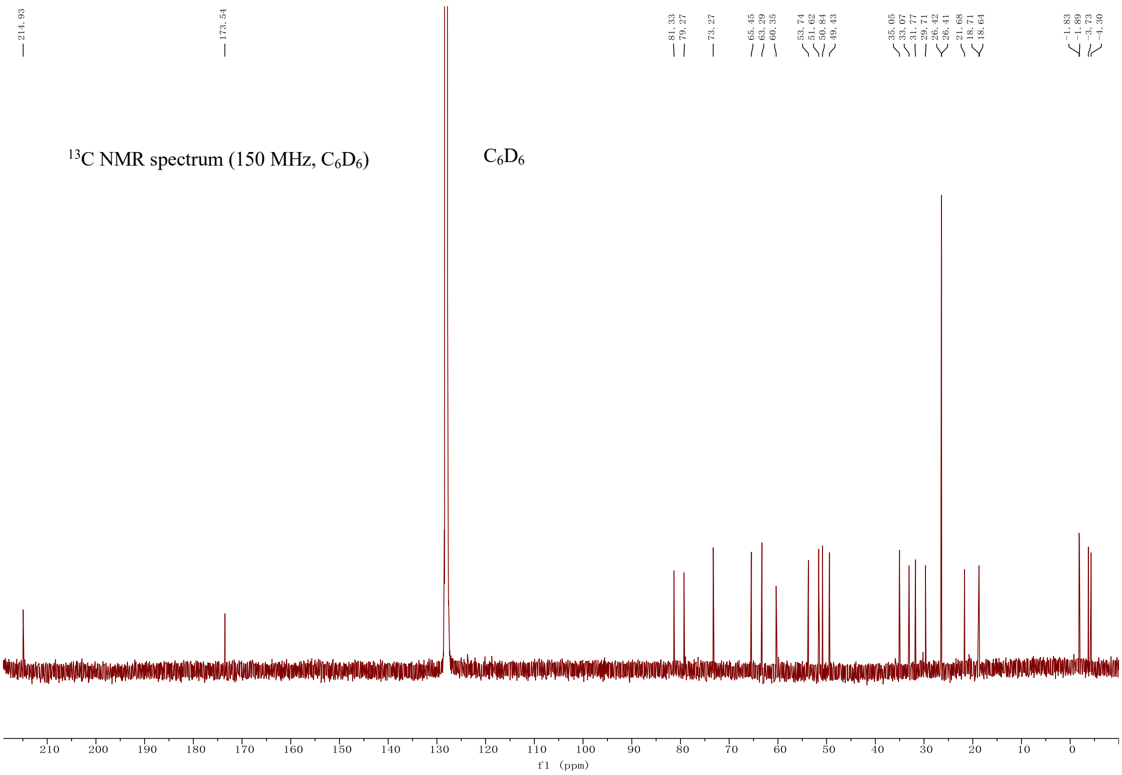

¹³C NMR spectrum (150 MHz, C_6D_6)

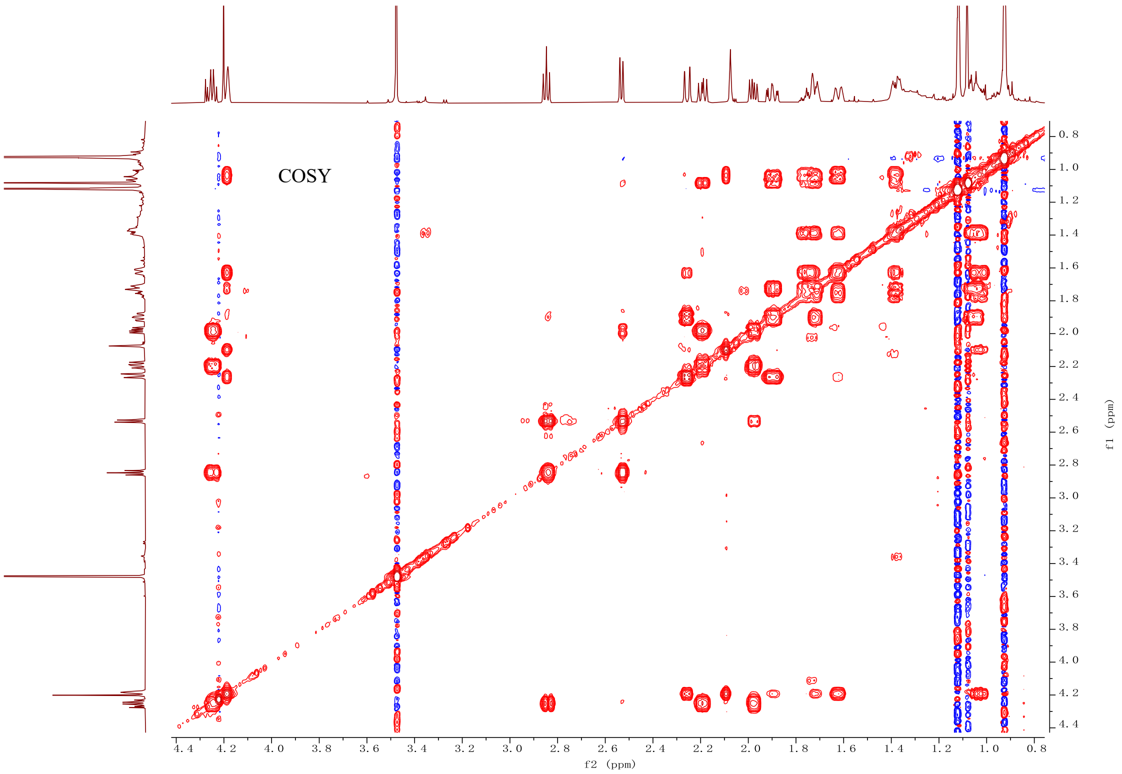
COSY
f2 (ppm)
f1 (ppm)

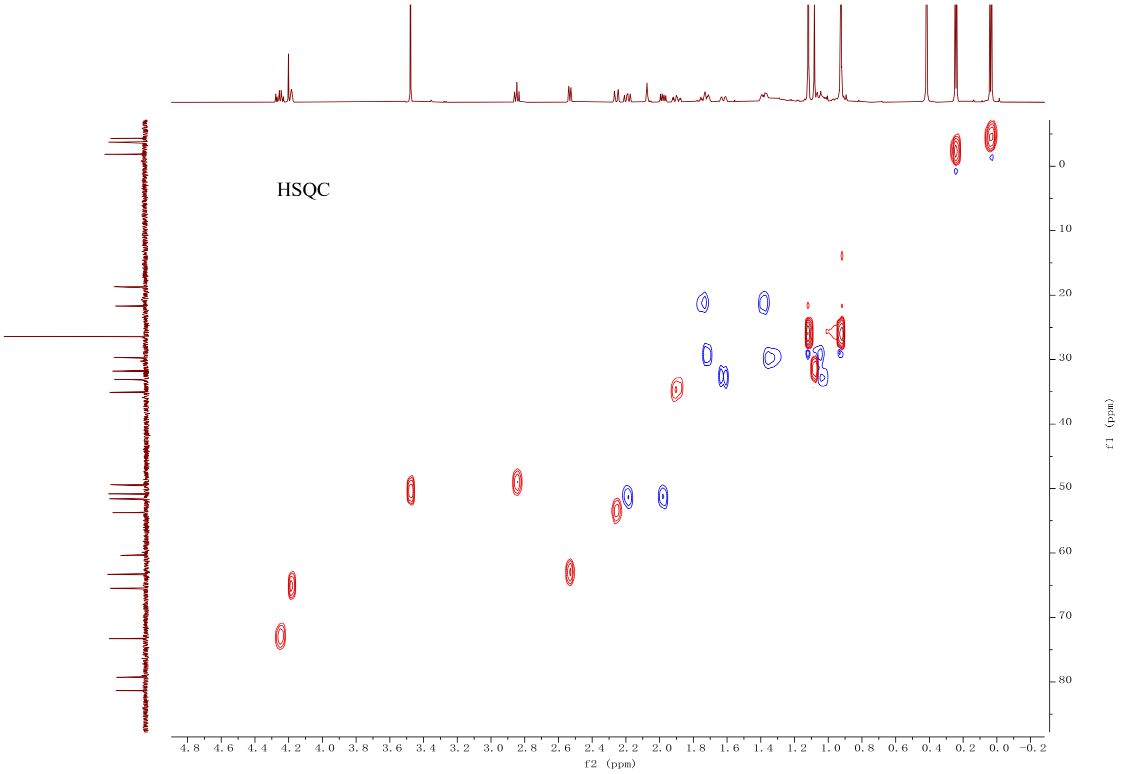
HSQC
f2 (ppm)
f1 (ppm)

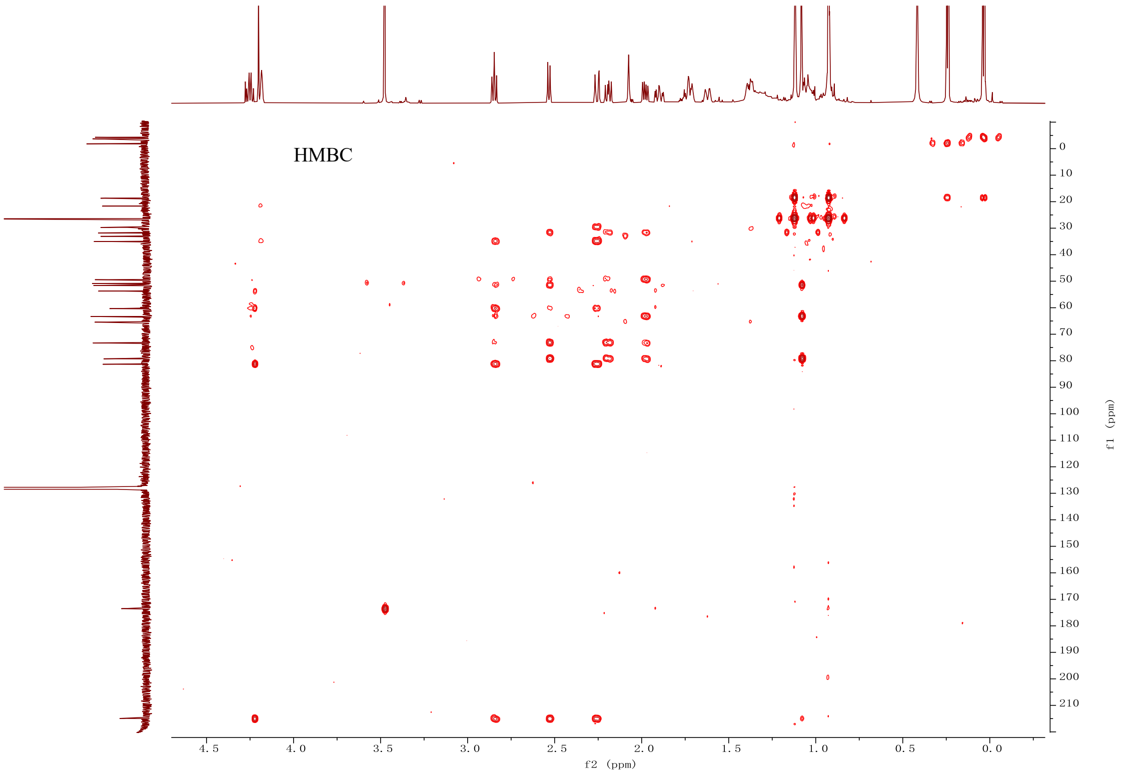
HMBC
f2 (ppm)
f1 (ppm)

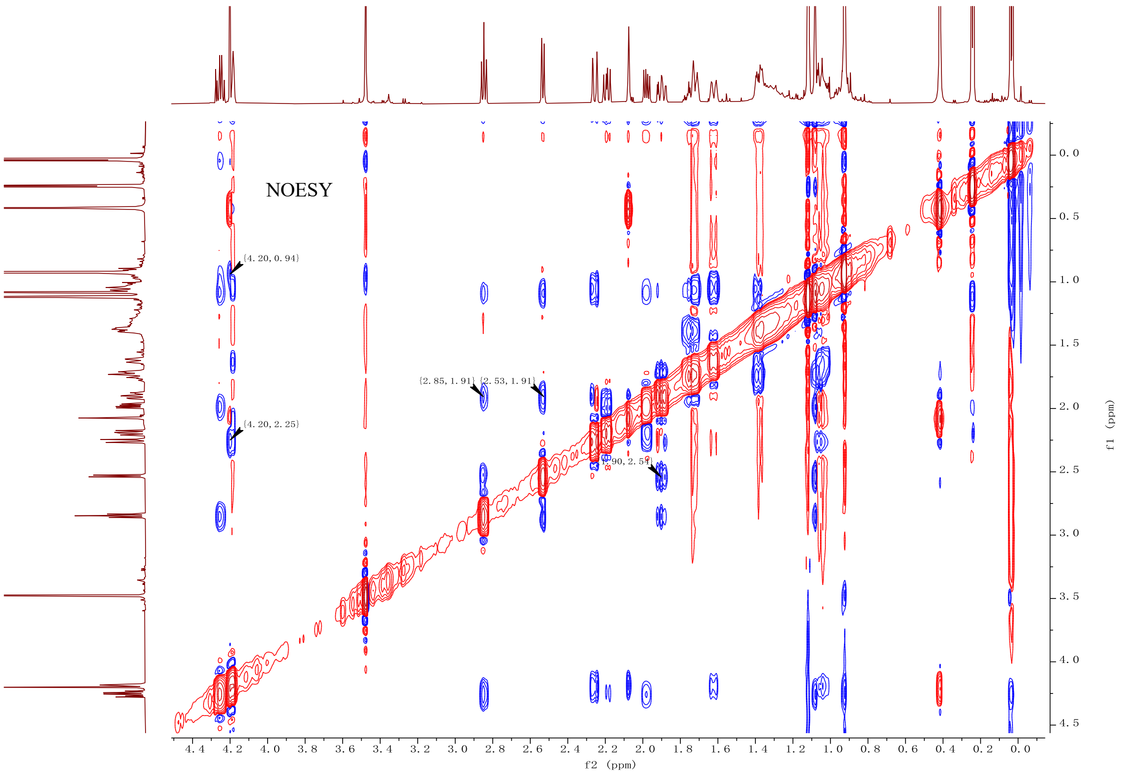
NOESY
(4.20, 0.94)
(4.20, 2.25)
(2.85, 1.91)
(2.53, 1.91)
f2 (ppm)
f1 (ppm)

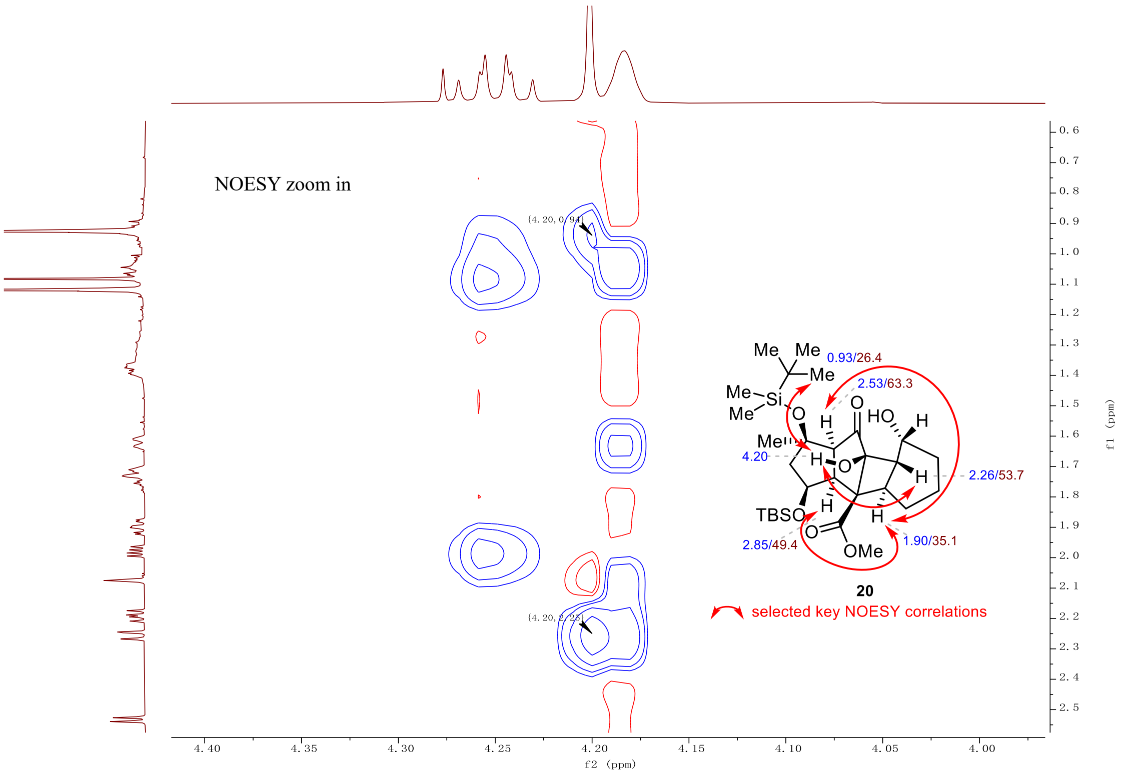
NOESY zoom in
0.93/26.4
2.53/63.3
4.20
2.26/53.7
2.85/49.4
1.90/35.1
20
selected key NOESY correlations
f2 (ppm)
f1 (ppm)

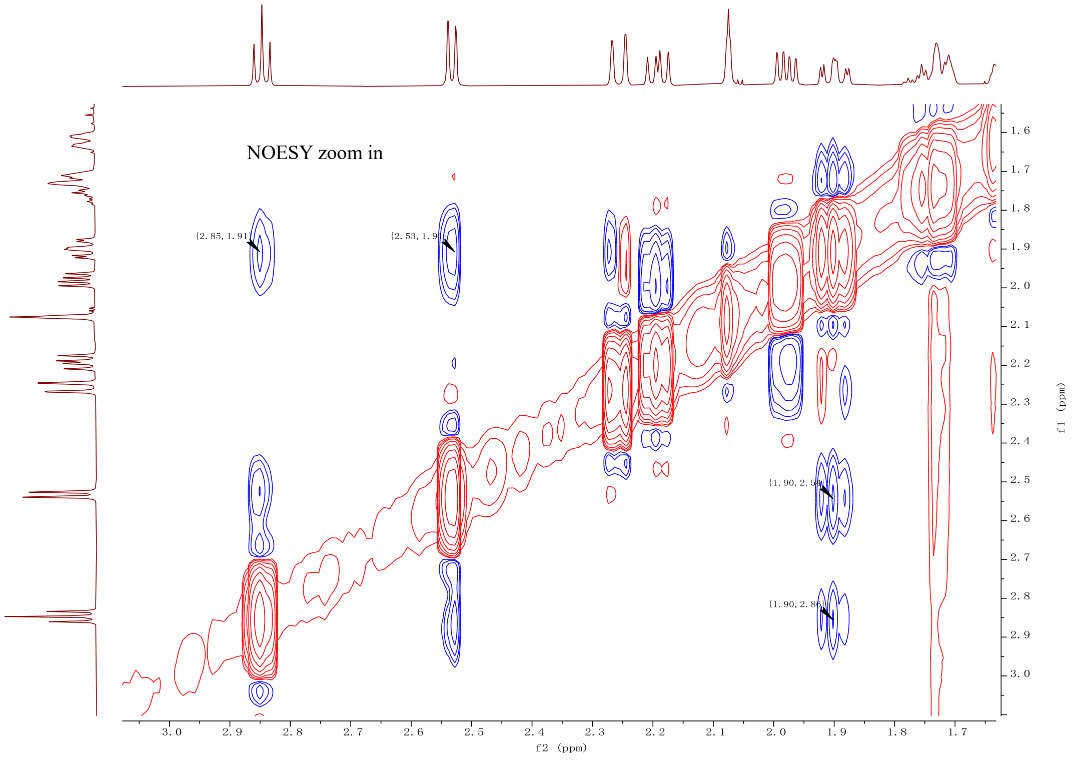
NOESY zoom in
f2 (ppm)
f1 (ppm)

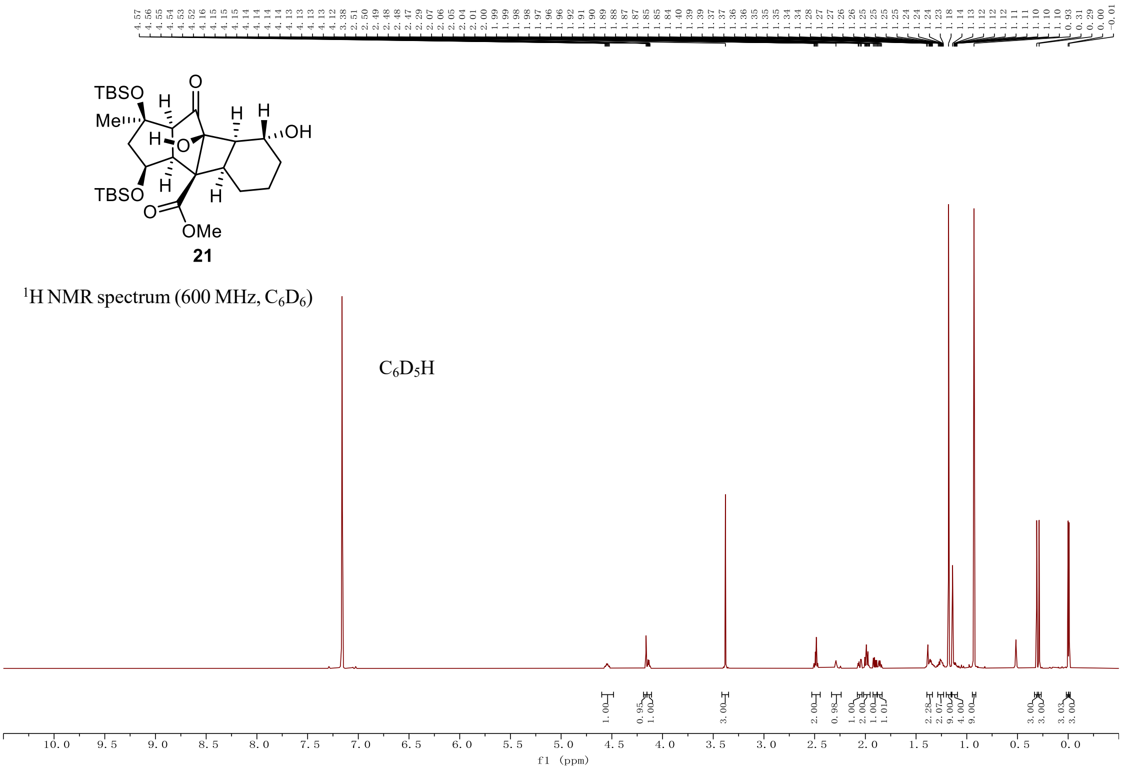

^{1}H NMR spectrum (600 MHz, C_6D_6)

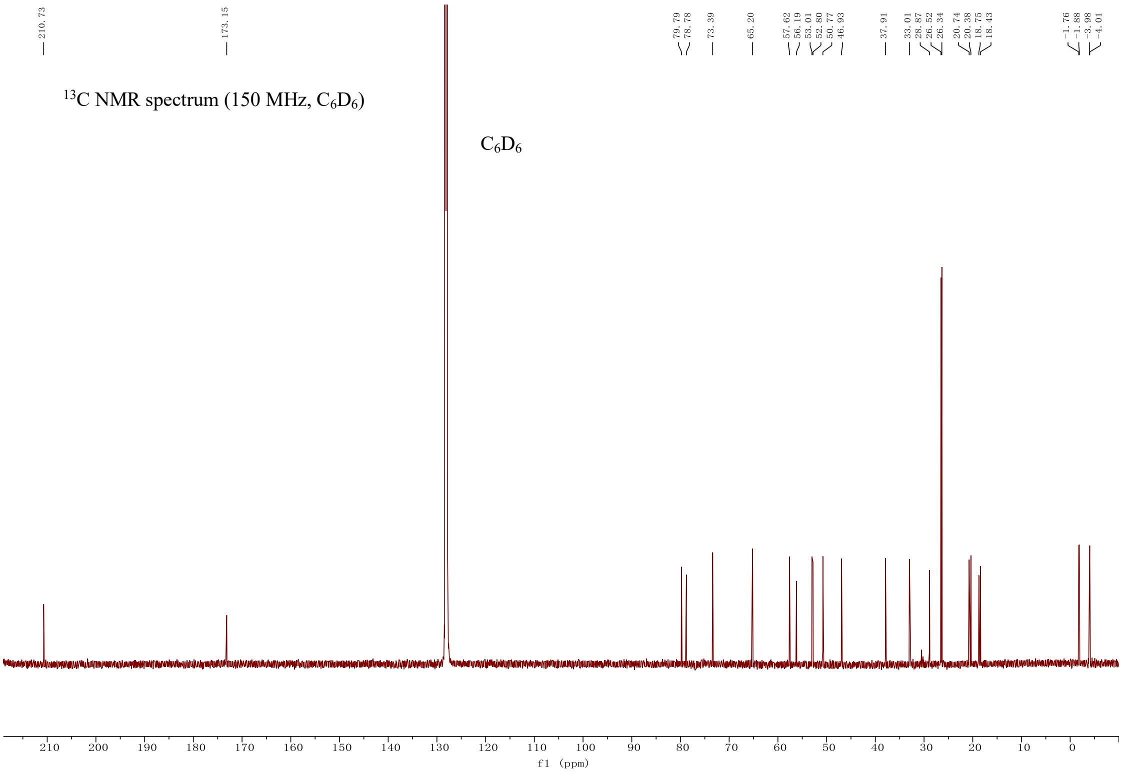

^{13}C NMR spectrum (150 MHz, C_6D_6)

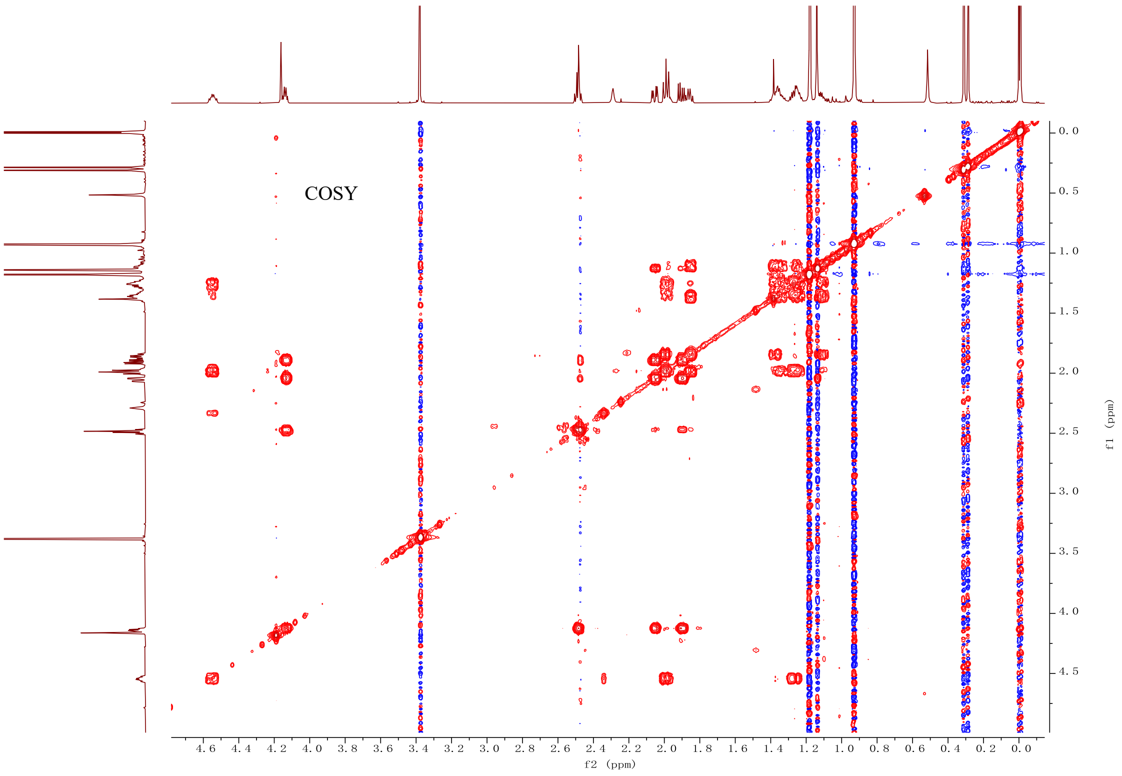
COSY
f2 (ppm)
f1 (ppm)

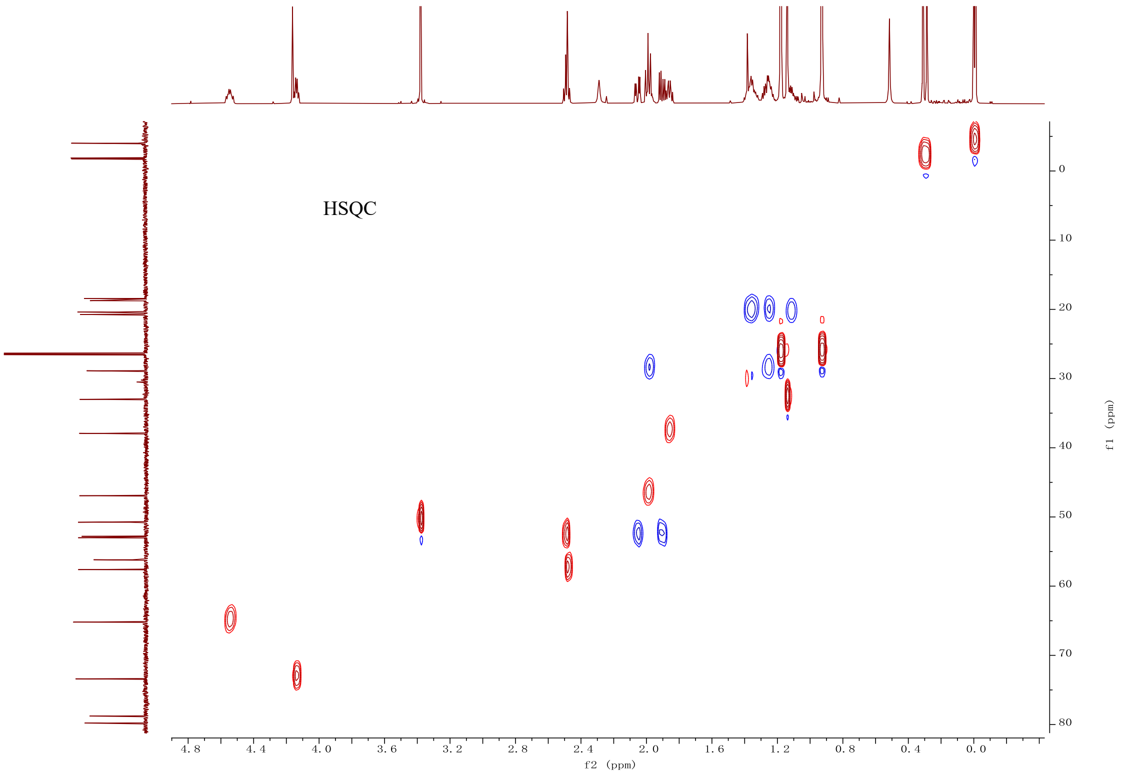
HSQC
f2 (ppm)
f1 (ppm)

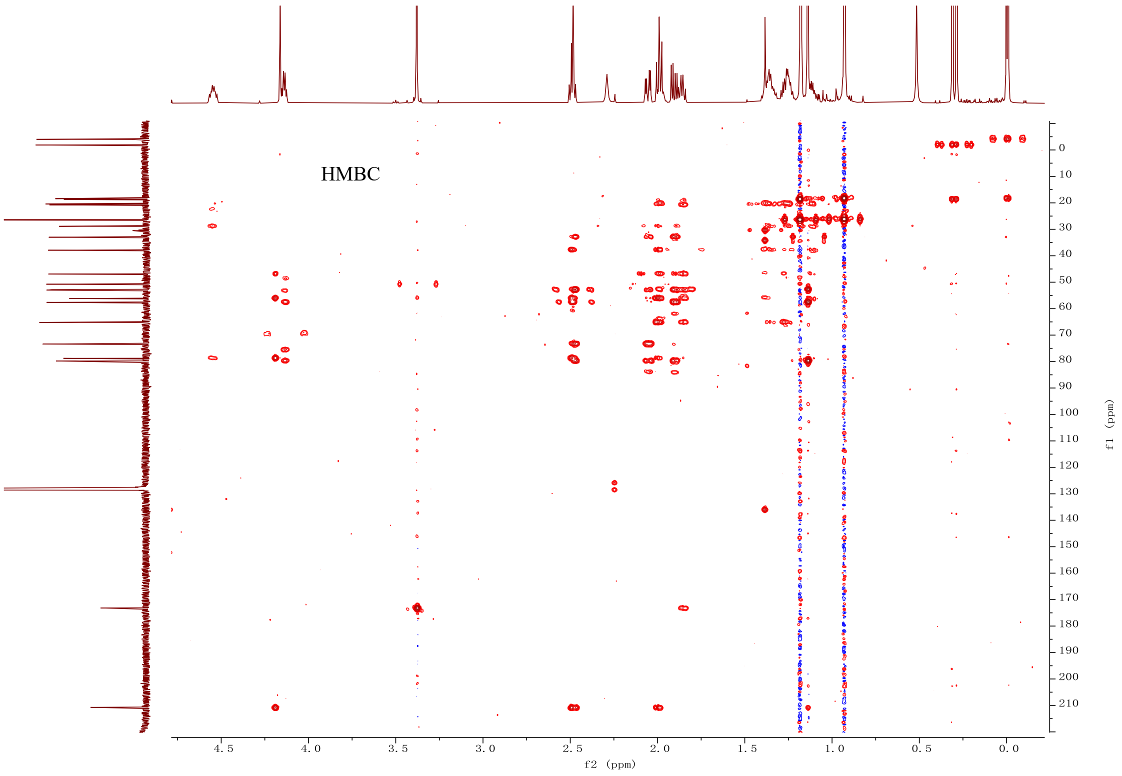
HMBC
f2 (ppm)
f1 (ppm)

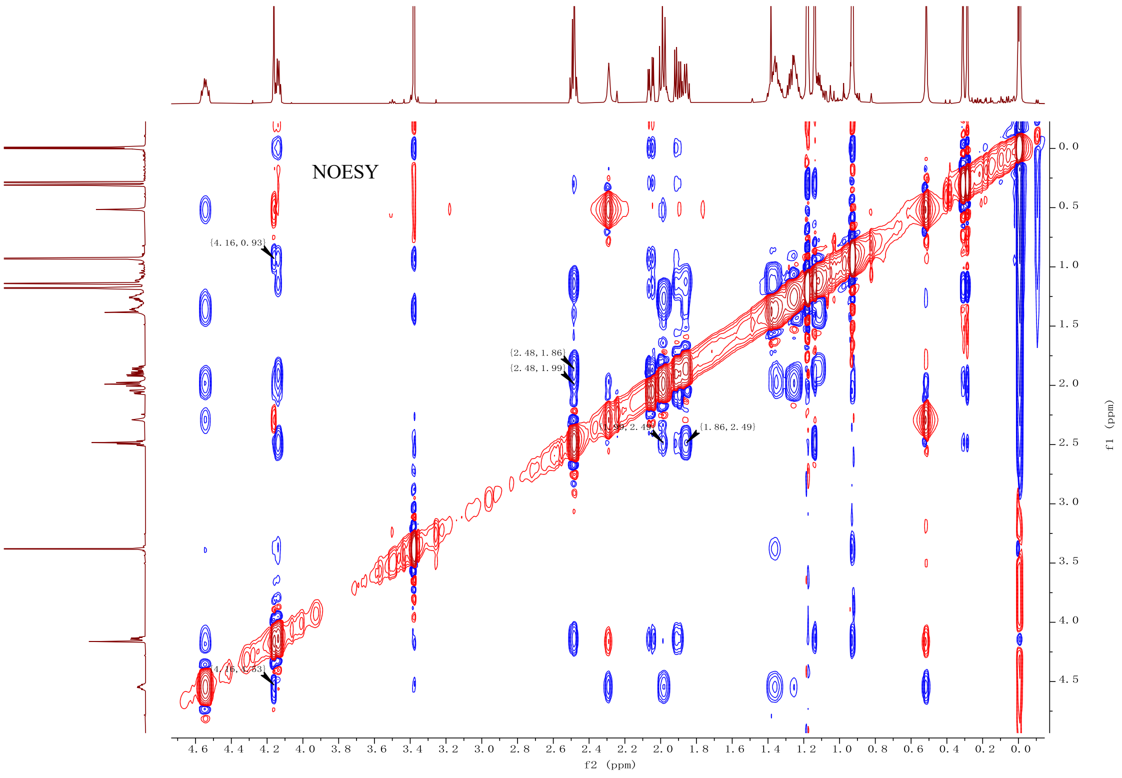
NOESY
(4.16, 0.93)
(2.48, 1.86)
(2.48, 1.99)
(1.86, 2.49)
f2 (ppm)
f1 (ppm)

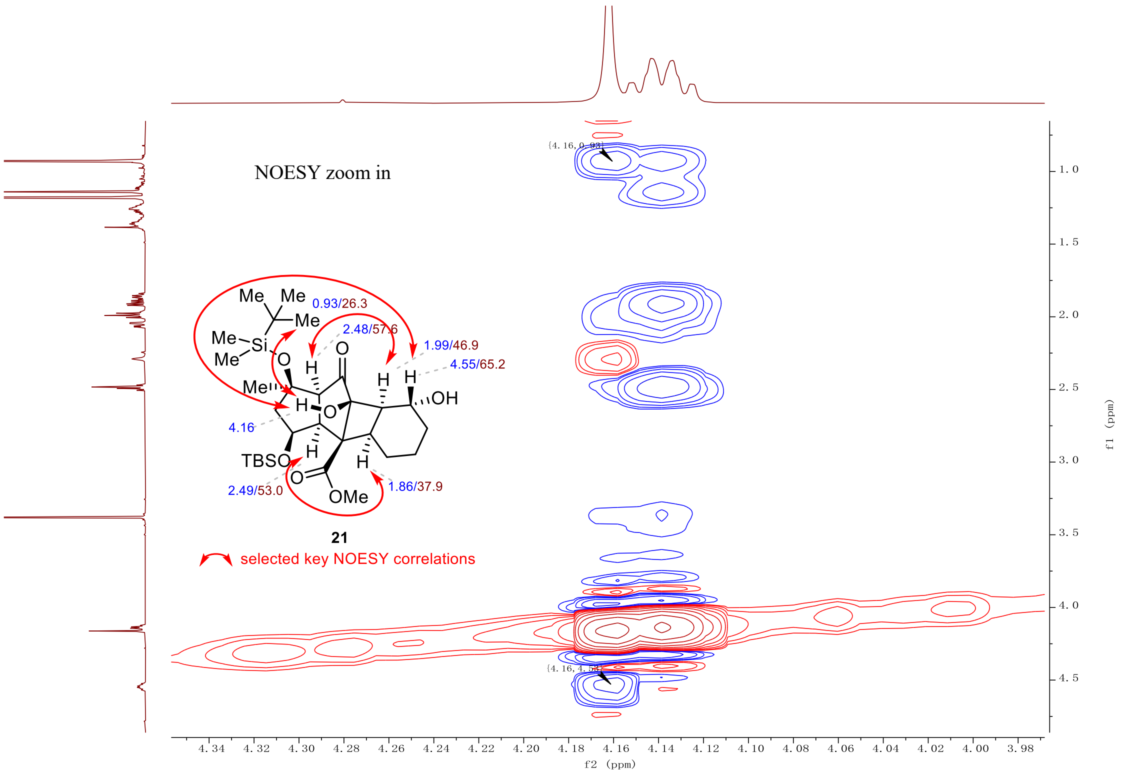
NOESY zoom in
0.93/26.3
2.48/57.6
1.99/46.9
4.55/65.2
4.16
2.49/53.0
1.86/37.9
21
selected key NOESY correlations
f2 (ppm)
f1 (ppm)

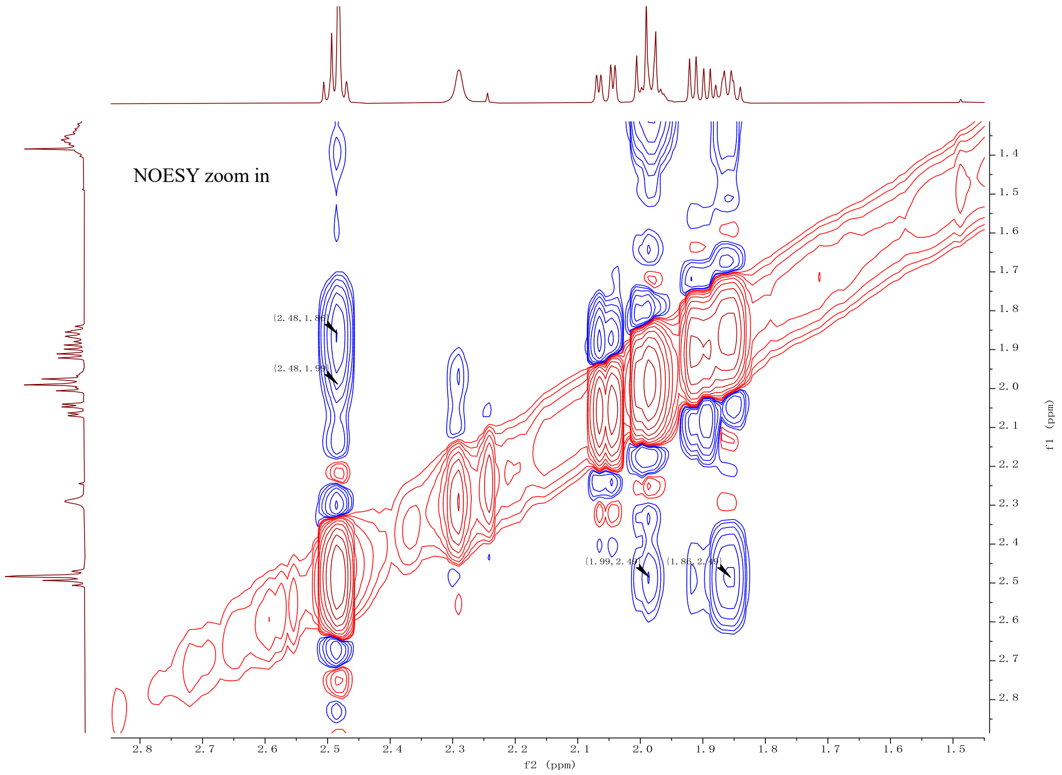
NOESY zoom in
f2 (ppm)
f1 (ppm)

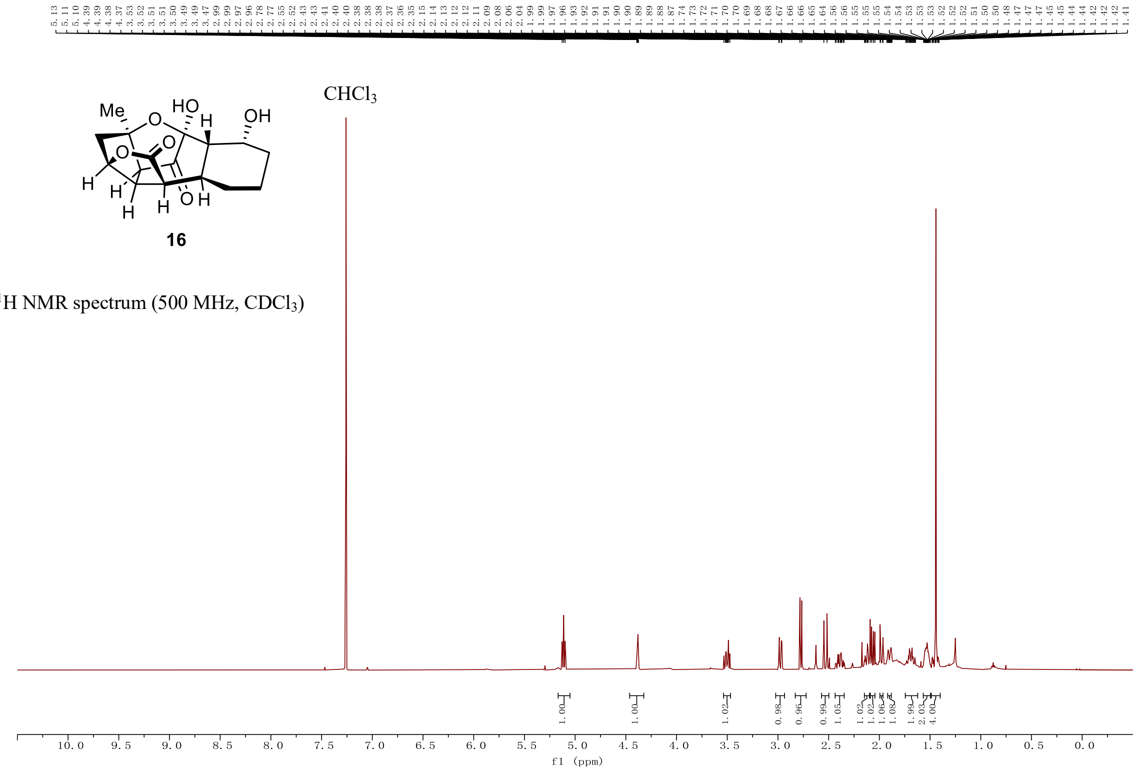
5.13 5.11 5.10 4.39 4.39 4.38 4.37 3.53 3.52 3.51 3.51 3.50 3.49 3.49 3.47 2.99 2.99 2.97 2.96 2.78 2.77 2.55 2.52 2.43 2.43 2.41 2.40 2.40 2.38 2.38 2.38 2.37 2.36 2.35 2.15 2.14 2.13 2.12 2.12 2.11 2.09 2.08 2.06 2.04 1.99 1.99 1.97 1.96 1.93 1.92 1.91 1.91 1.90 1.90 1.89 1.89 1.88 1.87 1.74 1.73 1.72 1.71 1.70 1.69 1.68 1.68 1.67 1.66 1.66 1.65 1.64 1.56 1.56 1.55 1.55 1.55 1.55 1.54 1.54 1.53 1.53 1.53 1.52 1.52 1.52 1.51 1.50 1.48 1.47 1.47 1.45 1.45 1.44 1.44 1.42 1.42 1.41
CHCl3
Me
O
HO
H
OH
O
O
H
H
H
H
OH
16
1H NMR spectrum (500 MHz, CDCl3)
1.00 1.00 1.02 0.98 0.96 0.99 1.05 1.02 1.02 1.06 1.08 1.99 2.03 4.00
10.0 9.5 9.0 8.5 8.0 7.5 7.0 6.5 6.0 5.5 5.0 4.5 4.0 3.5 3.0 2.5 2.0 1.5 1.0 0.5 0.0
f1 (ppm)

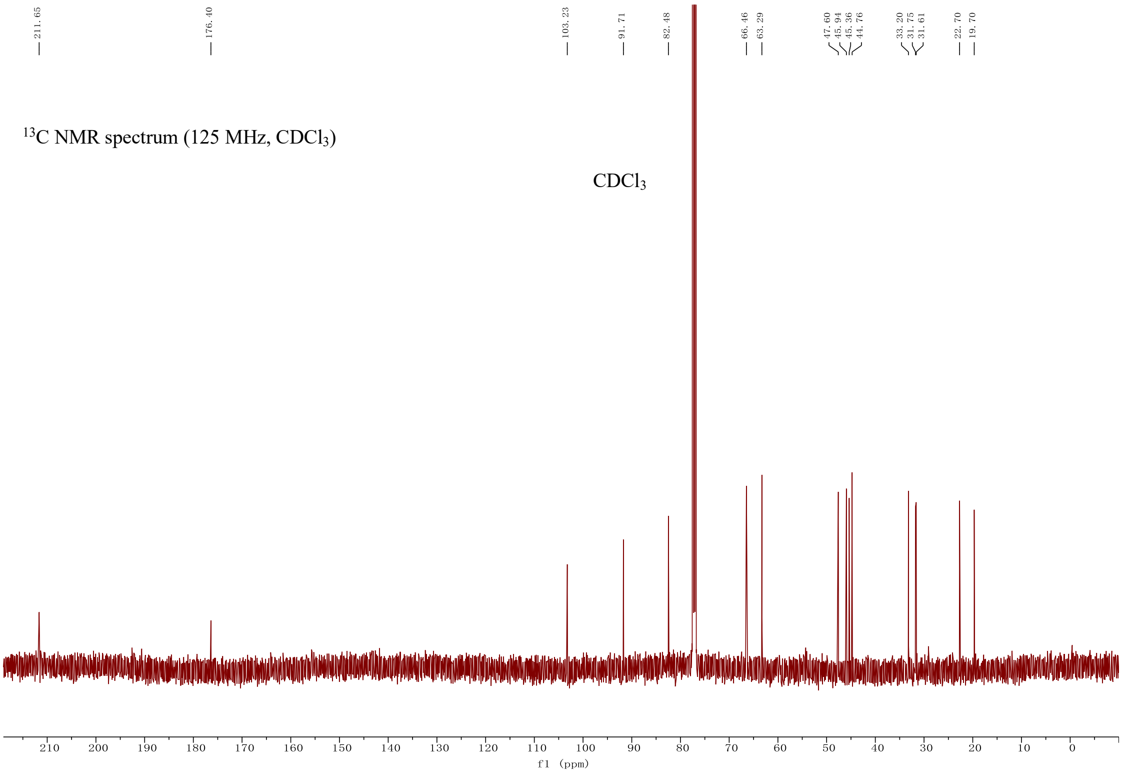
211.65
176.40
103.23
91.71
82.48
66.46
63.29
47.60 45.94 45.36 44.76
33.20 31.75 31.61
22.70
19.70
13C NMR spectrum (125 MHz, CDCl3)
CDCl3
210 200 190 180 170 160 150 140 130 120 110 100 90 80 70 60 50 40 30 20 10 0
f1 (ppm)

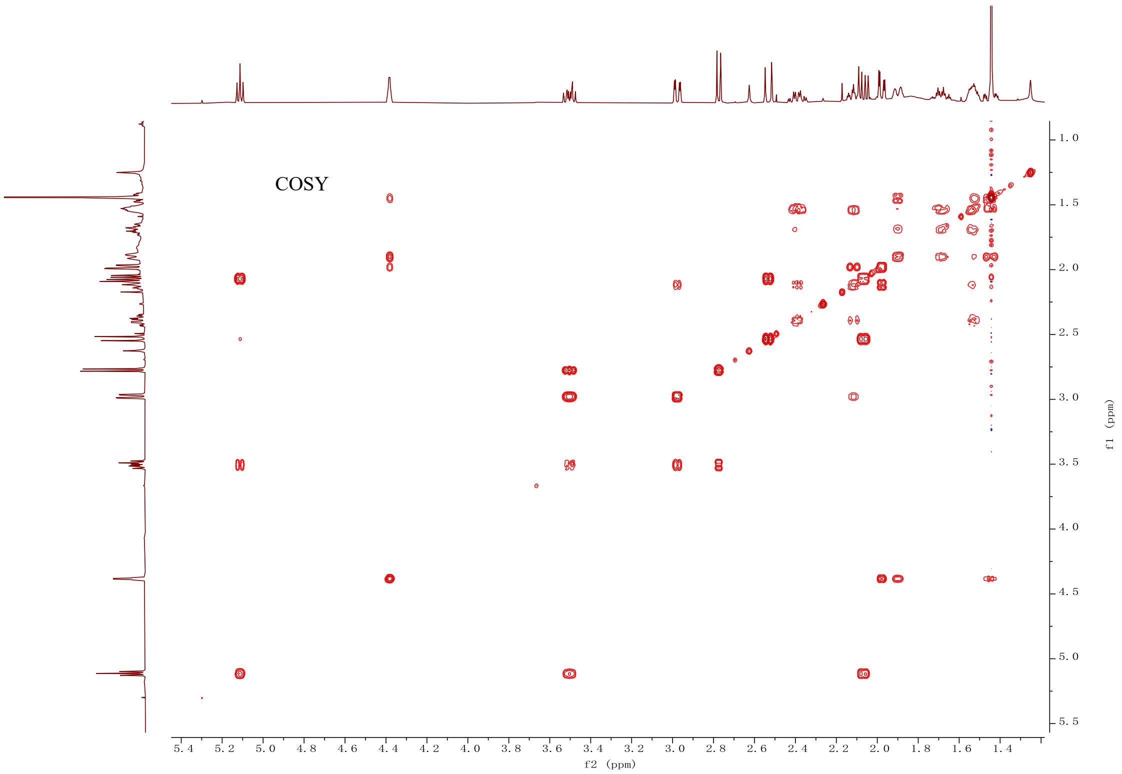
COSY
5.4 5.2 5.0 4.8 4.6 4.4 4.2 4.0 3.8 3.6 3.4 3.2 3.0 2.8 2.6 2.4 2.2 2.0 1.8 1.6 1.4
f2 (ppm)
1.0 1.5 2.0 2.5 3.0 3.5 4.0 4.5 5.0 5.5
f1 (ppm)

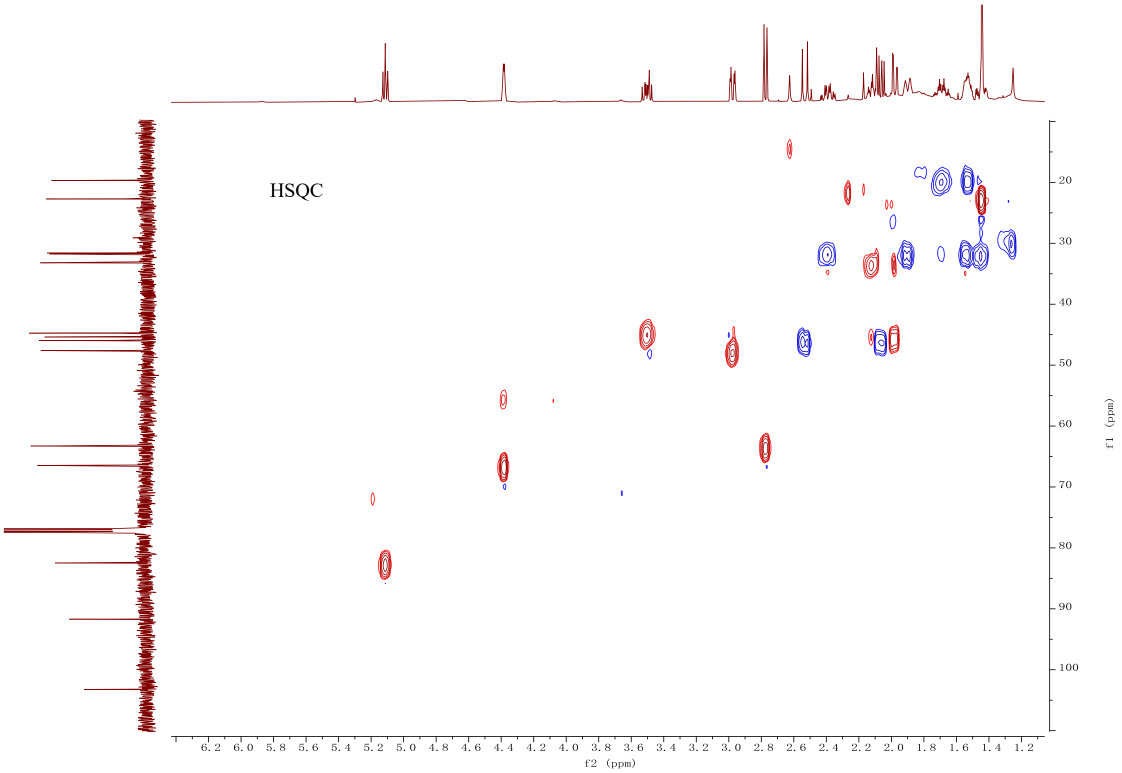
HSQC
6.2 6.0 5.8 5.6 5.4 5.2 5.0 4.8 4.6 4.4 4.2 4.0 3.8 3.6 3.4 3.2 3.0 2.8 2.6 2.4 2.2 2.0 1.8 1.6 1.4 1.2
f2 (ppm)
20 30 40 50 60 70 80 90 100
f1 (ppm)

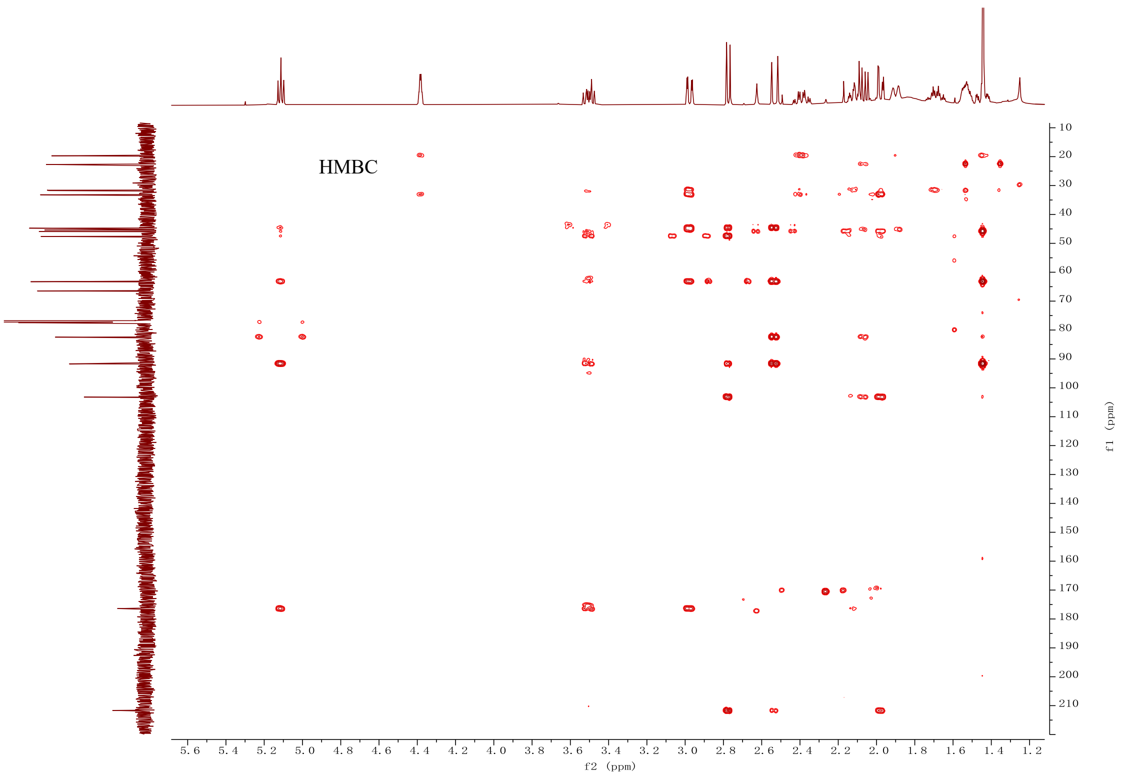
HMBC
f2 (ppm)
f1 (ppm)

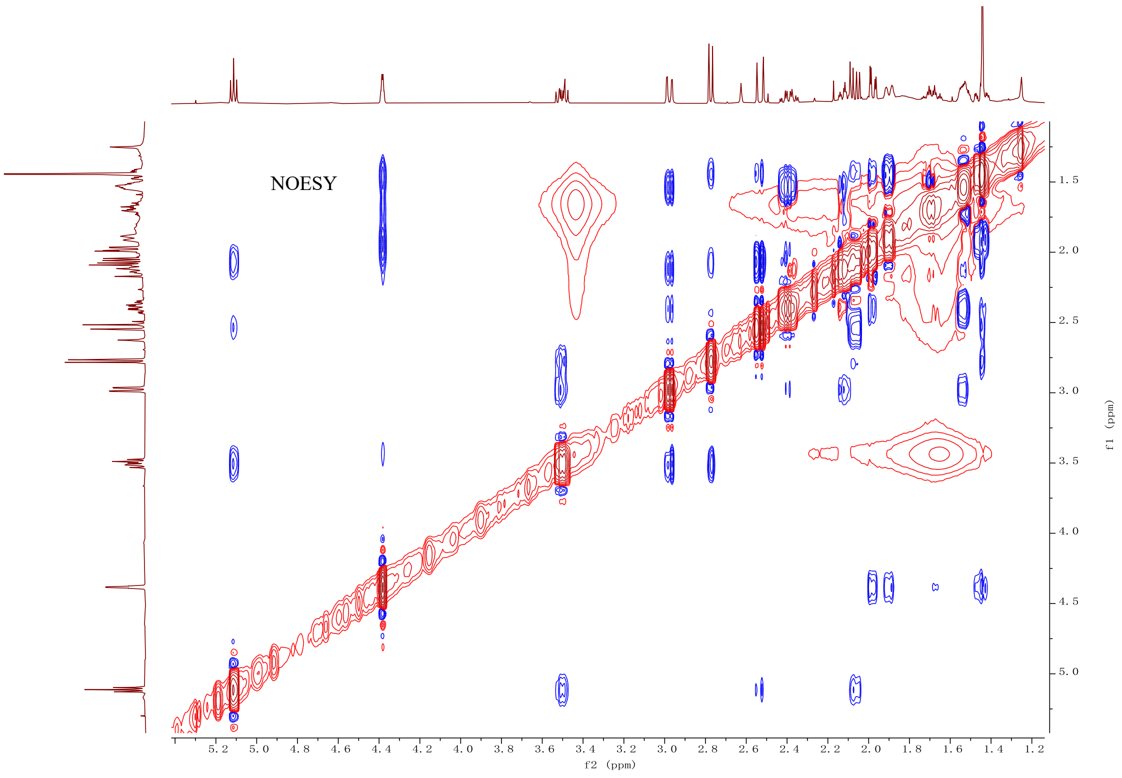
NOESY
f2 (ppm)
f1 (ppm)

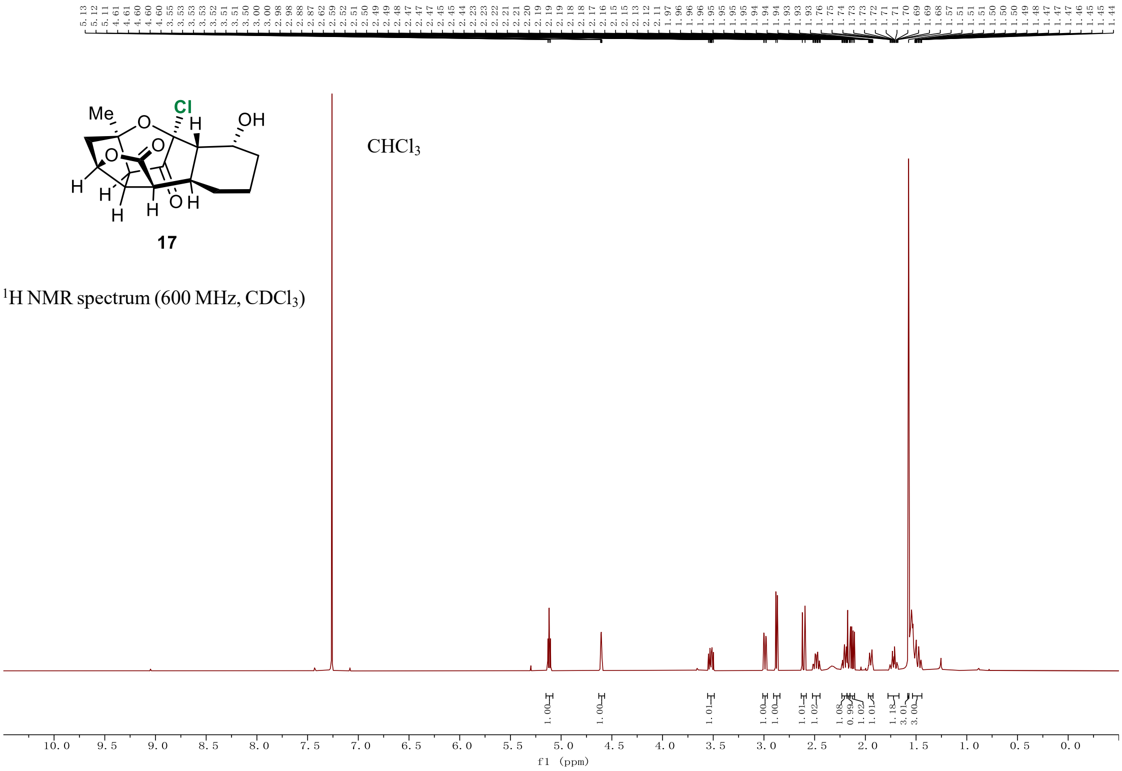

^{1}H NMR spectrum (600 MHz, $CDCl_3$)

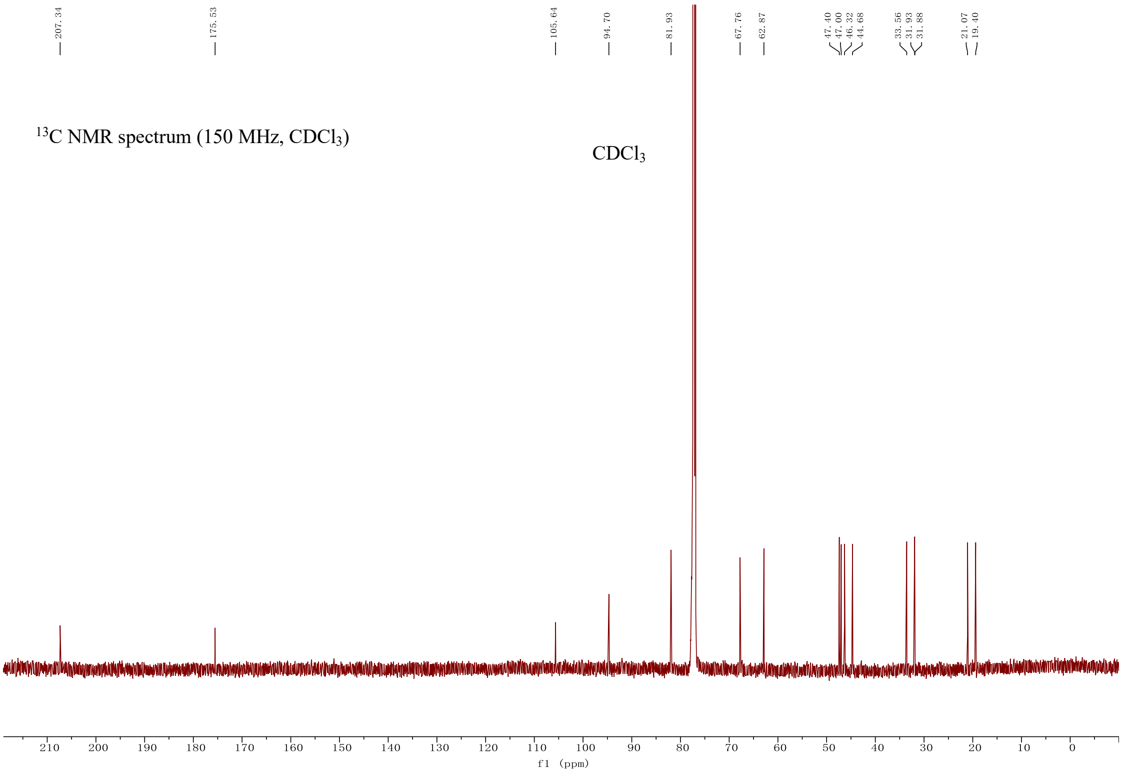

^{13}C NMR spectrum (150 MHz, $CDCl_3$)

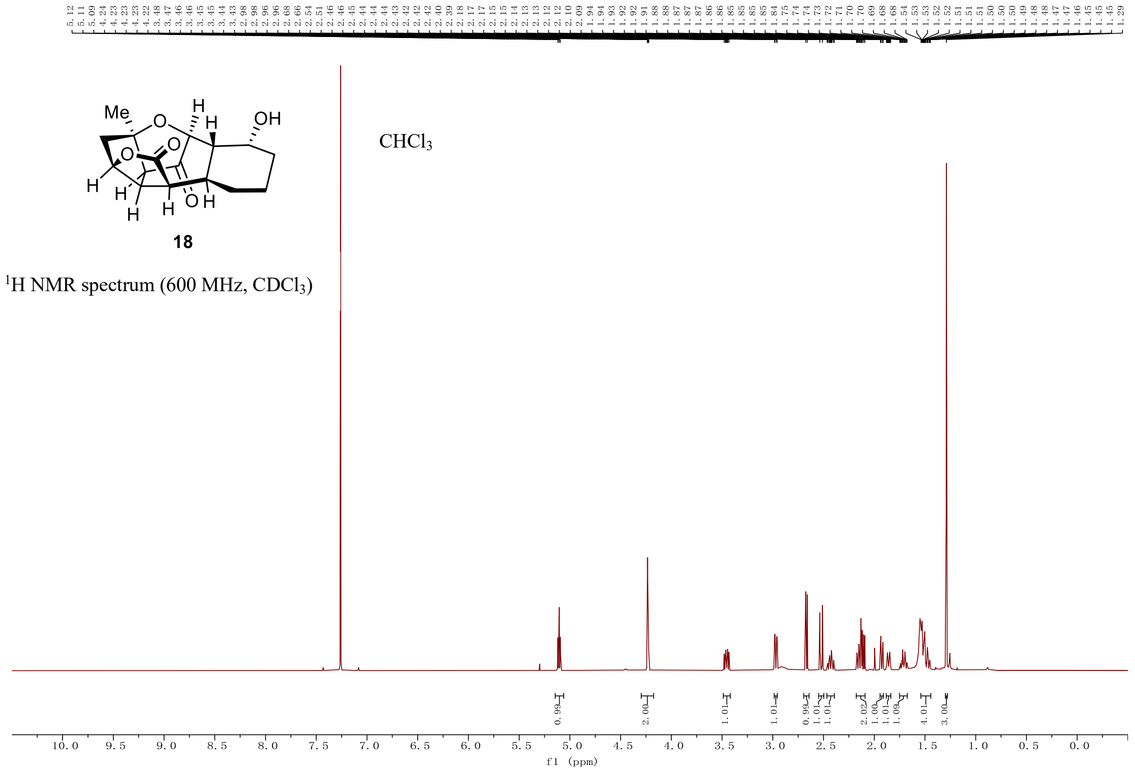
Me
O
H
H
OH
O
O
H
H
H
O
H
OH
18
CHCl3
1H NMR spectrum (600 MHz, CDCl3)
f1 (ppm)

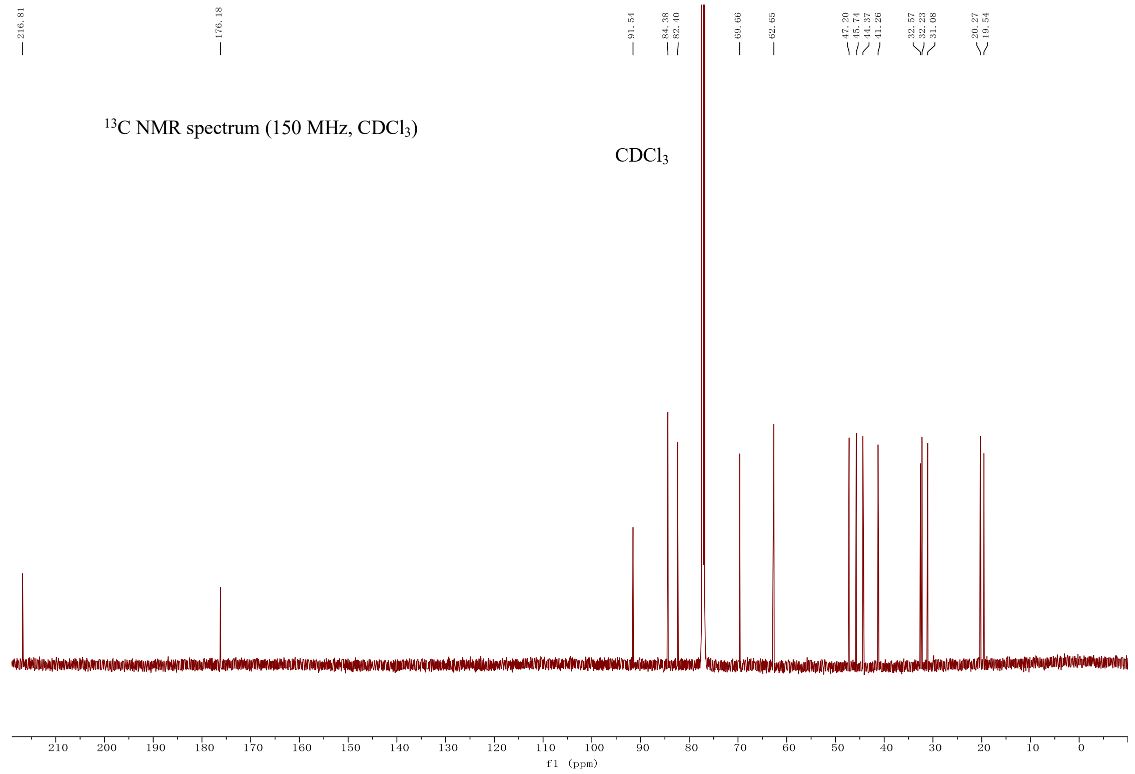
216.81
176.18
91.54
84.38
82.40
69.66
62.65
47.20
45.74
44.37
41.26
32.57
32.23
31.08
20.27
19.54
13C NMR spectrum (150 MHz, CDCl3)
CDCl3
f1 (ppm)

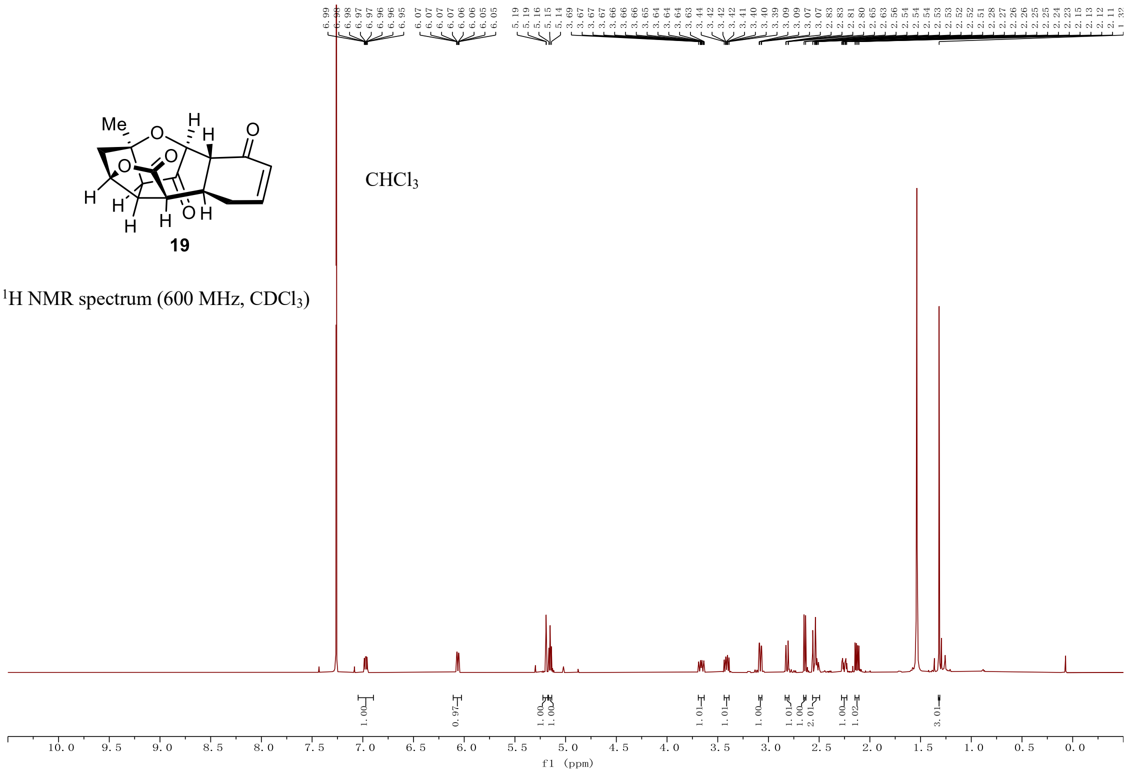

19

^{1}H NMR spectrum (600 MHz, $CDCl_3$)

^{13}C NMR spectrum (150 MHz, $CDCl_3$)

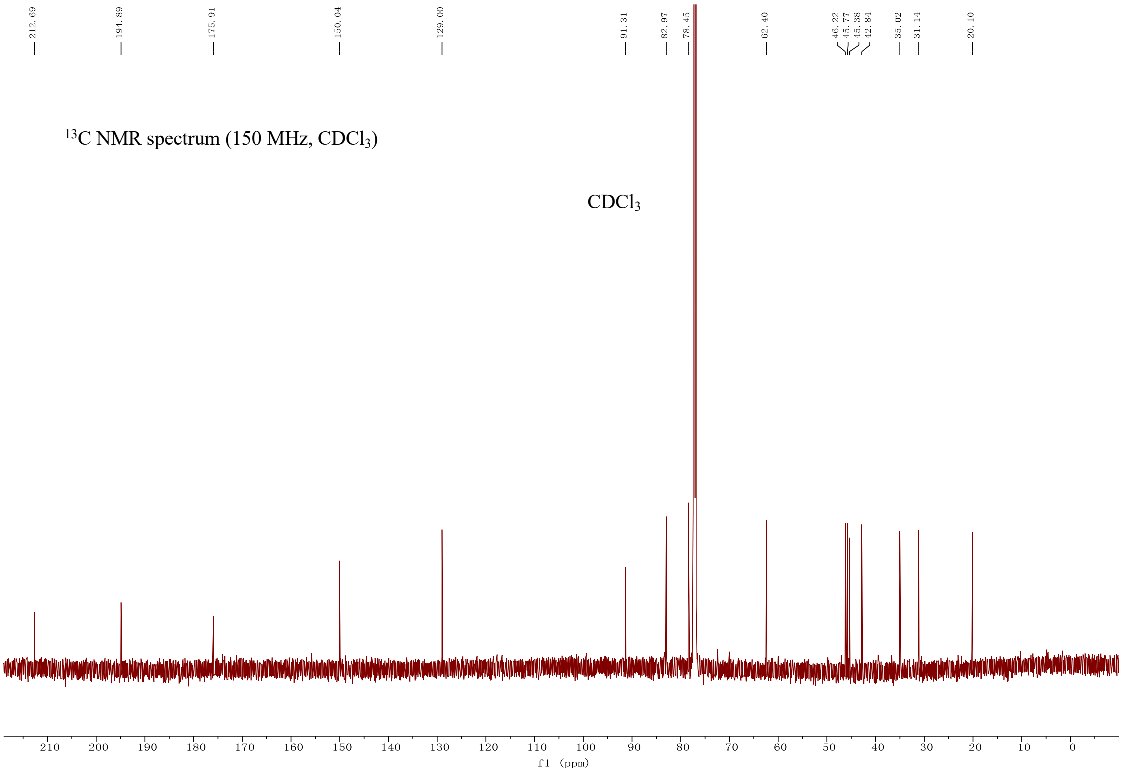

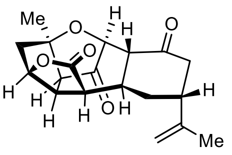

(+)-ineleganolide (**4**)

^{1}H NMR spectrum (600 MHz, $CDCl_3$)

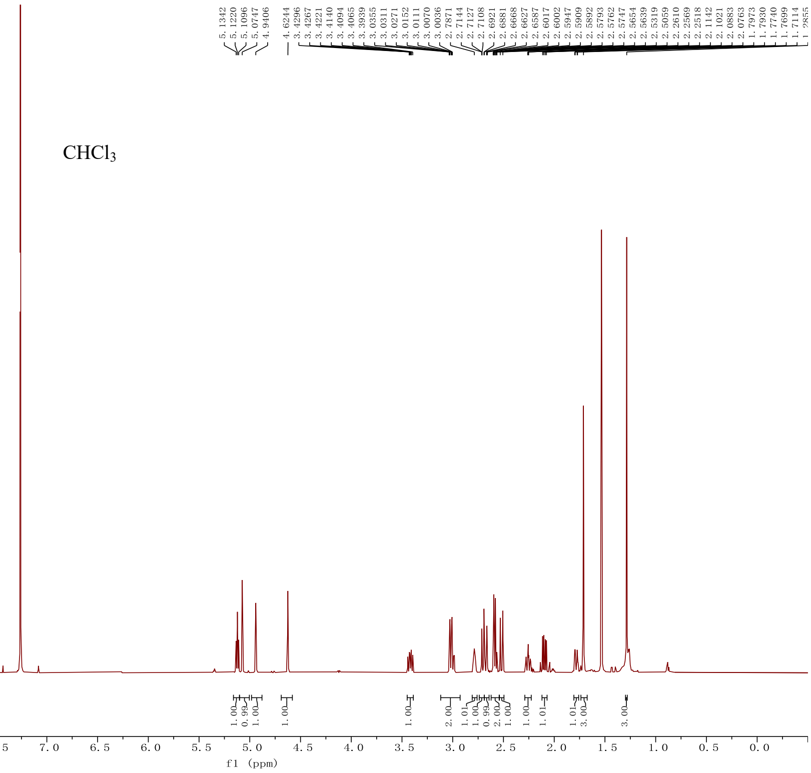

^{13}C NMR spectrum (150 MHz, $CDCl_3$)

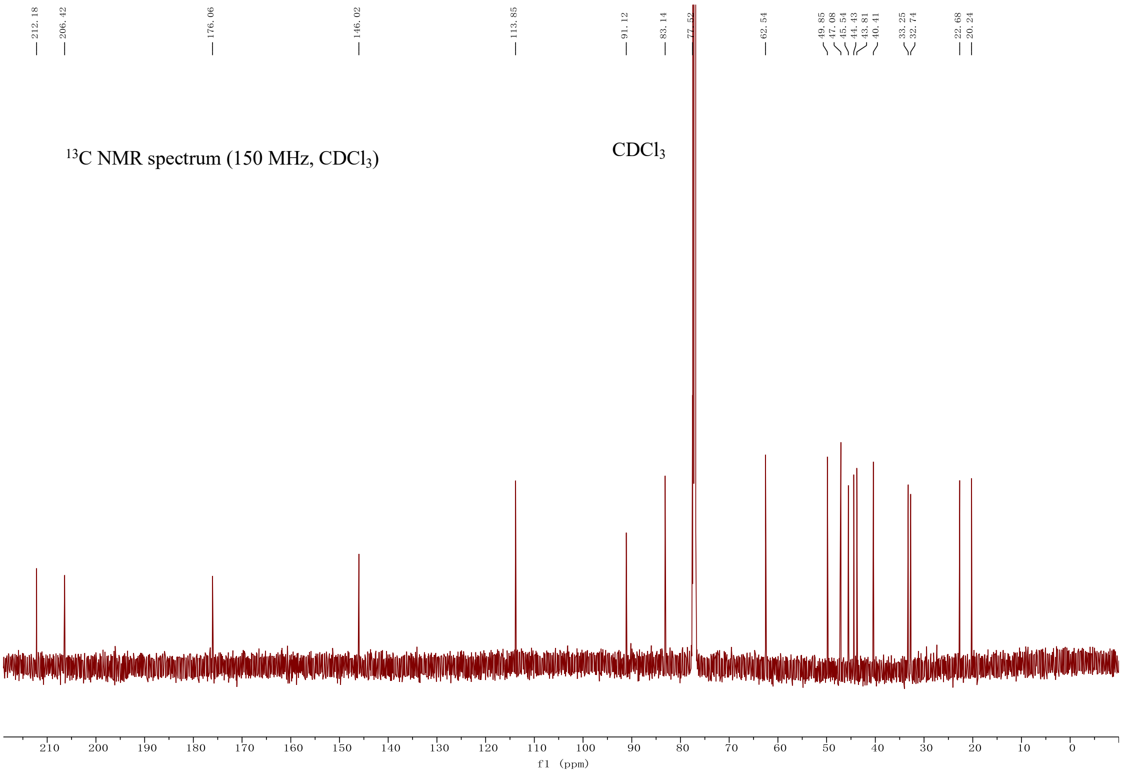

Supplement: Supplementary file 1 [file ja5c17640_si_001.pdf]
